# WTAP and m^6^A-modified circRNAs modulation during stress response in acute myeloid leukemia progenitor cells

**DOI:** 10.1007/s00018-024-05299-9

**Published:** 2024-06-23

**Authors:** Alessia Iaiza, Gilla Mazzanti, Frauke Goeman, Bianca Cesaro, Clelia Cortile, Giacomo Corleone, Claudia Tito, Francesca Liccardo, Luciana De Angelis, Vincenzo Petrozza, Silvia Masciarelli, Giovanni Blandino, Maurizio Fanciulli, Alessandro Fatica, Giulia Fontemaggi, Francesco Fazi

**Affiliations:** 1 Department of Biology and Biotechnology ‘Charles Darwin’, Sapienza University of Rome, P.le Aldo Moro, 5, 00185 Rome, Italy; 2 Section of Histology and Medical Embryology, Department of Anatomical, Histological, Forensic Medicine and Orthopedics Sciences, Sapienza University of Rome, Via A. Scarpa, 14-16, 00161 Rome, Italy; 3 SAFU, Department of Research, Diagnosis and Innovative Technologies, Translational Research Area, IRCCS Regina Elena National Cancer Institute, Rome, Italy; 4 Oncogenomic and Epigenetic Unit, IRCCS Regina Elena National Cancer Institute, Via Elio Chianesi 53, 00144 Rome, Italy; 5 Department of Medico-Surgical Science and Biotechnologies, Sapienza University of Rome, Latina, Italy

**Keywords:** circRNAs, WTAP, m^6^A, Oxidative stress, AML, circHIPK3

## Abstract

N^6^-methyladenosine (m^6^A) is one of the most prevalent and conserved RNA modifications. It controls several biological processes, including the biogenesis and function of circular RNAs (circRNAs), which are a class of covalently closed-single stranded RNAs. Several studies have revealed that proteotoxic stress response induction could be a relevant anticancer therapy in Acute Myeloid Leukemia (AML). Furthermore, a strong molecular interaction between the m^6^A mRNA modification factors and the suppression of the proteotoxic stress response has emerged. Since the proteasome inhibition leading to the imbalance in protein homeostasis is strictly linked to the stress response induction, we investigated the role of Bortezomib (Btz) on m^6^A regulation and in particular its impact on the modulation of m^6^A-modified circRNAs expression. Here, we show that treating AML cells with Btz downregulated the expression of the m^6^A regulator WTAP at translational level, mainly because of increased oxidative stress. Indeed, Btz treatment promoted oxidative stress, with ROS generation and HMOX-1 activation and administration of the reducing agent N-acetylcysteine restored WTAP expression. Additionally, we identified m^6^A-modified circRNAs modulated by Btz treatment, including circHIPK3, which is implicated in protein folding and oxidative stress regulation. These results highlight the intricate molecular networks involved in oxidative and ER stress induction in AML cells following proteotoxic stress response, laying the groundwork for future therapeutic strategies targeting these pathways.

**Supplementary Information:**

The online version contains supplementary material available at 10.1007/s00018-024-05299-9.

## Introduction

N^6^-methyladenosine (m^6^A) is a prevalent post-transcriptional RNA modification found in eukaryotic mRNAs, discovered in 1974 [1]. The enzyme responsible for this modification was purified in the 1990s [2], but only in the last decades, recent technological advances have significantly enhanced our understanding of m^6^A, revealing its dynamic and reversible nature [3–5]. The MAC complex, consisting of METTL3 and METTL14, catalyzes m^6^A modification and is regulated by the MACOM complex [6, 7]. Removal of m^6^A is mediated by the FTO and ALKBH5 demethylases, both belonging to the ALKB subfamily of the superfamily of Fe(II)/2-oxoglutarate dioxygenases [8, 9]. Specific “readers”, such as members of the YT521-B homology (YTH) domain family [10] and certain members of the heterogeneous nuclear ribonucleoprotein (HNRNP) family [11–13], recognize and read the m^6^A modification. Recently, IGF2BPs have also been found to recognize this modification [14]. m^6^A modification modulates RNA metabolism, including structure, maturation, stability, splicing, export, translation and decay, affecting different biological processes, as well as hematopoiesis [15–17]. Acute myeloid leukemia (AML) is a malignancy characterized by the uncontrolled accumulation of incompletely differentiated myeloid cells, impacting innate immunity. With an annual incidence of 3.8 cases per 100,000 in the US and Europe [18], AML is particularly challenging, with less than 5% 5-year overall survival in patients older than 65 years [19], although it can also affect adults and children [20]. One of the prevalent genetic abnormalities in acute myeloid leukemia is the Internal Tandem Duplication of FLT3 gene, known as FLT3-ITD. This mutation in the self-inhibitory domain of FLT-3 leads to constitutive activation, hindering receptor regulation [21]. The FLT3-ITD mutated protein is improperly processed in the ER, leading to its retention in this organelle as a misfolded protein. Furthermore, this mutation causes elevated reactive oxygen species (ROS), DNA double-strand breaks (DSBs), and repair errors, contributing to AML aggressiveness in FLT3-ITD+ patients [21, 22]. This and other genetic mutations [23, 24], along with misfolding and mutant proteins, induce continuous Endoplasmic Reticulum (ER) stress and activate the Unfolded Protein Response (UPR) [25]. Leukemic cells adeptly manage ER stress and sustained UPR, evading cell death and promoting growth [26]. One strategy to exacerbate ER stress and induce AML cell death is to inhibit ER stress-associated degradation (ERAD), which allows the removal and degradation of misfolded proteins accumulated in the ER [27]. Bortezomib, also known as Velcade, is the first selective proteasome inhibitor that demonstrated significant preclinical activity in several tumor models [28]. Moreover, it has been successfully used in patients with refractory or relapsed multiple myeloma, either as a single agent or combined with other therapies [29]. Recent studies demonstrated the potential use of Btz against AML [30–32]. Additionally, recent insights highlighted the involvement of circRNAs (single-stranded circularized RNA products of back-splicing) in hematopoietic malignancies [33–35]. As m^6^A modification can affect circRNA biogenesis, translation, degradation, and cellular localization [36, 37] its role in these processes is significant. Further studies have demonstrated the correlation between m^6^A modification and various cellular stress types. In particular, it has been shown that ROS levels can regulate m^6^A enzymes and, conversely, m^6^A enzymes can control redox homeostasis, for example, by influencing oxidative stress-related signaling pathways [38–42]. In light of what has been described thus far, our research is focused on investigating the dynamic interplay among m^6^A modification, circRNAs, and proteostasis within the landscape of AML. This multifaceted perspective unravels the complexities of this prevalent blood malignancy and sheds light on the potential role of m^6^A-modified circRNAs in AML progression and resistance to treatments.

## Materials and methods

### Cell culture and treatments

MOLM-13 cell line was cultured with RPMI 1640 (Gibco^®^ Thermo Fisher Scientific, Waltham, MA USA) enriched with pen/strep (50 U/mL) and with 10% heat-inactivated South-American Fetal Bovine Serum (FBS) (Gibco^®^ Thermo Fisher Scientific), at 37 °C and 5% CO_2_.

MOLM-13 cells were treated with 5 nM and 10 nM Bortezomib (Btz, Sigma-Aldrich) for 6 h, 16 h and 24 h. Regarding the treatment with N-acetylcysteine (NAC, Sigma-Aldrich), cells were pre-incubated with vehicle (sterilized water, indicated as NIL) or with NAC 20 mM for 24 h at pH 7.4 and then treated with 5 nM of Btz for 16 h.

MOLM-13 cell line resistant to Btz was established by exposing MOLM-13 cells to the continuous presence of stepwise increasing levels of Btz. After 4 months, we seeded them in a 96-well plate and proceeded with the clone selection method. Then we tested the clones and selected those that showed a 50% cell death rate with the higher concentration of Btz, 10 nM, after 72 h of treatment.

MOLM-13 cells were treated with puromycin (#ant-pr-1, InvivoGen) at 10 μg/mL after 16 h of Btz treatment. Incorporated puromycin protein level was then analyzed at 0, 10 and 30 min after treatment by western blot analysis.

For the protein stability assay, untreated and 5 nM Btz-treated cells for 16 h were stopped, the medium containing the drug removed, and then treated with 10 μg/mL cycloheximide (CHX, Sigma-Aldrich) for 1 and 3 h.

### Cell death, cell cycle and ROS detection

Cell death and cell proliferation were assessed by flow cytometry upon staining with propidium iodide (Sigma-Aldrich).

For cell cycle analysis, 2 × 10^5^ cells were fixed O/N in 70% ethanol, then incubated with 50 μg/ml propidium iodide (Sigma-Aldrich) and 50 units/mL DNase-free RNase A for 3 h (Sigma-Aldrich) before analysis by flow cytometry (1 × 10^4^ events).

ROS levels were measured by flow cytometry after staining cells with the redox-sensitive dye 2 μM CM-H2DCFDA (ThermoFisher Scientific) in pre-warmed PBS at 37 °C for 30 min in the dark.

### Lentiviral transduction

Lentiviral constructs for circHIPK3 knockdown experiments were obtained from circHIPK3 TRC2 pLKO.5-PURO (shcircHIPK3, Sigma-Aldrich) and SHC202 TRC2 (Non-Target shRNA Control, shSCR, Sigma-Aldrich) was used as control. For viral transduction, MOLM-13 cells (1 × 10^6^ cells) were resuspended in 1 mL of serum-free and antibiotic-free media supplemented with 4 µg/mL Polybrene (Sigma-Aldrich). Subsequently, cells were infected with 2 μL of lentiviral particles previously resuspended in HBSS buffer. After 6 h, an equal volume of medium with 2× serum and 2× antibiotic concentrations were added. At 24 h post-viral transduction, the medium was replaced with complete medium. 48 h after viral transduction, cells were subjected to selection with 2 µg/mL of puromycin (Sigma-Aldrich) until resistant colonies became visible (3–5 days).

### RNA extraction and real-time qRT-PCR analysis

Total RNA was extracted using the TRIzol RNA Isolation System (Invitrogen) according to manufacturer instructions. Reverse transcription to cDNA was performed with the High-Capacity RNA-to-cDNA Kit (Applied Biosystems) and cDNA was amplified using the SYBR™ Green PCR Master Mix and TaqMan Universal PCR Master Mix (Thermo Fisher Scientific) on an ABI PRISM 7500 Sequence Detection System (Applied Biosystems). The following oligo sequences were used:

**Table:** 

Target		Sequence oligo
WTAP	SYBR	For 5′-GGCGAAGTGTCGAATGCT-3′ Rev 5′-CCAACTGCTGGCGTGTCT-3′
METTL3	SYBR	For 5′-AAGCAGCTGGACTCTCTGCG-3′ Rev 5′-GCACTGGGCTGTCACTACGG-3′
FTO	SYBR	For 5’-TGGGTTCATCCTACAACGG-3’ Rev 5’-CCTCTTCAGGGCCTTCAC-3’
YTHDC1	SYBR	For 5’-CCTACGCCAGATGGTTCTGAG-3’ Rev 5’-CATTCTCAGTGTTGTTCCCTTGC-3’
circZNF609	SYBR	For 5′-AAACCGGAGCCAGAGGAAGG-3′ Rev 5′-CAGCTATGTTCTCAGACCTGC-3′
circRNF220	SYBR	For 5′-TCTGATCGGGAAGCCTCATC-3′ Rev 5′-TCGGAGTCTCTTTCTGTGGC-3′
circHIPK3	SYBR	For 5′-CGGCCAGTCATGTATCAAAGA-3′ Rev 5′-ACAACTGCTTGGCTCTACTTTG-3′
circAFF1	SYBR	For 5′-CCTGAACTGAAACCACTGCC-3′ Rev 5′-CCTGGTTGCGTCTTTCCTTC-3′
ZNF609	SYBR	For 5′-AAGGCTCAACTCCCTCACTC-3′ Rev 5′-GTGGAAACGGCAAACAGGAT-3′
RNF220	SYBR	For 5′-CACCTCCACCCTCAATTTGC-3′ Rev 5′-CAAGTGGGGAGACTCTGTGT-3′
HIPK3	SYBR	For 5′-GTTGTGATACGGTGGATGGC-3′ Rev 5′-GGCACCACAACAGAACAGAC-3′
AFF1	SYBR	For 5′-TACAATGACGACAGAAACCTGC-3′ Rev 5′-GGCGATGAGTGTGAGACTTAGTA -3′
hBiP	SYBR	For 5′-TAGCGTATGGTGCTGCTGTC-3′ Rev 5′-TTTGTCAGG GGTCTTTCACC-3′
XBP1s	SYBR	For 5′-GAGTCCGCAGCAGGTGC-3′ Rev 5′-TCCTTCTGGGTAGACCTCTGGGAG-3′
H3	SYBR	For 5′-GTGAAGAAACCTCATCGTTACAGGCCTGGT -3′ Rev 5′-CTGCAAAGCACCAATAGCTGCACTCTGGAA -3′
GAPDH	TaqMan	Primer 1 5′-ACATCGCTCAGACACCATG-3′ Primer 2 5′-TGTAGTTGAGGTCAATGAAGGG-3′
HMOX-1	TaqMan	Primer 1 5′-TCATGAGGAACTTTCAGAAGGG-3′ Primer 2 5′-TGCGCTCAATCTCCTCCT-3′

### Protein lysate and immunoblotting analysis

Cells were lysed with RIPA buffer and fresh protease inhibitors. Cells were sonicated for 10 s and centrifuged for 10 min at 12,000× rpm. Lysates were quantified using Bradford Assay Reagent (#1863028, Thermo Fisher, USA). Protein samples were separated by SDS-PAGE and transferred onto a nitrocellulose membrane. The membrane was incubated overnight with the following primary antibodies: rabbit monoclonal anti-METTL3 (#ab195352, Abcam); mouse monoclonal anti-WTAP (#60188-1-Ig, Proteintech); rabbit monoclonal anti-YTHDC1 (#ab122340, Abcam); rabbit monoclonal anti-FTO (#27226-1-AP, Proteintech); mouse monoclonal anti-BiP/GRP78 (#610978, BD); rabbit monoclonal anti-Phospho-eIF2α (Ser51) (D9G8,#3398, Cell Signaling); rabbit monoclonal anti-eIF2α (D7D3,#5324, Cell Signaling);rabbit monoclonal anti-H3 (#ab1791, Abcam); mouse monoclonal anti-Puromycin, clone 12D10 (#MABE343, Millipore); mouse monoclonal anti-GAPDH 6C5 (#sc32233, Santa Cruz Biotechnology); mouse monoclonal anti-Tubulin B512 (#T5168, Sigma-Aldrich). For the detection we used ECL method (Enhanced ChemiLuminescence) (Amersham Biosciences) using the ChemiDoc-It Imaging System (UVP, Upland, CA) instrument.

### Immunofluorescence

10^6^ CTR and Btz treated cells were collected and stained with Cytofix/Cytoperm Fixation/Permeabilization solution Kit (#BD554714, Becton Dickinson) according to the manufacturer’s instructions. Cells were fixed in Fix Perm buffer for 20 min at 4 °C, rinsed with Wash Perm buffer and incubated with the following primary antibodies: anti-m^6^A (#ab151230, Abcam); anti-GM130 (#610822, BD). After two Wash Perm washes, cells were incubated with the secondary antibody, Alexa Fluor 488-labeled goat anti-rabbit (#A-11034, Invitrogen) or Alexa Fluor 555-labeled goat anti-mouse (#A-21424). The nuclei were stained with Hoechst 33342 (Life Technologies) for 5 min in Wash Perm. Cells were rinsed, seeded on the slides (200,000 cells) and mounted in VECTASHIELD antifade mounting media (#H-1000, Vector Laboratories). Images were acquired using a Zeiss LSM 900 confocal laser scanning microscope.

### m^6^A immunoprecipitation

Cells were treated with 5 nM of Btz and total RNA was extracted after 16 h. The samples were treated with RNAseR, in order to remove all the linear RNA. A fraction of the RNA was collected as input sample and the rest was incubated with an m^6^A-specific antibody (#ab151230, Abcam) or with a control rabbit IgG (Millipore) antibody for 2 h at 4 °C on a rotator. Immunoprecipitated RNA was eluted and resuspended in RNase-free water. The same procedure, but without RNAseR treatment was performed on new biological replicates, to validate the RNAseq data.

### CircRNAs sequencing

The purified RNA was quantified with NanoDrop. The size distribution of the RNA was controlled on a Bioanalyzer with the Agilent RNA 6000 Pico Kit (Agilent Technologies, Santa Clara, CA, USA). The RNA libraries for sequencing were prepared with the SMARTer Stranded Total RNA Sample Prep Kit—Low Input Mammalian without ribosomal depletion (Takara Bio. USA, Inc). The quality of the final libraries was controlled on a Bioanalyzer using the High Sensitivity DNA Kit (Agilent Technologies, Santa Clara, CA, USA). The quantification of the libraries was performed by qPCR. Sequencing was carried out on a NovaSeq 6000 instrument (Illumina Inc., San Diego, CA, USA), sequencing in paired-end mode 101 bp. circRNA expression values are deposited in Gene Expression Omnibus (GEO) database with accession number GSE262829.

### Computational analysis of circRNA-seq

The quality assessment of circRNA-seq was evaluated through FASTQC tool v0.11.9 (http://www.bioinformatics.babraham.ac.uk/projects/fastqc/) for each sample. Additionally, a comprehensive analysis of sequencing quality was performed by combining the results of FASTQC with the multiqc too v.1.9 [43]. Back spliced junctions (BSJ) were identified and aligned to the reference genome (hg19) using STAR v2.7.9a [44] (parameters for BSJ: –chimJunctionOverhangMin 15, –chimOutType Junctions SeparateSAMold, –chimScoreMin 15, –chimScoreSeparation 10, –chimSegmentMin 10). The sorted alignments were indexed using the *index* function of samtools v1.9 [45]. Subsequently, the *parse* module of CIRCExplorer2 v.2.3.8 software [46] (parameter: -t STAR) was used to extract the coordinates of fragments mapping to BSJ sites. These were annotated through the *annotate* module of CIRCExplorer2 based on a comprehensive gene annotation of hg19 (https://www.gencodegenes.org/human/release_19.html). The quantification of detected circRNA for each sample was performed with CIRIquant toolkit v.1.1.2 [47] by taking FASTQ files and the coordinates of BSJ sites as input. In both control and bortezomib-treated samples of circRNA with m^6^A modification, the quantification was corrected respectively on the total amount of circRNA before and after treatment. The per-sample control and bortezomib-treated m^6^A circRNA quantification results were merged into a single matrix using the prep_CIRIquant module of CIRIquant v.1.1.2. This matrix contains the expression levels of m^6^A circRNAs (n = 9723) detected in all samples. The raw counts of identified m^6^A circRNAs within the matrix were normalized using a multi-step procedure integrating DGEList, filterByExpr, calcNormFactors, and estimateDisp functions from the EdgeR v. 3.38.4 R package [48]. The normalized matrix was converted into counts per million (CPM). The CPM matrix was refined by retaining m^6^A circRNAs with an average CPM value greater than 0.1 across all samples (n = 2422). The refined data were scaled and subjected to clustering analysis for detecting m^6^A circRNA sets that exhibited specific expression profile across the control and bortezomib-treated groups of m^6^A circRNA samples. The clustering analysis and the visualization of results was obtained using an in-house R script leveraging ComplexHeatmap R package v. 2.12.1. Clustering group assignments for each m^6^A circRNA sample were stored in Supplementary Table 1. The identification of m^6^A circRNA sets was performed using the ward.2 hierarchical clustering method and the similarity between m^6^A circRNA enrichment among the samples was computed using the Manhattan distance metric. Concurrently, the samples were clustered according to ward. D2 hierarchical clustering and the similarity between samples was computed using the maximum distance. From the clustering analysis results, we selected m^6^A circRNAs (n = 126) that are uniformly highly expressed in the bortezomib condition group compared to the control group. The enrichment of these selected m^6^A circRNAs was illustrated in a heatmap generated using the ComplexHeatmap R package v. 2.12.1, with the same clustering and distance method previously set.

### Statistical analysis

The statistical analyses were performed using GraphPad Prism (GraphPad Software, San Diego, California, USA). Statistical analysis to determine significance was performed using Student’s t-tests or the One-Way Anova test. Differences were considered statistically significant at the p < 0.05 level.

## Results

### Btz treatment induces downregulation of m^6^A enzymes expression at post-transcriptional level and downregulation of global m^6^A modification levels

**Fig. 1:**
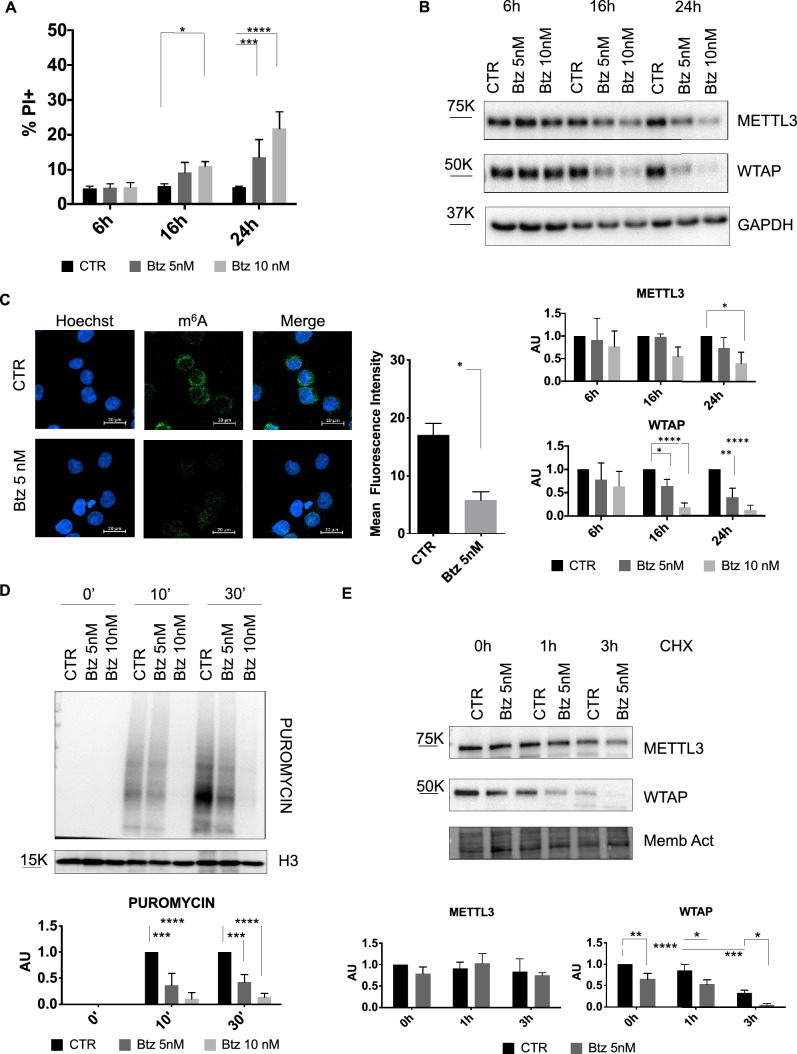
**A** Percentage of cell death detected by propidium iodide (PI) after 6 h, 16 h and 24 h of treatment with Btz 5 nM and 10 nM (n = 4). *P ≤ 0.05; ***P ≤ 0.0005; ****P ≤ 0.00005; statistical analysis was performed by Two-Way ANOVA. **B** Representative western blot of METTL3 and WTAP and relative quantification after 6 h,16 h and 24 h of treatment with Btz 5 nM and 10 nM (n = 4). *P ≤ 0.05; **P ≤ 0.005; ****P ≤ 0.00005; statistical analysis was performed by Two-Way ANOVA. **C** MOLM-13 cells were treated with 5 nM of Btz for 16 h, labeled with Hoechst (cell nuclei) and with anti-m^6^Ab labeled with Alexa Fluor 488 (m^6^A modification). Scale bars, 20 μm. On the right, graph of mean fluorescence quantification (n = 2). ***P ≤ 0.0005; statistical analysis was performed by T-Test. **D** Detection by western blot analysis of puromycin incorporation after 0′, 10′ and 30′ in control cells and in cells treated with Btz 5 nM for 16 h. On the bottom is presented the quantification relative to the control for each time point (n = 3). **E** Representative western blot analysis of MOLM-13 cells treated with 5 nM of Btz for 16 h and subsequently with CHX for additional 0 h (CHX -), 1 h and 3 h. On the bottom, relative quantification of METTL3 and WTAP protein levels, normalized on total protein measured by stain-free BioRad method (n = 3). *P ≤ 0.05; **P ≤ 0.005; ***P ≤ 0.0005; ****P ≤ 0.00005; statistical analysis was performed by Two-Way ANOVA

Considering m^6^A RNA modification’s roles in circRNA biogenesis and function and the involvement of cellular stress in malignant transformation, tumor progression, and drug resistance [36, 49], we decided to investigate the role of cellular stress induced by Btz on the expression m^6^A regulators. Based on literature data and our analysis of dose–response curves, we decided to use two different doses of Btz at short times [30, 32]. We treated MOLM-13, a FLT3-ITD+ human AML cell line, with 5 nM and 10 nM of Btz for 6 h,16 h and 24 h to induce acute stress. Cytofluorimetric analysis of cell death showed that Btz treatment resulted in low cell death rates at the indicated times and concentrations, with only 25–30% dead cells in the samples treated with the highest dose for 24 h (Fig. 1A). Then, we investigated the expression levels of some proteins involved in m^6^A RNA modification under acute stress conditions in AML. Specifically, we analyzed the expression of the methyltransferase METTL3 and the cofactor WTAP, which is critical for METTL3 activity. As shown in Fig. 1B, we observed a general downregulation of both proteins, METTL3 and WTAP, upon Btz treatment after 24 h. Interestingly, a strong and significant decrease in WTAP protein expression levels was observed as early as 16 h of treatment with the lowest Btz concentration, 5 nM. We additionally explored the expression levels of the YTHDC1 and FTO proteins. The former plays a crucial role in leukemogenesis and the maintenance of myeloid cell state, while the latter is implicated in various drug resistance mechanisms [50–52]. Similarly to WTAP and METTL3, Btz treatment induced a significant strong downregulation of YTHDC1 and a modest decrease of FTO protein expression levels, as depicted in Supplementary Fig. 1A. To understand whether this regulation was transcriptional or post-transcriptional, we further evaluated METTL3 and WTAP mRNA levels by RT-qPCR. As shown in Supplementary Fig. 1B, the reduction in protein levels did not correspond to a decrease in their mRNA, suggesting post-transcriptional regulation of METTL3, WTAP, YTHDC1, and FTO upon Btz treatment. Since the decrease in protein levels of m^6^A regulators, we investigated the effect of Btz-induced stress on overall m^6^A modification levels. To address this question, we treated the MOLM-13 cell line with 5 nM of Btz for 16 h and analyzed the amount of m^6^A through immunofluorescence with an anti-m^6^A antibody, quantifying the fluorescence intensity. The results illustrated in Fig. 1C showed reduced fluorescence intensity in the Btz-treated cells, indicating a decrease in RNA methylation level.

### Btz treatment induces inhibition of global translation and WTAP degradation

According to literature data, intrinsic and extrinsic conditions like ER stress, hypoxia, oxidative stress, and proteasome inhibition lead to phosphorylation of eIF2α, resulting in the inhibition of global protein synthesis [53]. For this reason, we wondered whether the reduced quantity of the m^6^A regulators was due to a decrease in global protein synthesis. We employed the SUnSET method, using an antipuromycin antibody to detect changes in protein synthesis [54]. Specifically, control cells and those treated with 5 nM and 10 nM of Btz were exposed to puromycin for 0, 10 and 30 min, followed by the analysis of newly synthesized polypeptides by western blotting. As shown in Fig. 1D, we observed a significant drop in puromycin incorporation upon Btz treatment, particularly evident with the highest concentration compared to control cells, indicating a global reduction of protein synthesis. This finding aligns with the decline in protein levels of the m^6^A regulators after 24 h treatment with Btz 10 nM. To understand whether the decrease in these proteins is dependent only on translation inhibition or also on protein destabilization, we conducted a Cycloheximide (CHX) chase assay, commonly used to measure the steady-state protein stability. After 16 h of treatment with 5 nM of Btz, the dose and time in which METTL3 protein expression levels did not decrease, while WTAP started to decrease significantly, we exposed cells to CHX for 1 and 3 h. Intriguingly, after CHX treatment WTAP protein decreased significantly already after 1 h, almost disappearing after 3 h, faster upon Btz treatment, while METTL3 did not. These data suggest that WTAP has a very short half-life, and the treatment with Btz 5 nM strongly destabilizes this protein (Fig. 1E). Thus, protein degradation enhances WTAP diminution, not excluding the contribution of inhibition of protein synthesis.

### Btz treatment induces the integrated stress response (ISR)

**Fig. 2:**
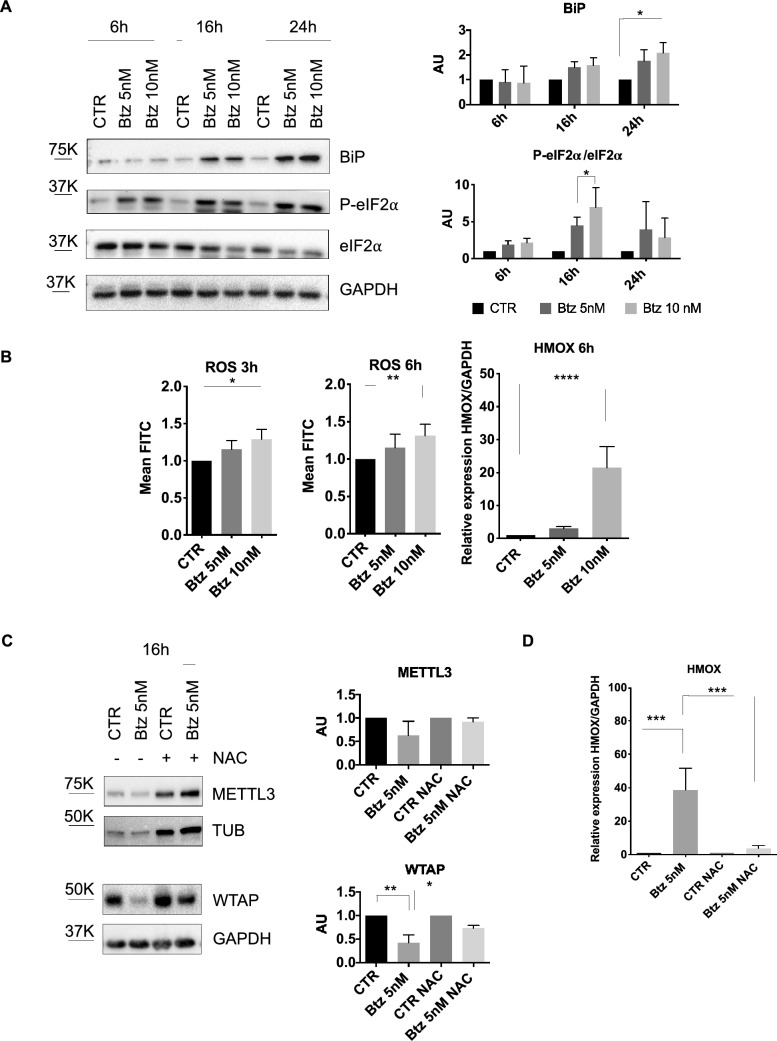
**A** Representative western blot analysis of BiP, P-eIF2α and eIF2α in MOLM-13 cells treated with 5 nM and 10 nM of Btz for 6, 16 and 24 h, with relative quantification (n = 3). **B** Cytofluorimetric detection of ROS after 3 h and 6 h of Btz treatment at the final concentration of 5 nM and 10 nM by using oxidized DCF-DA (on the left panel), (n = 3). On the right RT-qPCR analysis of HMOX-1 after 6 h of 5 nM and 10 nM of Btz (n = 4). **C** Representative western blot of METTL3 and WTAP after 16 h with 5 nM of Btz treatment, in presence or not of NAC and relative quantification (n = 3). **D** RT-qPCR analysis of HMOX-1 in the same previous described experimental condition (n = 3).*P ≤ 0.05; **P ≤ 0.005; ***P ≤ 0.0005; statistical analysis was performed by Two-Way ANOVA

As previously mentioned, several cellular stresses can phosphorylate eIF2α, impairing global translation efficiency and activating the Integrated Stress Response (ISR). Given the significant decrease in global protein synthesis, we investigated whether Btz treatment induced ISR by assessing eIF2α phosphorylation levels. Figure 2A illustrates that Btz treatment increased the phosphorylated form of eIF2α, particularly after 16 h , indicating IRS induction by the Btz doses used. Moreover, we observed an increase of BiP protein, one of the main ER chaperones, upon Btz treatment, indicating the presence of ER stress likely generated by the misfolded protein accumulation due to proteasome inhibition. As described in the introduction, under ER stress conditions, the oxidative folding of proteins, i.e. the formation of disulfide bonds, can occur in a dysregulated manner, leading to ROS accumulation and oxidative stress. Therefore, we investigated whether treatment with Btz could induce oxidative stress. We measured the amount of ROS by cytofluorimetric analysis 3 h and 6 h after Btz treatment. As shown in Fig. 2B, we observed an accumulation of ROS after 3 h and 6 h with the highest concentration of Btz, 10 nM. Furthermore, we found increased expression of heme oxygenase-1 (HMOX-1) mRNA, which is activated during the antioxidant response. To evaluate the contribution of oxidative stress to the Btz-induced down-regulation of METTL3 and WTAP, we treated cells with N-acetylcysteine (NAC), a commonly used pharmacological antioxidant and cytoprotectant. We administrated NAC to MOLM-13 cells, one day in advance of Btz treatment. After 16 h of Btz treatment, samples treated with NAC recovered protein expression levels, particularly significant for WTAP, suggesting that Btz-mediated protein decrease may be due to oxidative stress (Fig. 2C). Moreover, in the same experimental conditions, we performed RT-qPCR analysis of HMOX-1, which showed expression levels similar to the control samples as reported in Fig. 2D.

### Btz-resistant clones are able to control oxidative stress

**Fig. 3:**
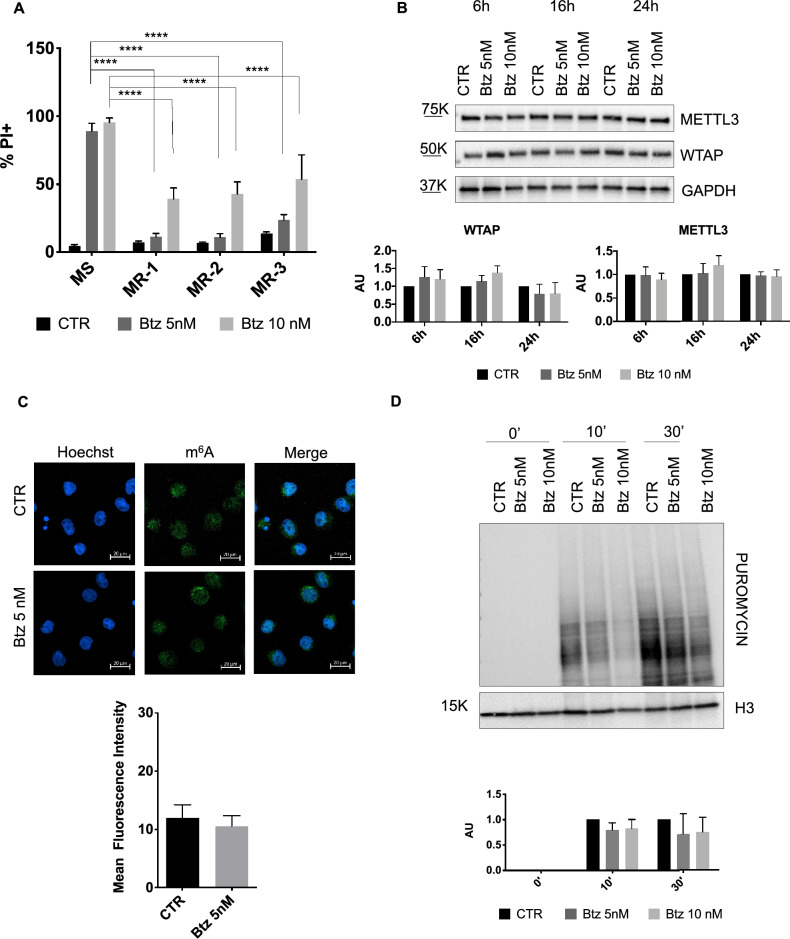
**A** Cytofluorimetric analysis of cell death detected by propidium iodide (PI) exclusion assay in MS and MR cells treated with Btz 5 nM and 10 nM for 72 h (n = 4). **B** Representative western blot of METTL3 and WTAP with relative quantification in MR-1 cells, treated with 5 nM and 10 nM of Btz for 6, 16 and 24 h (n = 4). **C** MR-1 cells were treated with 5 nM of Btz for 16 h, labeled with Hoechst (cell nuclei) and with anti-m^6^Ab labeled with Alexa Fluor 488 (m^6^A modification). On the bottom, is reported the graph of mean fluorescence quantification. Scale bars, 20 μm. (n = 2). **D** Detection by western blot analysis of puromycin incorporation after 0’, 10’ and 30’ in MR-1 cells control and treated with Btz 5 nM for 16 h. On the bottom is presented the quantification relative to the control for each time point (n = 3). ****P ≤ 0.00005; statistical analysis was performed by Two-Way ANOVA

**Fig. 4:**
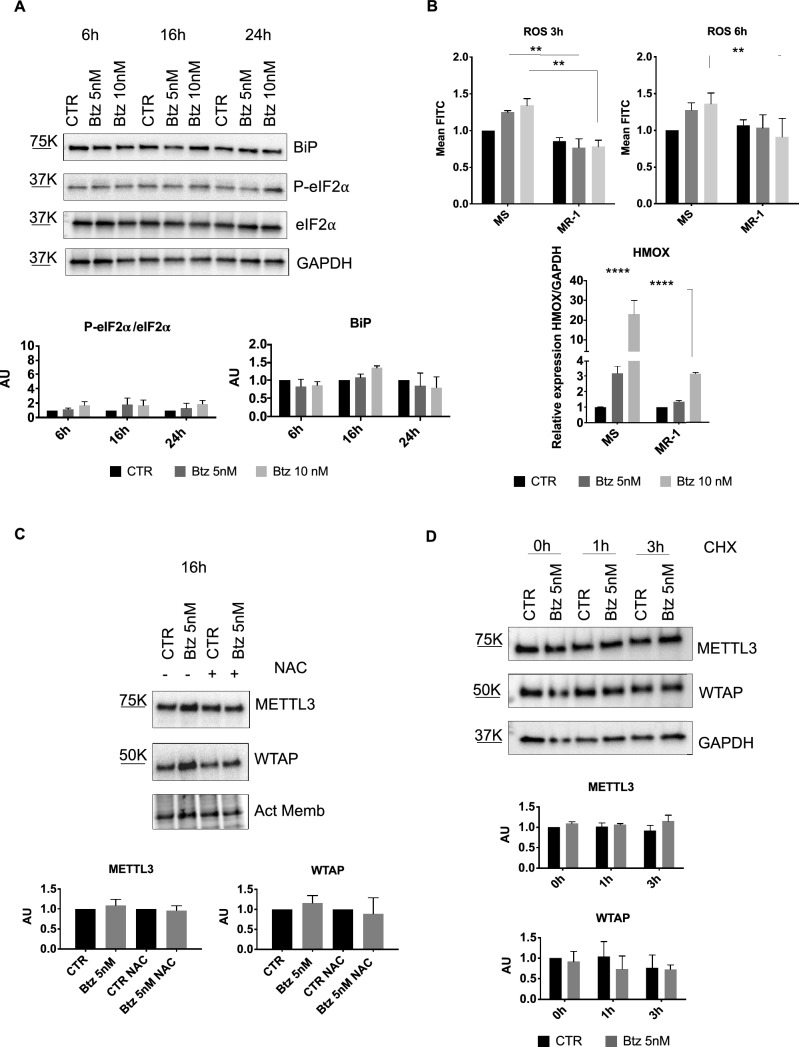
**A** Representative western blot analysis of BiP, P-eIF2α and eIF2α in MR-1 cells treated with 5 nM and 10 nM of Btz for 6, 16 and 24 h (n = 3). **B** ROS detection after 3 h and 6 h of Btz treatment at the final concentration of 5 nM and 10 nM in MOLM-13 and MR-1 cells, by cytofluorimetry. On the bottom, RT-qPCR analysis of HMOX-1 in the same samples treated with Btz for 6 h, as described above (n = 3). **C** Representative western blot of METTL3 and WTAP after 16 h with 5 nM of Btz treatment, in presence or not of NAC and relative quantification (n = 3). **D** Representative western blot analysis of MR-1 cells treated with 5 nM of Btz for 16 h and subsequently with CHX for additional 0 h (CHX -), 1 h and 3 h; on the bottom, relative quantification of METTL3 and WTAP protein levels (n = 2). *P ≤ 0.05; **P ≤ 0.005; ***P ≤ 0.0005; statistical analysis was performed by Two-Way ANOVA

Next, we generated MOLM-13 Btz-resistant clones for functional studies. We exposed the MOLM-13 cell line to doses of Btz increasing every 4 weeks, changing the medium every 4 days. After three months of treatment, we seeded the cells in 96 wells in serial dilution, and we isolated the single resistant clones (subclones). In total, we isolated 3 different MOLM-13 Btz-resistant clones (MR), on which we performed functional assays. We first confirmed the resistance of our clones to Btz by treating them with 5 nM and 10 nM concentrations for longer than 72 h and similarly we treated the parental MOLM-13 cell line. After 72 h of treatment, we performed a cytofluorimetric analysis of cell death upon propidium iodide uptake. As shown in Fig. 3A, after 72 h of treatment with 5 nM of Btz, there was no cell death in the MR cell lines. We observed 50% cell death with 10 nM Btz, which still indicates resistance when comparing these results with those obtained with the parental MOLM-13 cell line, which resulted in 100% cell death upon treatment with both concentrations. Among the MR clones, MOLM-13 Btz resistant 1 (MR-1) showed the best resistance to the treatment, so we selected this one for further functional studies. As Btz treatment in MOLM-13 cells induced down-regulation of METTL3 and WTAP, we investigated their expression in the MR-1 cells under the same experimental conditions, Btz 5 nM and 10 nM for 6, 16 and 24 h. Additionally, we also assessed YTHDC1 and FTO protein expression levels. As represented in Fig. 3B and in Supplementary Figure S2A, the MR-1 cells no longer showed downregulation of the m^6^A regulators upon Btz treatment compared to control samples, nor did we detect any significant deregulation of METTL3, WTAP, YTHDC1 and FTO mRNA levels (Supplementary Figure S2B). As expected, we observed no changes in m^6^A modification levels in MR-1 cells treated with Btz 5 nM for 16 h (Fig. 3C). Moreover, unlike the parental MOLM-13 cell line, there was no overall decrease in global translation in MR-1 cells, as illustrated in Fig. 3D. Considering the induction of stress resulting in ISR activation observed previously, we investigated the Btz-induced stress response in the MR-1 clone. We analyzed phosphorylation of eIF2α protein, which showed a slight, statistically non-significant, increase, while the BiP protein expression level remained constant in a dose and time-dependent manner (Fig. 4A). In order to evaluate Btz-induced oxidative stress in Btz-resistant cells, we analyzed the amount of ROS in MR-1 cells treated with 5 nM and 10 nM Btz for 3 and 6 h. As shown in Fig. 4B, Btz treatment did not increase ROS levels in the MR-1 cells in all the experimental conditions, unlike previously observed in the parental MOLM-13 cells. Accordingly, the MR-1 cells activated the antioxidant response after 6 h of stress induced by the highest concentration of Btz, as evidenced by increased levels of HMOX-1 mRNA, albeit at lower levels than that induced in the MOLM-13 cell line (Fig. 4B). As expected, no changes were observed upon administration of NAC to MR-1 cells treated with Btz (Fig. 4C).

Interestingly, we treated MR-1 cells with 5 nM of Btz for 16 h with and without CHX and we did not observe a significant reduction in WTAP protein levels. This suggests that in MR-1 cells, WTAP indeed has a longer half-life compared to parental MOLM-13 cells (Fig. 4D) and this may play a crucial role in the cellular stress response. Altogether, these data suggest that Btz treatment induced a certain degree of stress, but Btz-resistant cells are nonetheless capable of managing it.

### Btz treatment induces the methylation and expression of a subset of m^6^A-modified circRNAs

**Fig. 5:**
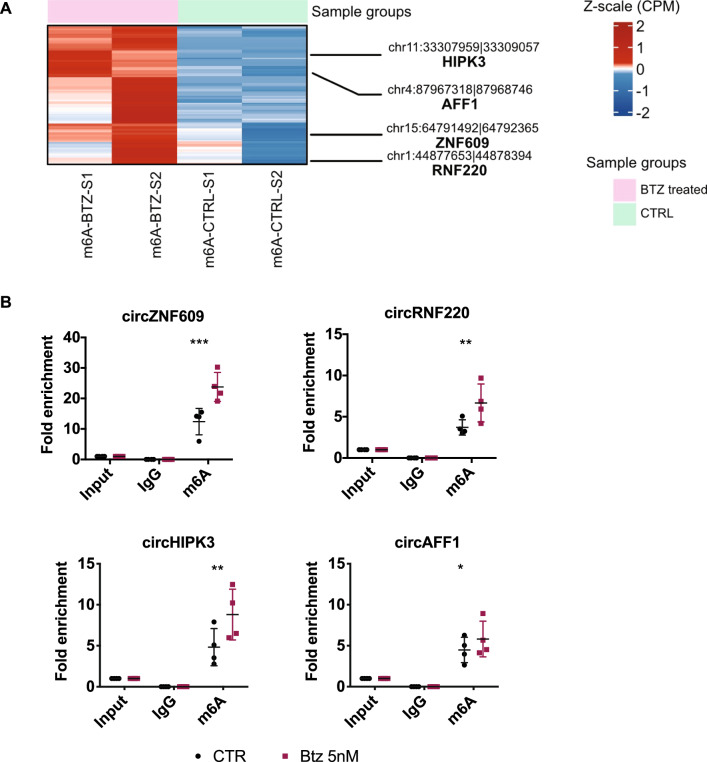
**A** Unsupervised clustering heatmap showing the z-score cpm enrichment of circRNAs identified by m^6^A-IP-RNAseq in MOLM-13 cells treated with Bortezomib vs control untreated cells. The circRNAs experimentally validated were highlighted on the left side of the heatmap. Color legend: pink = Bortezomib treated samples; light green = control samples. **B** RT-qPCR analysis of circZNF609, circRNF220, circHIPK3 and circAFF1 in control and Btz-treated samples after m^6^A-IP (n = 4). *P ≤ 0.05; **P ≤ 0.005; ***P ≤ 0.0005; statistical analysis was performed by Two-Way ANOVA

**Fig. 6:**
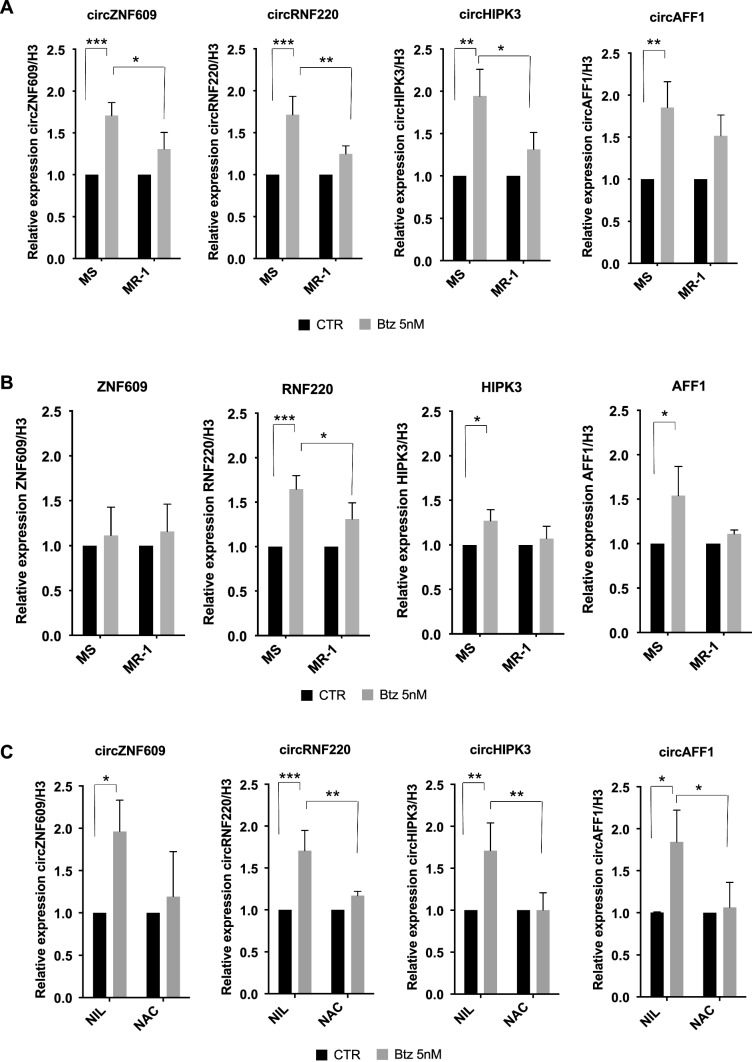
**A** RT-qPCR analysis of circZNF609, circRNF220, circAFF1 and circHIPK3 in control and Btz-treated samples in MS and MR-1 cells (n = 3). **B** RT-qPCR analysis ZNF609, RNF220, AFF1 and HIPK3 in control and Btz-treated samples in MS and MR-1 cells (n = 3). **C** RT-qPCR analysis of circZNF609, circRNF220, circAFF1, circHIPK3 and circSATB1 in control and Btz-treated samples in MOLM-13 treated with water (control) or NAC (n = 3). Each untreated cell line is set at 1.0. *P ≤ 0.05; **P ≤ 0.005; ***P ≤ 0.0005; statistical analysis was performed by Two-Way ANOVA

As mentioned, among our aims there was the identification of m^6^A-modified circRNAs differentially expressed upon proteotoxic stress. Once we assessed that Btz alters m^6^A levels, we performed m^6^A-Immunoprecipitation (m^6^A-IP) on RNA extracted from MOLM-13 cells treated with 5 nM of Btz for 16 h. In detail, total RNA was isolated, treated with RNaseR to remove all the linear RNA and most of the ribosomal RNA before proceeding with m^6^A-IP. Input and m^6^A-immunoprecipitated RNAs from CTR- and Btz-treated cells were then analyzed by RNA-seq and circRNAs expression values are available on GEO dataset (GSE262829). To select m^6^A circRNA deregulated between CTR and Btz-treated MOLM-13 cells, we employed unsupervised clustering analysis on normalized RNA-seq enrichment of detected m^6^A circRNAs (Fig. 5A and Supplementary Table 1). Although the global m^6^A RNA level is decreased, we identified a signature of circRNAs that showed an increase in m^6^A methylation after Btz treatment. Hierarchical clustering enabled to identify a set of 126 m^6^A circRNA that exhibited higher expression in the m^6^A-IP Btz-treated samples compared to the m^6^A-IP untreated samples. RT-qPCR validation was executed on four circRNA selected from this set using divergent primers. As shown in Fig. 5B, circZNF609, circHIPK3, circRNF220 and circAFF1 exhibited hyper-methylation following Btz treatment, compared to the control samples. We then investigated how their expression, along with their linear counterparts, changed in MOLM-13 parental cells and MR-1 cells upon Btz treatment. We treated MOLM-13 and MR-1 cells with 5 nM Btz for 16 h and assessed the expression of the selected circRNAs and their linear transcripts using RT-qPCR. As shown in Fig. 6A,B, we observed a significant upregulation of the four selected and hypermethylated m^6^A-modified circRNAs following Btz treatment. Interestingly, the upregulation of circZNF609, circRNF220 and circHIPK3 in response to Btz treatment was attenuated in MR-1 cells under the same experimental conditions, as was the case for circAFF1, although not to a statistically significant extent. These data suggest that Btz treatment increased both methylation levels and expression of the selected circRNAs. Regarding their linear counterparts, we observed a significant but less pronounced increase in RNF220, AFF1, and HIPK3 mRNA expression, while ZNF609 showed no variation. To investigate the impact of oxidative stress on circRNA expression in response to Btz, we treated MOLM-13 cells with Btz 5 nM with or without NAC. As shown in Fig. 6C, circRNAs expression increased in Btz-treated samples, but not in those pre-treated with NAC. This suggests that the selected circRNAs, hyper-methylated and overexpressed upon Btz treatment, are dependent on oxidative stress and may play a role in modulating Btz-induced stress.

### circHIPK3 alleviates oxidative and ER stress

**Fig. 7:**
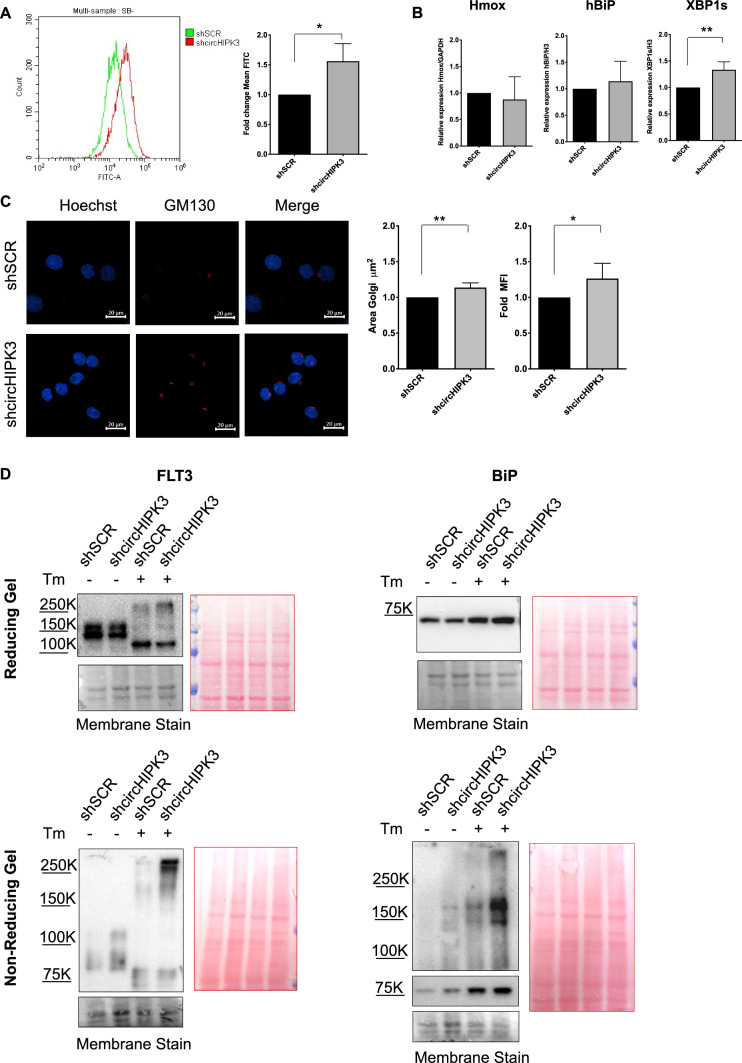
**A** shSCR and shcircHIPK3 cells were plated and after 24 h cytofluorimetric detection of ROS was performed by using oxidized DCF-DA (n = 3). **B** RT-qPCR analysis of HMOX-1, hBiP and XBP1s in shSCR and shcircHIPK3 after 24 h of plating (n = 3). **C** shSCR and shcircHIPK3 cells were plated and, after 24 h, were labeled with Hoechst (cell nuclei) and with anti-GM130 labeled with Alexa Fluor 555. On the right, is reported the graph of Golgi Area (µm^2^) and mean fluorescence quantification. Scale bars, 20 μm. (n = 4). **D** Representative western blot of FLT3 and BiP in Reducing (R, upper panel) and Non-Reducing (NR, lower panel) conditions, of shSCR and shcircHIPK3 cells after 16 h of 300 ng/mL Tm treatment (n = 3). *P ≤ 0.05; **P ≤ 0.005; statistical analysis was performed by Two-Way ANOVA

Among the four validated circRNAs, circHIPK3 certainly has captured our interest since its antioxidant role is well-known in literature [55]. Therefore, we silenced circHIPK3 using shRNA and we analyzed, in a time course, whether our circRNA was silenced without influencing the expression of the linear counterpart. Supplementary Fig. 3A demonstrates the stable silencing of circHIPK3. Silencing did not affect cell death and proliferation, as also indicated by the cell cycle analysis performed 72 h post-seeding (Supplementary Fig. 3B). Subsequently, we investigated the response to oxidative and ER stress. We observed a significant increase in reactive oxygen species 24 h after shcircHIPK3 cells seeding, suggesting a possible role of circHIPK3 in alleviating oxidative stress also in acute myeloid leukemia (Fig. 7A). However, as shown in Fig. 7B, steady-state ROS levels in the circHIPK3-silenced cells were not sufficient to activate HMOX-1, suggesting a mild level of stress. Regarding basal ER stress, BiP expression levels were not increased, but we observed an increase in the spliced form of X-box binding protein 1 (XBP1s). Under ER stress conditions, the UPR sensor IRE1α splices XBP1 mRNA generating the spliced form XBP1s. XBP1s is a transcription factor, which migrates to the nucleus and activates UPR target genes encoding ER chaperones, folding enzymes, and ERAD to attenuate ER stress [56]. Upon ER stress, the UPR triggers a series of events that influence the structure and function of cellular organelles, including the Golgi apparatus. Consistent with this, we found increased dimension of the Golgi apparatus in the circHIPK3-silenced cells, analyzed by immunofluorescence upon staining for the Golgi marker GM130 (Fig. 7C). Alterations in XBP1s expression and Golgi size suggest perturbation of the secretory pathway. Thus, we evaluated the expression levels of a secretory protein in circHIPK3-silenced cells compared to control. Based on literature, retinoic acid (RA) administration is not able to induce a complete differentiation (as in Acute Promyelocytic Leukemia, APL) in AML cells, although it causes signs of differentiation and activates the secretory pathway [30]. Thus, we measured by flow cytometry the expression of a plasma membrane protein, the myeloid-monocytic lineage differentiation maker CD11b, upon treatment of shSCR and shcircHIPK3 MOLM-13 cells with 10^–6^ RA for 72 h. CD11b levels increased in MOLM-13, shSCR and shcircHIPK3 RA-treated samples compared to the respective control samples (CTR). Interestingly, shcircHIPK3 cells displayed a significantly lower level of CD11b compared to MOLM-13 and shSCR cells (Supplementary Fig. 3D), after RA treatment, indicating alterations in the secretory pathway in the absence of circHIPK3.

To further investigate this aspect, we decided to treat shSCR and shcircHIPK3 cells with the N-glycosylation inhibitor Tunicamycin (Tm), a well-known ER inducer. We tested three different concentrations of Tm for 72 h and observed increased sensitivity of circHIPK3-silenced cells to the treatment compared to control cells (shSCR) (Supplementary Fig. 4A). We chose the intermediate concentration of Tm (300 ng/mL) to observe the potential accumulation of misfolded proteins. As shown in Fig. 7D, circHIPK3-silenced cells treated for 16 h with Tm (300 ng/mL) exhibited a significant increase in total BiP levels (Fig. 7D reducing gel), and a considerable accumulation of FLT3 and BiP high molecular weight aggregates in comparison to control samples treated with Tm (Fig. 7D non-reducing gel and Supplementary Fig. 4B). Although not significant, a mild increase in BiP aggregates in the non-reducing western blot conditions was observed in the untreated shcircHIPK3 sample, confirming a slight increase in ER stress and accumulation of unfolded proteins already at the steady state. As mentioned above FLT3-ITD is a misfolded protein retained in the ER and BiP is the main ER chaperone that forms complexes with misfolded proteins that accumulate in the ER; thus, the presence of high molecular weight aggregates of these proteins indicates an impairment of ER folding capacity. These data confirm the protective role of circHIPK3 in alleviating oxidative and ER stress in AML cells.

## Discussion

AML is a blood disease characterized by the arrest of differentiation of hematopoietic progenitors, leading to the uncontrolled proliferation of leukemic blasts. The primary treatment, chemotherapy, is often followed by hematopoietic stem cell transplantation, but this may not be optimal, and in many cases, it is not possible, especially for the elderly [57]. Most of these malignancies are characterized by chromosomal abnormalities and mutations, and some of these, like FLT-ITD, cause the accumulation of misfolded protein and ER stress. Tumor cells, in response to mutations and oxidative stress, activate pro-survival mechanisms such as the UPR and oxidative stress response [58–60].

Several research groups have highlighted these aspects and how this balance can be disturbed using cellular stress inducers to cause the death of cancer cells, for instance by blocking protein glycosylation rather than the machinery responsible for protein degradation. In this context, our laboratory developed a combination of drugs, including Tm, Arsenic Trioxide (ATO) and RA at physiological doses, to induce ER stress and oxidative stress [61]. Another stress inducer is the proteasome inhibitor Bortezomib (Btz), the first one approved by the Food and Drug Administration, for treating mantle cell lymphoma and multiple myeloma, and we demonstrated that used in combination with RA and ATO efficiently causes FLT3-ITD + AML cell death [30]. Despite generating strong cellular stress, Btz is not used as a single agent for treating AML and requires further investigation.

AML is characterized by elevated expression of proteins responsible for N^6^-methyladenosine (m^6^A) modification. Recent studies have shed light on m^6^A’s role in the biogenesis and translation of a new class of RNAs, circRNAs [62–64].

Both m^6^A and circRNAs are involved in stress induction and response, but this field deserves elucidation. Based on what has been described so far, and as the role of Btz on m^6^A and circRNAs is unknown, we wondered whether modulation of m^6^A-modified circRNAs could contribute to the Btz-induced stress response.

To address this, we treated the FLT3-ITD + MOLM-13 cell line with Btz to induce acute stress, observing a slight downregulation of METTL3 and a much stronger downregulation of WTAP protein expression levels. The latter was one of the first m^6^A regulators to be discovered [65] and its deregulation and involvement in AML are well-established and demonstrated by numerous studies [66, 67]. Surprisingly, WTAP protein exhibited a short half-life, which could account for its faster decrease after Btz treatment compared to METTL3. A similar trend of protein expression levels was observed for YTHDC1 and FTO respectively, both of them involved in oxidative stress induction [68, 69].

The stress response is finely regulated and different concentrations and/or conditions can lead to different effects. The Btz concentrations we used in the AML cell line induced ISR activation, as evidenced by the phosphorylation of eIF2α and the resulting reduction in overall protein synthesis. Btz treatment also generated oxidative stress, which contributes to the lowered protein expression levels of WTAP, as evidenced by its recovery following NAC administration. NAC is one of the most used antioxidants to prevent oxidative stress effects, since it is a precursor for glutathione biosynthesis, reduces disulfide bonds, and acts as a scavenger of ROS [70].

As previously mentioned, different studies have highlighted the intricate and contradictory roles of oxidative stress on m^6^A modifications, alongside their reciprocal relationship [71]. Our study contributes to this understanding by revealing that Btz-induced proteotoxic and oxidative stress has a negative influence on overall m^6^A levels. Since m^6^A also regulates the biogenesis of circRNAs and their translation in some cases, it was crucial to investigate possible m^6^A-modified circRNAs that might play a role in the Btz-induced stress response.

Surprisingly, despite the general decrease in m^6^A RNA methylation, m^6^A-IP followed by circRNA-seq, showed that there was a group of 126 circRNAs that exhibited hyper-methylation upon Btz treatment, compared to control samples. We focused our attention on hypermethylated circRNAs in a hypomethylation context because they are those that, induced by Btz treatment, could play a role in the stress response. Additionally, among our hypermethylated circRNAs, some had already been described as having a role in the stress response or AML progression [72–74], which further motivated us to analyze this subset of circRNAs. In particular, we analyzed four hyper-methylated circRNAs, those that were most enriched in the m^6^A-immunoprecipitated samples: circZNF609, circRNF220, circHIPK3, circAFF1.

In AML treatment, relapses and resistance pose significant challenges, despite molecular targeted drugs like the Bcl-2 inhibitor Venetoclax and FLT3 inhibitors being approved. Ongoing studies explore combining Venetoclax with FLT3 inhibitors to tackle resistance mechanisms, particularly prevalent FLT3 mutations, offering potential solutions for relapse and treatment failure [75–77]. Despite the recently developed combined treatments, it is important to identify other molecular targets that may be useful in overcoming drug resistance. For this purpose, we established Bortezomib (Btz)-resistant cell lines, exposing cells to increasing Btz doses. The resulting MR cells displayed resistance to cell death, maintained normal m^6^A protein expression levels post-Btz treatment, and exhibited reduced upregulation of circRNAs observed in the parental MOLM-13 cell line treated with Btz. Notably, pre-treatment with NAC in MOLM-13 cells blocked the Btz-induced increase in these circRNAs, suggesting their potential involvement in the oxidative stress response. Nevertheless, further investigations are still needed to elucidate the whole picture of this complex OxS-m^6^A-circRNAs axis induced by Btz in AML progression and resistance.

We focused our attention on studying circHIPK3 role in AML. In recent years, several research groups have highlighted its role as a diagnostic and prognostic biomarker in various tumors as well as its role in tumor progression and in chemotherapeutics sensitization [78]. In particular, various studies have demonstrated the ability of circHIPK3 to alleviate oxidative stress. Wang Y. et al., observed its antioxidant function and therefore a protective effect of circHIPK3 against oxidative stress-induced damage in cardiac microvascular endothelial cells (CMVECs) [72]. Furthermore, Liang et al. demonstrated the protection of human osteoblasts from oxidative damage through the circHIPK3-miR-124 axis [79]. In this study, we demonstrate that circHIPK3 has an important role in oxidative and ER stress regulation also in AML cells. Indeed, our findings show increased ROS levels and greater misfolded protein accumulation when we induced ER stress with Tm in circHIPK3-silenced cells compared to control cells.

In conclusion, our work demonstrates that proteotoxic and oxidative stress induced by proteasome inhibition affects the m^6^A modification pathways, the methylation and expression of specific circRNAs. These could be important molecular biomarkers for proteostasis-related oxidative stress and relevant targets to understand AML resistance to treatments.

### Supplementary Information

Below is the link to the electronic supplementary material.

Supplementary file1 (PDF 2263 KB)

Supplementary file2 (PDF 1426 KB)

Supplementary file3 (PDF 663 KB)

Supplementary file4 (PDF 160 KB)

Supplementary file5 (PDF 6939 KB)

## Data Availability

All data supporting the findings of this study are available within the paper and its Supplementary Information. circRNA expression values are deposited in GEO database.

## Abbreviations

m^6^A: N^6^-methyladenosine
circRNAs: Circular RNAs
AML: Acute myeloid leukemia
Btz: Bortezomib
UPR: Unfolded protein response
ER: Endoplasmic reticulum
ERAD: ER stress-associated degradation
ROS: Reactive oxygen species
CHX: Cycloheximide
ISR: Integrated stress response
HMOX-1: Heme oxygenase-1
NAC: N-acetylcysteine
MR: MOLM-13 Btz resistant
XBP1s: X-box binding protein 1
RA: Retinoic acid
Tm: Tunicamycin
ATO: Arsenic trioxide

## Code availability

GSE262829.
