# Supplementary material for: WTAP and m6A-modified circRNAs modulation during stress response in acute myeloid leukemia progenitor cells
Source: Cell Mol Life Sci. 2024 Jun 23;81(1):276. doi: 10.1007/s00018-024-05299-9 (PMC11335200; doi:10.1007/s00018-024-05299-9)
Supplement: Supplementary file 1 — Supplementary file1 (PDF 2263 KB) [file 18_2024_5299_MOESM1_ESM.pdf]

Figure S1

A

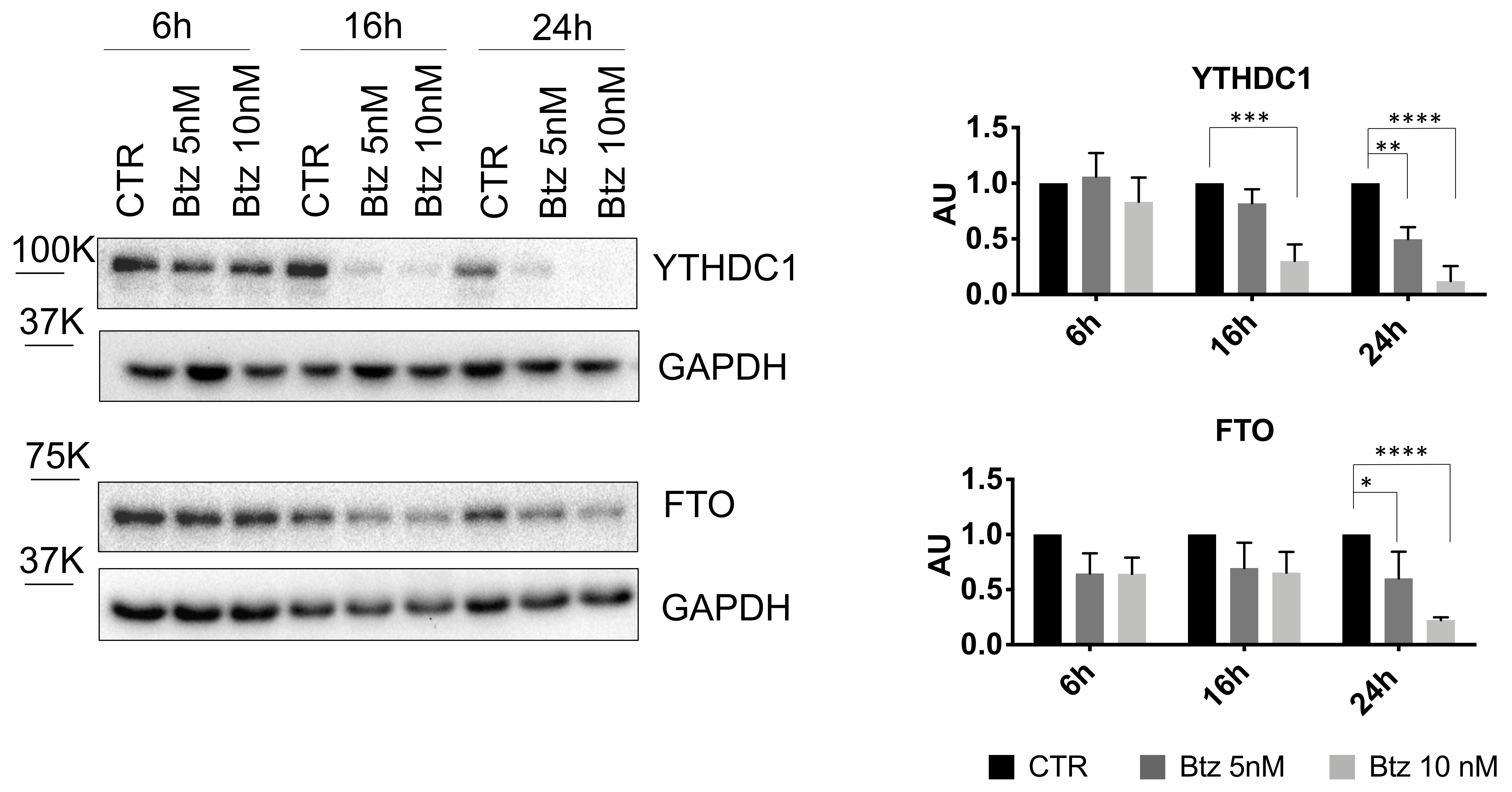

B

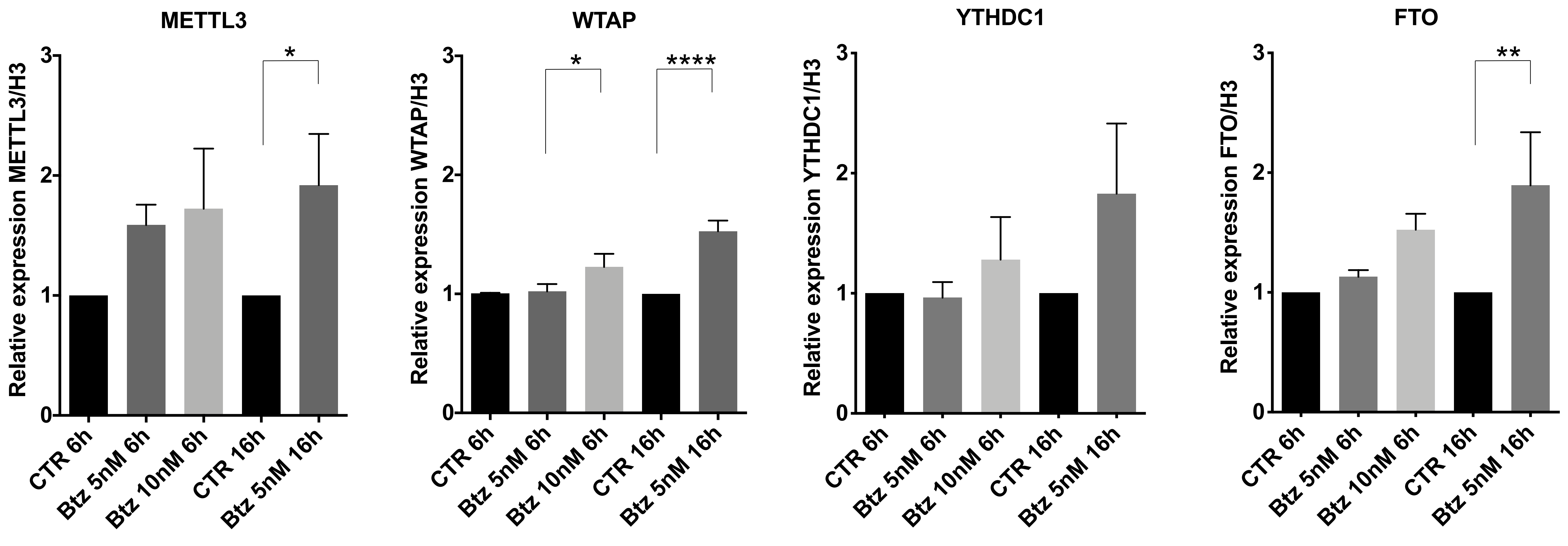

Supplementary Figure 1A. Representative western blot of YTHDC1 and FTO and relative quantification after 6h,16h and 24h of treatment with Btz 5nM and 10nM (n=3). **B.** RT-qPCR analysis of METTL3, WTAP, YTHDC1 and FTO mRNA expression after 6h of treatment with 5 nM and 10 nM of Btz, and after 16h of treatment with 5 nM of Btz (n=3). *P≤0.05; **P≤0.005; ***P≤0.0005; ****P≤0.00005; statistical analysis was performed by Two-Way ANOVA.
