# Supplementary material for: WTAP and m6A-modified circRNAs modulation during stress response in acute myeloid leukemia progenitor cells
Source: Cell Mol Life Sci. 2024 Jun 23;81(1):276. doi: 10.1007/s00018-024-05299-9 (PMC11335200; doi:10.1007/s00018-024-05299-9)
Supplement: Supplementary file 3 — Supplementary file3 (PDF 663 KB) [file 18_2024_5299_MOESM3_ESM.pdf]

Figure S3

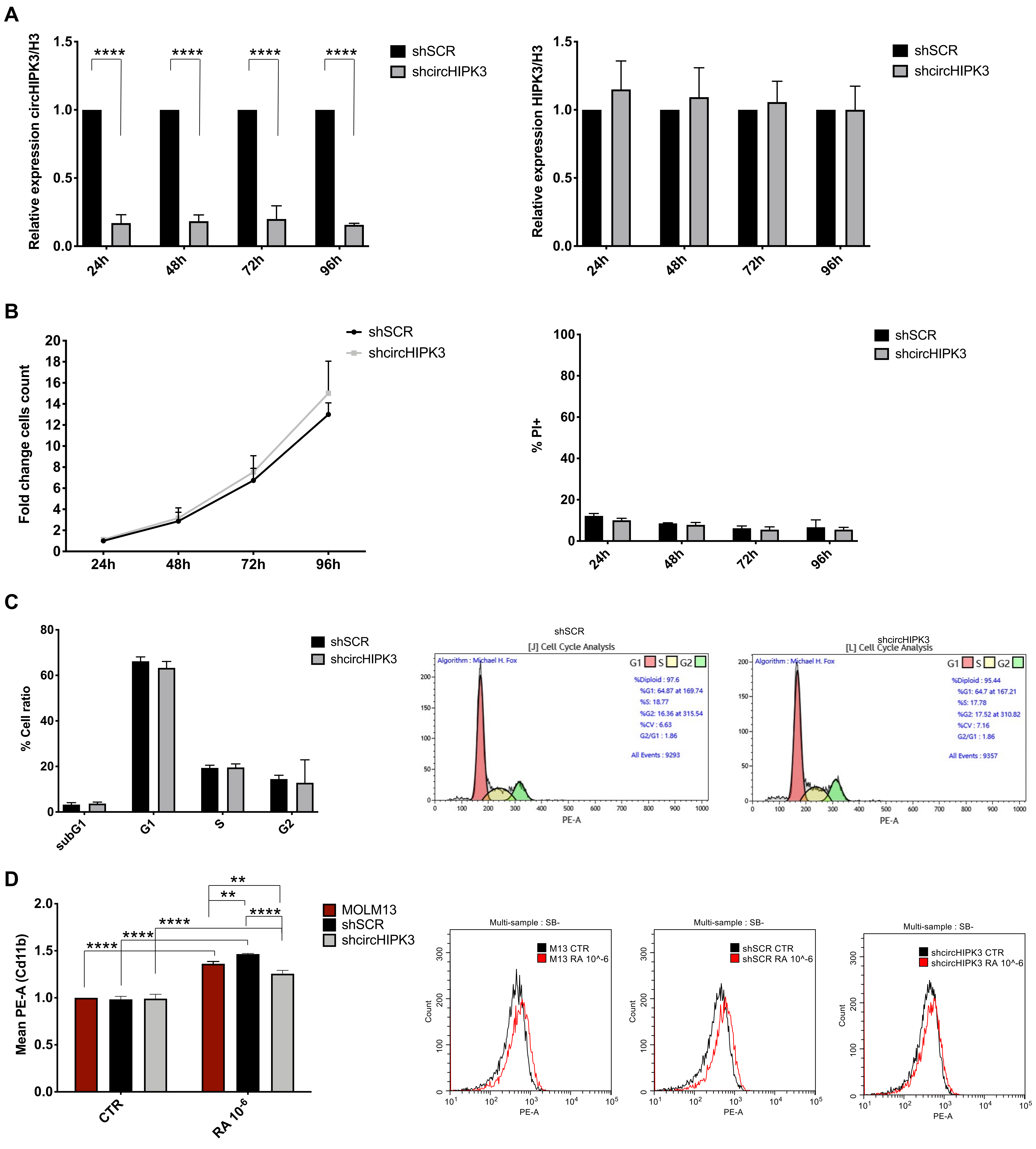

Supplementary Figure 3 A. RT-qPCR analysis of circHIPK3 and HIPK3 24h, 48h, 72h and 96h after plating (n=3). **B.** Cell count foldchange and cell death analysis of shSCR and shcircHIPK3 cells in the same experimental conditions described above (n=3). **C.** Cytofluorimetric analysis of cell cycle of shSCR and shcircHIPK3 72h after plating (n=3). **D.** Expression of Cd11b expression measured as Mean PE-A by FACS in MOLM13, shSCR and shcircHIPK3 CTR or treated with 10^{-6} RA. (n=3) **P≤0.005; ****P≤0.00005; statistical analysis was performed by Two-Way ANOVA.
