# Supplementary material for: WTAP and m6A-modified circRNAs modulation during stress response in acute myeloid leukemia progenitor cells
Source: Cell Mol Life Sci. 2024 Jun 23;81(1):276. doi: 10.1007/s00018-024-05299-9 (PMC11335200; doi:10.1007/s00018-024-05299-9)
Supplement: Supplementary file 4 — Supplementary file4 (PDF 160 KB) [file 18_2024_5299_MOESM4_ESM.pdf]

Figure S4

A

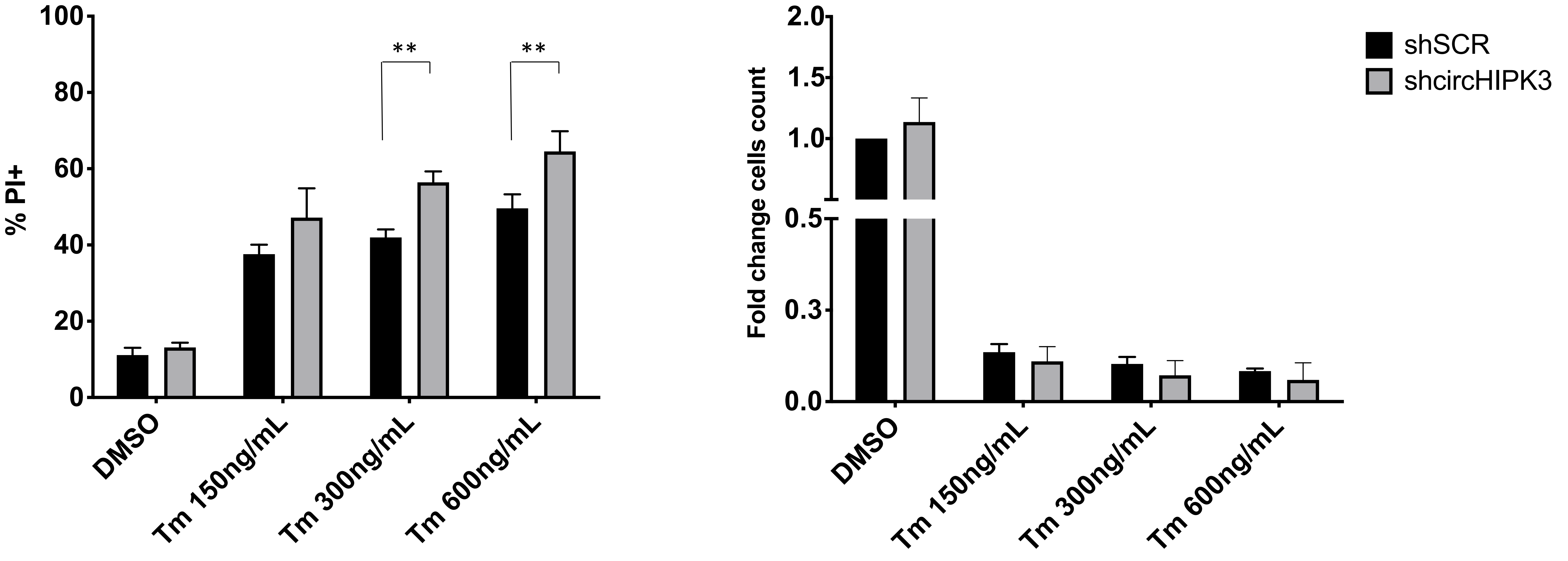

B

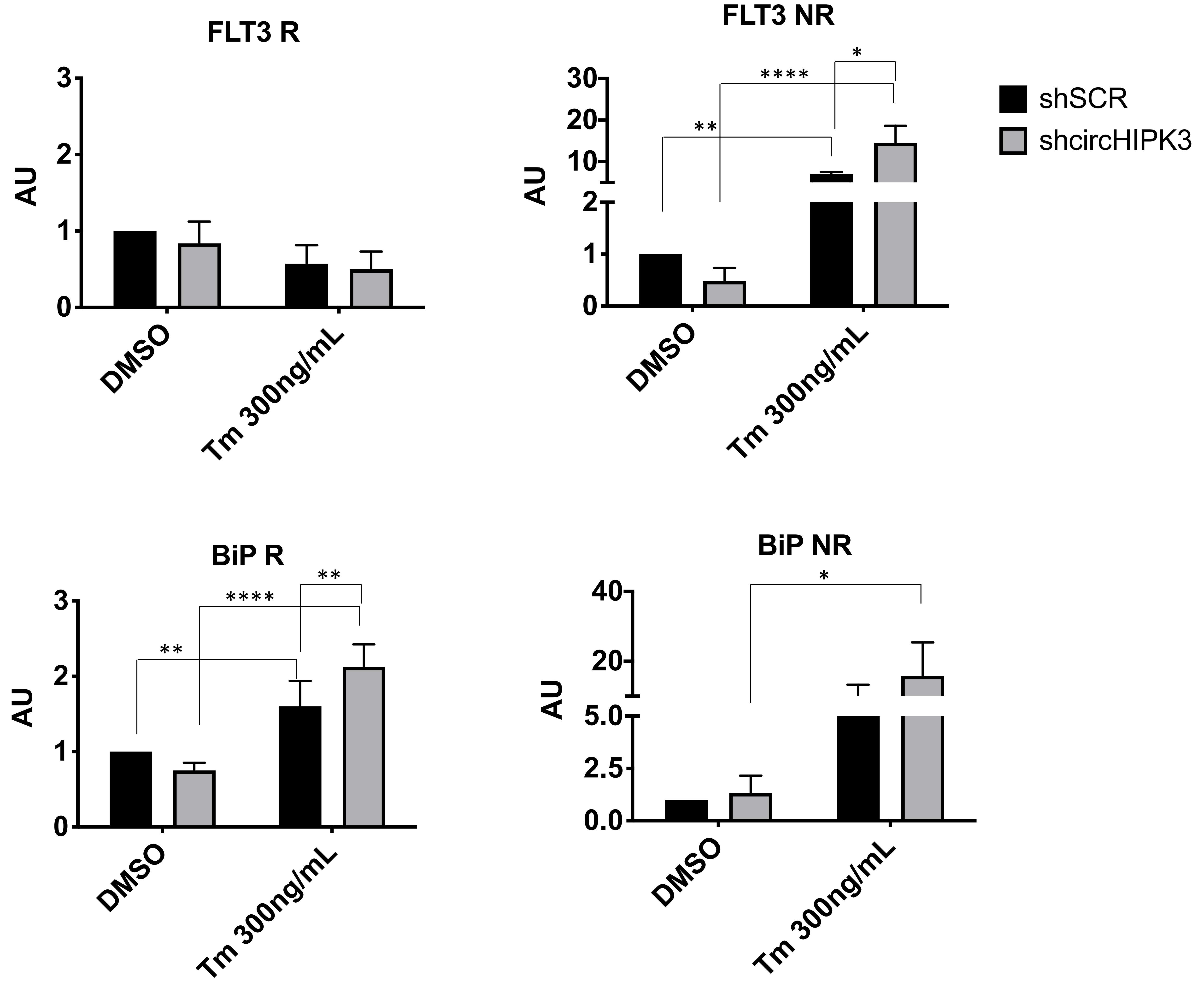

Supplementary Figure 4 A. Cytofluorimetric analysis of cell death and cell count after 72h of 150ng/mL, 300ng/mL and 600ng/mL Tm treatment (n=3). **B.** Relative quantification of gels represented in Figure 7D, R=Reducing Gel, NR=Non-Reducing Gel (n=4). *P≤0.05; **P≤0.005; ****P≤0.00005; statistical analysis was performed by Two-Way ANOVA.
