# Supplementary material for: WTAP and m6A-modified circRNAs modulation during stress response in acute myeloid leukemia progenitor cells
Source: Cell Mol Life Sci. 2024 Jun 23;81(1):276. doi: 10.1007/s00018-024-05299-9 (PMC11335200; doi:10.1007/s00018-024-05299-9)
Supplement: Supplementary file 5 — Supplementary file5 (PDF 6939 KB) [file 18_2024_5299_MOESM5_ESM.pdf]

circRNA_coordinates	m6A-CTRL-S1	m6A-CTRL-S2	m6A-BTZ-S1	m6A-BTZ-S2	cluster_group
chr10:102683732|102685776	1.0082351	1.8826621	3.0190760	1.7005715	2
chr10:102777856|102778974	0.5994912	0.0000000	0.0000000	0.0000000	6
chr10:103552596|103567658	0.8174880	0.1711511	0.0000000	0.0000000	6
chr10:103552596|103570071	0.4632432	0.5990289	0.9289464	0.2616264	2
chr10:103558599|103567658	0.4904928	0.0000000	0.0000000	0.0000000	6
chr10:103916776|103917971	0.6539904	0.0855756	0.5160298	0.2616264	1
chr10:105177549|105198565	0.0000000	0.0000000	0.4644732	0.0000000	2
chr10:105197772|105198565	0.1089984	0.4278778	0.6967098	1.4389451	4
chr10:105199503|105200607	0.8174880	0.2567267	0.0000000	0.2616264	6
chr10:111860383|111860606	0.0000000	0.0000000	0.0000000	0.6540660	5
chr10:112356156|112358048	0.4904928	0.0855756	0.2322366	0.3924396	1
chr10:112635724|112642855	0.0000000	0.0000000	0.4644732	0.0000000	2
chr10:115636280|115644139	0.0000000	0.7701800	0.0000000	0.0000000	7
chr10:116719485|116734144	0.0000000	0.0000000	0.0000000	0.5232528	5
chr10:12009340|12056183	0.1907472	0.0000000	0.0000000	0.2616264	5
chr10:12039671|12056183	0.2724960	0.7701800	0.0000000	0.9156923	5
chr10:120445495|120460920	0.0000000	0.0000000	1.8578929	0.0000000	2
chr10:120488807|120489922	0.4724535	0.0000000	3.5506656	0.4290673	2
chr10:120797750|120797951	0.3542448	0.0855756	1.1611831	0.6540660	2
chr10:120801506|120803654	1.8802223	0.0000000	0.0000000	0.0000000	6
chr10:120809313|120810833	0.0000000	0.0000000	0.9805030	0.0000000	2
chr10:120867460|120892121	0.0000000	0.1711511	0.4644732	0.0000000	2
chr10:12123471|12136270	0.1089984	0.0000000	0.6967098	0.0000000	2
chr10:12123471|12162266	0.2997456	0.0000000	0.6967098	0.1245342	1
chr10:126370176|126370948	2.8612078	1.4547844	0.0000000	0.3924396	6

circRNA_coordinates	m6A-CTRL-S1	m6A-CTRL-S2	m6A-BTZ-S1	m6A-BTZ-S2	cluster_group
chr10:126370176|126384781	0.5177424	0.5134533	1.8578929	1.1773187	2
chr10:126370176|126395456	1.1444831	0.2567267	0.0000000	0.1308132	6
chr10:126631026|126631876	7.9296332	2.6528421	3.9480224	4.4476485	1
chr10:126631026|126655350	0.3814944	0.0855756	0.2322366	0.0000000	6
chr10:13214376|13214765	0.3163678	0.0000000	0.2933148	0.0664531	1
chr10:134161488|134169390	0.0000000	0.0000000	0.0000000	0.5232528	5
chr10:134459372|134464009	0.3814944	0.0000000	0.0000000	0.1308132	6
chr10:134503896|134523960	0.0000000	0.0000000	0.0000000	0.6540660	5
chr10:14970015|14977563	0.0000000	0.0000000	0.4644732	0.0082412	2
chr10:15858834|15885271	0.2452464	0.2567267	0.0000000	0.1308132	7
chr10:15875629|15889942	0.3269952	0.1711511	0.3871384	0.0622671	1
chr10:16509131|16528616	0.1907472	0.0000000	0.0000000	0.5232528	5
chr10:16526336|16528616	0.0000000	0.5134533	0.0000000	0.3924396	5
chr10:22048099|22095028	0.0000000	0.0000000	0.4644732	0.0000000	2
chr10:22171211|22209892	0.4087440	0.0000000	0.0000000	0.0000000	6
chr10:225934|295028	0.0000000	0.5134533	0.0000000	0.0000000	7
chr10:26789748|26802589	0.3269952	0.0000000	0.0000000	0.2616264	6
chr10:27420788|27425314	0.0000000	0.0000000	0.4644732	0.0000000	2
chr10:27453993|27454468	1.1947041	0.0855756	0.2322366	0.2616264	6
chr10:27458873|27462188	0.5994912	0.0000000	0.0000000	0.0000000	6
chr10:3159039|3160944	0.6812400	0.2567267	0.0000000	0.0000000	6
chr10:3160974|3162236	0.2664466	0.1467621	1.5645781	0.2616264	2
chr10:3174634|3175378	0.0000000	0.0000000	0.4644732	0.0000000	2
chr10:3180556|3181095	0.6539904	0.0000000	0.6967098	0.0000000	1
chr10:32197100|32199491	34.6069901	11.9805772	21.5980049	19.3603525	1

circRNA_coordinates	m6A-CTRL-S1	m6A-CTRL-S2	m6A-BTZ-S1	m6A-BTZ-S2	cluster_group
chr10:32740520|32762951	0.0968996	0.1711511	0.2322366	0.0000000	3
chr10:46121399|46143982	0.0000000	0.0000000	0.4644732	0.0000000	2
chr10:46224310|46274583	0.4087440	0.0000000	0.0000000	0.0000000	6
chr10:4872867|4884696	0.0000000	0.0000000	0.6967098	0.0000000	2
chr10:49941976|49951593	0.0000000	0.0000000	0.4644732	0.0000000	2
chr10:51630970|51633029	0.4904928	0.0855756	0.0000000	0.0000000	6
chr10:51829307|51840691	0.4632432	0.0000000	0.4644732	0.0000000	1
chr10:5768848|5773166	0.1634976	0.0000000	0.0000000	0.6540660	5
chr10:5815805|5842668	0.9537359	1.1124822	0.6967098	1.3081319	5
chr10:5827105|5842668	0.4087440	1.2836333	2.5546027	0.0000000	2
chr10:5836848|5842668	0.2452464	0.0202814	0.0000000	0.5232528	5
chr10:69692354|69752439	0.0000000	0.0000000	0.4129167	0.0000000	2
chr10:70218861|70229920	0.8992368	0.4278778	0.0000000	0.1308132	6
chr10:70547684|70548085	0.0000000	0.0000000	0.4644732	0.1308132	2
chr10:70719562|70720005	0.7902384	0.4278778	2.0901295	4.3168354	4
chr10:70719562|70721913	0.8174880	0.0000000	0.6967098	0.2616264	1
chr10:70719562|70726959	0.5449920	0.7701800	0.0000000	0.7848792	7
chr10:72604230|72619256	0.5092132	0.0000000	0.0000000	0.0000000	6
chr10:7262373|7423911	0.2724960	0.3423022	0.0000000	0.2616264	7
chr10:7285520|7327916	0.2997456	0.0000000	0.0000000	0.2616264	6
chr10:7285520|7423911	0.4359936	0.0000000	0.0000000	0.0000000	6
chr10:7318854|7327916	0.1907472	0.0855756	0.4644732	0.2616264	2
chr10:7318854|7423911	0.2997456	0.0234477	0.2654464	1.1773187	5
chr10:7409611|7412337	0.4554498	0.2567267	0.3095714	0.0000000	6
chr10:7409611|7423911	0.4359936	0.5990289	0.9289464	1.4389451	4

circRNA_coordinates	m6A-CTRL-S1	m6A-CTRL-S2	m6A-BTZ-S1	m6A-BTZ-S2	cluster_group
chr10:74100547|74100928	0.1021042	0.0241323	0.2933148	0.0000000	1
chr10:74468041|74475660	0.1937719	0.0299514	0.4888581	0.1569758	1
chr10:74897761|74899495	0.4971689	0.0000000	0.0000000	0.0000000	6
chr10:75331179|75336119	0.3542448	0.3423022	0.9289464	0.2616264	2
chr10:7839010|7844817	2.5342127	1.7115110	1.8578929	3.6627694	5
chr10:863665|866785	0.0000000	0.0000000	0.0000000	0.7474666	5
chr10:89685270|89712016	0.2724960	0.0000000	0.2322366	0.0000000	1
chr10:93609269|93611093	0.4632432	0.0000000	1.3418631	0.0000000	1
chr10:95259797|95259991	0.0000000	0.5134533	0.0000000	0.1308132	7
chr10:98408416|98416691	0.0000000	0.0000000	0.4644732	0.0000000	2
chr10:99120397|99123610	0.5177424	0.0000000	0.0000000	0.0000000	6
chr10:99967858|99969656	0.0000000	0.1711511	0.0000000	0.2616264	5
chr11:108057209|108061227	0.0000000	0.4171808	0.0000000	0.0000000	7
chr11:108137898|108138069	0.0000000	0.0000000	0.5866297	0.0000000	2
chr11:108559663|108594189	0.4851246	0.0000000	0.0000000	0.0000000	6
chr11:108586606|108594189	0.0000000	0.1711511	0.0000000	0.3924396	5
chr11:108593724|108594189	0.0817488	0.2567267	0.2322366	0.0000000	3
chr11:110007388|110030215	0.0000000	0.0000000	0.6967098	0.0000000	2
chr11:110118428|110124834	0.4087440	0.0000000	0.0000000	0.0000000	6
chr11:115047339|115049363	0.3814944	0.0000000	0.0000000	0.1308132	6
chr11:116627862|116629123	0.1362480	0.1711511	0.0000000	0.3924396	5
chr11:116629790|116636162	0.0000000	0.0855756	0.4644732	0.0000000	2
chr11:116633269|116636162	0.5177424	0.0000000	0.0000000	0.0000000	6
chr11:117023157|117034608	0.8447376	0.3423022	0.0000000	1.5697583	5
chr11:117109317|117110576	0.5994912	0.0000000	0.0000000	0.0000000	6

circRNA_coordinates	m6A-CTRL-S1	m6A-CTRL-S2	m6A-BTZ-S1	m6A-BTZ-S2	cluster_group
chr11:118425174|118430579	0.5177424	0.0000000	0.0000000	0.0523253	6
chr11:118650341|118657227	0.3814944	0.3423022	0.4644732	0.0000000	3
chr11:118656761|118657227	0.9264863	0.2567267	0.0000000	2.3546375	5
chr11:119002245|119003929	0.0000000	0.0299514	0.4644732	0.0000000	2
chr11:119003930|119004770	0.0000000	0.0000000	0.4888581	0.0000000	2
chr11:119155679|119156276	0.6539904	0.0000000	0.2322366	0.0000000	6
chr11:120916383|120930794	0.0000000	0.1711511	0.9289464	0.2616264	2
chr11:128628010|128642880	0.2997456	0.0000000	0.2322366	0.2616264	1
chr11:129739389|129740156	0.4480924	0.0000000	0.9289464	0.0000000	1
chr11:129793143|129807565	0.0000000	0.0855756	0.4644732	0.0000000	2
chr11:130130751|130131824	19.0474694	12.0661528	9.0572279	15.1743303	6
chr11:13435077|13466728	0.8447376	0.5134533	0.0000000	0.0000000	7
chr11:14486457|14504697	0.0000000	0.0000000	0.4644732	0.0000000	2
chr11:14793483|14840755	0.5449920	0.0000000	0.0000000	0.0000000	6
chr11:14852244|14882912	0.5449920	0.0000000	0.0000000	0.0000000	6
chr11:17190224|17191353	0.0544992	0.0000000	4.4124956	1.0465055	2
chr11:18312989|18313566	0.5177424	0.0000000	0.0000000	0.6540660	5
chr11:18312989|18314523	1.3079807	0.4278778	0.0000000	1.5697583	5
chr11:18557953|18566343	0.4087440	0.0000000	0.0000000	0.0000000	6
chr11:210001|210545	0.4904928	0.0000000	0.0000000	0.0000000	6
chr11:216745|218831	0.0000000	0.0000000	0.9289464	0.0000000	2
chr11:22225350|22261230	0.0000000	0.0855756	0.2933148	1.3081319	4
chr11:22225350|22284589	0.0272496	0.0000000	0.4644732	0.0000000	2
chr11:22242643|22261230	0.0000000	0.1711511	0.0000000	0.3924396	5
chr11:2997254|3000467	0.0000000	0.0000000	1.1611831	0.0000000	2

circRNA_coordinates	m6A-CTRL-S1	m6A-CTRL-S2	m6A-BTZ-S1	m6A-BTZ-S2	cluster_group
chr11:32410746|32413517	0.0000000	0.5134533	0.0000000	0.0000000	7
chr11:32413386|32413517	0.3814944	0.0855756	0.0000000	0.0000000	6
chr11:32948703|32956981	0.0000000	0.4278778	0.0000000	0.0000000	7
chr11:33307959|33309057	22.9714116	22.5919456	46.4473223	41.3369689	4
chr11:33307959|33350179	0.2997456	0.1711511	0.0000000	1.5697583	5
chr11:33368839|33370833	0.1634976	0.1711511	0.6967098	0.0000000	2
chr11:34084599|34084910	0.0000000	0.0000000	0.0000000	0.5232528	5
chr11:34093273|34098189	2.2072175	0.6846044	0.2322366	1.8313847	6
chr11:34093273|34101312	0.2452464	0.1711511	0.2322366	0.1308132	6
chr11:34952951|35006275	0.0000000	0.0000000	0.6967098	0.0000000	2
chr11:34952951|35013926	0.0000000	0.0000000	0.4644732	0.0000000	2
chr11:34978931|35006275	0.0000000	0.1711511	0.2837931	0.0000000	3
chr11:34978931|35013926	0.4632432	0.2567267	0.9289464	0.2616264	1
chr11:3723693|3733958	0.0000000	0.0000000	0.4644732	0.0000000	2
chr11:45883611|45893784	0.5449920	0.1711511	0.6967098	0.3924396	1
chr11:46098305|46113774	0.2029823	0.3302361	0.0977716	0.0374126	7
chr11:46391560|46392839	0.4087440	0.0000000	0.0000000	0.0000000	6
chr11:46529741|46534363	0.0000000	0.0000000	0.0000000	4.5223429	5
chr11:46771824|46773084	0.0000000	0.0000000	0.0000000	0.5232528	5
chr11:46829581|46842819	0.0000000	0.3423022	0.0000000	0.2093011	5
chr11:47380395|47397280	0.1907472	0.5258618	2.6454072	0.1374847	2
chr11:47380395|47397283	0.3814944	0.0898543	0.0000000	0.2990390	6
chr11:47444125|47444524	0.0000000	0.0000000	0.4129167	0.0000000	2
chr11:47504247|47505084	0.0000000	0.0000000	0.5504008	0.0000000	2
chr11:47647226|47648679	0.0000000	0.0000000	0.4644732	0.0000000	2

circRNA_coordinates	m6A-CTRL-S1	m6A-CTRL-S2	m6A-BTZ-S1	m6A-BTZ-S2	cluster_group
chr11:48146739|48149303	0.0000000	0.0000000	0.4644732	0.0000000	2
chr11:5717397|5719775	0.0000000	0.0000000	0.0000000	0.5232528	5
chr11:57258697|57259335	0.2724960	0.0000000	0.2322366	0.0000000	1
chr11:581492|582081	0.0000000	0.0000000	0.0000000	0.5232528	5
chr11:59426339|59426942	0.0000000	0.0000000	0.4644732	1.0465055	4
chr11:596923|598502	0.0000000	0.0000000	1.5172018	0.0000000	2
chr11:61895623|61898062	0.0000000	0.0000000	0.6967098	0.0000000	2
chr11:62303417|62304039	0.3814944	0.0000000	0.0000000	0.2616264	6
chr11:62603033|62603166	0.0000000	0.4278778	0.0000000	0.7848792	5
chr11:62606586|62607339	0.5177424	0.0478367	0.4644732	0.0000000	1
chr11:66133407|66137003	0.3269952	0.0000000	0.0000000	0.2616264	6
chr11:66407171|66407594	1.1717327	0.0855756	1.3934197	1.8313847	4
chr11:66947550|66999431	0.4087440	0.0000000	0.0000000	0.1308132	6
chr11:67012660|67013587	0.5271707	0.0000000	0.0000000	0.0000000	6
chr11:67953248|67957619	0.0363237	0.0000000	0.2322366	0.2616264	4
chr11:68029220|68030198	0.9537359	0.4278778	0.0000000	0.5232528	6
chr11:68548108|68552478	0.4152294	0.0000000	0.0000000	0.1308132	6
chr11:6953325|6964872	0.0000000	0.0000000	0.0000000	0.5232528	5
chr11:6953325|6977013	0.8992368	0.6846044	0.0000000	0.0000000	7
chr11:6962802|6977013	0.6539904	0.3423022	0.6967098	0.2616264	1
chr11:70178066|70185412	0.0000000	0.0000000	1.1611831	0.0000000	2
chr11:71693807|71701763	0.6576146	0.0000000	0.0000000	0.0000000	6
chr11:73418465|73429763	0.2802076	0.3423022	0.1955432	0.1569758	7
chr11:73418465|73429935	0.4087440	0.0521155	0.6967098	0.0000000	1
chr11:73843889|73844602	0.4632432	0.1390603	0.0000000	0.0000000	6

circRNA_coordinates	m6A-CTRL-S1	m6A-CTRL-S2	m6A-BTZ-S1	m6A-BTZ-S2	cluster_group
chr11:76174865|76183884	0.0000000	0.0000000	2.4772679	0.3924396	2
chr11:77330651|77340944	3.1609534	0.4278778	1.6256563	1.0465055	1
chr11:77336008|77336863	0.0000000	0.5134533	0.4644732	0.3924396	5
chr11:77830228|77832220	0.5994912	0.0000000	0.0000000	0.0000000	6
chr11:77936272|77937510	0.0000000	0.0000000	0.4644732	0.0000000	2
chr11:86778736|86802437	0.0000000	0.0000000	0.4644732	0.0000000	2
chr11:9424837|9430186	0.5799260	0.0000000	0.0000000	0.0000000	6
chr11:9451221|9452550	0.0000000	0.0000000	1.6182247	0.0000000	2
chr11:95546096|95546753	0.2179968	0.1711511	0.2322366	0.0000000	3
chr11:9735013|9770730	0.0000000	0.0000000	0.4644732	0.0000000	2
chr12:1003728|1006847	0.3814944	0.0000000	0.6967098	0.0000000	1
chr12:102153808|102183835	0.0000000	0.0000000	0.6967098	0.0000000	2
chr12:102154905|102160072	0.0000000	0.0000000	0.4644732	0.0000000	2
chr12:104370696|104374740	0.6267408	0.0000000	0.0000000	0.3924396	6
chr12:104714869|104725419	0.0000000	0.0000000	0.6967098	0.0000000	2
chr12:109046048|109048186	0.3390940	0.1564321	0.0000000	0.1308132	6
chr12:110017752|110019170	0.4087440	0.0000000	0.4644732	0.0000000	1
chr12:110345331|110346742	0.0000000	0.0000000	0.4644732	0.3924396	4
chr12:110819557|110834257	0.2179968	0.2567267	0.6967098	0.0000000	2
chr12:110922883|110925748	0.0000000	0.6846044	0.0000000	0.0000000	7
chr12:110974923|110975948	0.4632432	0.0000000	0.0000000	0.0000000	6
chr12:111855923|111856681	1.1989823	0.0000000	0.6967098	0.0000000	6
chr12:111951161|111957880	0.0000000	0.0847198	0.4644732	0.1308132	2
chr12:111990084|111993723	0.4087440	0.0000000	0.0000000	0.3924396	5
chr12:112303054|112308984	0.0000000	0.0000000	0.0000000	0.6540660	5

circRNA_coordinates	m6A-CTRL-S1	m6A-CTRL-S2	m6A-BTZ-S1	m6A-BTZ-S2	cluster_group
chr12:112578623|112580099	0.4632432	0.0000000	0.0000000	0.0000000	6
chr12:112757029|112757546	0.3269952	0.3423022	0.6967098	0.0000000	3
chr12:112884080|112893867	0.0000000	0.0000000	1.3934197	0.0000000	2
chr12:1136914|1137738	1.5259775	0.3423022	1.8578929	3.5319562	4
chr12:113715170|113715960	0.4087440	0.0000000	0.0000000	0.0000000	6
chr12:116534474|116675510	0.4904928	0.2567267	0.0000000	0.1308132	6
chr12:118619176|118619412	0.0000000	1.1402942	0.0000000	1.7733036	5
chr12:118627612|118639268	0.0000000	0.0000000	0.8258334	0.0000000	2
chr12:120156050|120168435	1.3378190	0.0000000	0.0000000	0.0000000	6
chr12:12022358|12022903	0.8992368	0.2567267	1.6256563	0.7848792	1
chr12:120565813|120567079	0.0000000	0.5134533	0.0000000	0.0000000	7
chr12:120592774|120593523	0.4087440	0.1711511	0.2322366	0.3924396	1
chr12:120599888|120600650	0.4632432	0.3423022	0.0000000	0.0000000	7
chr12:121840545|121855714	0.6539904	0.5134533	0.0000000	0.3924396	7
chr12:121853962|121855714	0.1907472	0.4278778	1.1611831	0.7848792	2
chr12:121878619|121891147	0.0000000	0.0000000	1.5998780	0.0000000	2
chr12:121932382|121972495	0.0000000	0.0000000	0.4644732	0.0000000	2
chr12:122242630|122255810	0.1089984	0.0000000	0.6967098	0.0000000	2
chr12:122242630|122265976	0.2997456	0.0855756	0.0000000	0.2616264	6
chr12:122255276|122255610	0.0000000	0.0000000	0.0000000	1.2500509	5
chr12:122261656|122263094	0.8174880	0.4278778	0.4644732	0.0000000	6
chr12:122825300|122826244	0.3814944	0.5990289	0.6967098	0.5232528	2
chr12:122861936|122865105	0.0000000	0.2567267	0.0000000	0.5232528	5
chr12:122992765|122999774	0.0000000	0.0000000	0.4644732	0.0000000	2
chr12:123076001|123078785	0.2997456	0.0000000	0.2322366	0.0000000	1

circRNA_coordinates	m6A-CTRL-S1	m6A-CTRL-S2	m6A-BTZ-S1	m6A-BTZ-S2	cluster_group
chr12:123694603|123707674	0.0000000	0.6846044	0.6967098	0.0000000	3
chr12:123702923|123707674	0.0000000	0.5990289	0.0000000	0.2093011	7
chr12:123705919|123707674	0.0000000	0.0855756	0.2322366	0.1308132	2
chr12:123798167|123800207	0.0000000	0.0000000	0.2752004	0.3924396	4
chr12:123825535|123830117	0.0000000	0.0000000	0.6967098	0.0000000	2
chr12:124071294|124074996	0.8992368	0.3423022	1.6256563	2.0930111	4
chr12:124856568|124862930	0.3542448	0.0000000	0.0000000	1.5697583	5
chr12:124904503|124915333	0.2997456	0.0000000	0.4644732	0.1308132	1
chr12:124911168|124922542	0.0000000	0.0000000	0.4644732	0.0000000	2
chr12:1289706|1399178	0.4087440	0.0000000	0.0000000	0.0000000	6
chr12:132237668|132250853	0.0000000	0.0000000	0.9289464	0.0000000	2
chr12:132479412|132491422	0.0000000	0.0000000	0.5675863	0.0000000	2
chr12:132575394|132593227	0.0000000	0.0000000	1.1611831	0.0000000	2
chr12:132588542|132606525	0.0817488	0.3423022	0.0000000	0.0000000	7
chr12:133250314|133251983	0.5177424	0.0000000	0.0000000	0.2616264	6
chr12:133306265|133306856	0.0000000	0.0855756	0.0000000	0.3924396	5
chr12:133310971|133313651	0.8858572	0.0000000	0.0000000	0.0000000	6
chr12:133331261|133331719	0.0000000	0.0000000	0.4644732	0.0000000	2
chr12:14576843|14578407	0.7084896	0.1711511	0.4644732	0.5232528	1
chr12:1863424|1863680	0.0000000	0.1711511	0.2322366	0.0000000	3
chr12:19615444|19626289	0.2724960	0.0000000	0.3834226	0.0000000	1
chr12:22796697|22826594	0.2997456	0.0000000	0.0000000	0.1308132	6
chr12:27867713|27888489	0.4904928	0.0000000	0.0000000	0.0000000	6
chr12:2931960|2946122	0.0000000	0.0000000	0.6967098	0.0000000	2
chr12:32458631|32459056	0.0000000	0.2567267	1.3934197	0.5232528	2

circRNA_coordinates	m6A-CTRL-S1	m6A-CTRL-S2	m6A-BTZ-S1	m6A-BTZ-S2	cluster_group
chr12:32480395|32481489	0.0000000	0.0000000	0.4644732	0.0000000	2
chr12:32729200|32735401	0.0000000	0.0000000	0.4644732	0.0000000	2
chr12:42768665|42792796	0.1089984	0.4171808	0.0000000	0.3924396	5
chr12:46319925|46322642	0.0000000	0.0000000	0.0000000	0.7848792	5
chr12:46622936|46637097	0.5449920	0.5990289	0.0000000	1.0465055	5
chr12:46633462|46637097	0.0000000	0.0855756	0.0000000	0.5232528	5
chr12:49237723|49239565	0.0272496	0.0000000	3.7157858	0.0000000	2
chr12:49419965|49421713	0.0000000	0.0000000	0.4644732	0.0000000	2
chr12:49436344|49442552	0.1089984	0.0000000	1.1611831	0.0000000	2
chr12:49447125|49447258	0.4359936	0.0000000	0.0000000	0.0000000	6
chr12:49854553|49884514	0.9537359	0.1711511	0.4644732	0.2616264	6
chr12:49854553|49893988	0.4359936	0.0000000	0.0000000	0.0000000	6
chr12:50385781|50386438	0.4359936	0.0000000	0.0000000	0.0000000	6
chr12:50488220|50490755	0.1907472	0.0000000	0.0000000	0.3139517	5
chr12:514677|527802	0.2997456	0.0299514	0.4644732	0.0000000	1
chr12:51497453|51497986	0.0000000	0.4389170	0.0000000	0.5108255	5
chr12:51685375|51686128	0.0000000	0.2567267	0.9289464	0.2616264	2
chr12:53410257|53416411	0.0000000	0.0000000	0.4644732	0.0000000	2
chr12:53415590|53421972	0.6267408	0.0000000	0.0000000	0.0000000	6
chr12:53427590|53432195	0.0000000	0.0000000	0.8516117	0.0000000	2
chr12:53848510|53853187	0.4359936	0.0000000	0.0000000	0.0000000	6
chr12:54109853|54110043	0.0000000	0.2567267	0.0000000	0.3924396	5
chr12:54639899|54646011	0.5164889	0.0000000	0.0000000	0.0000000	6
chr12:54922162|54925016	0.0000000	0.0000000	0.4644732	0.0000000	2
chr12:56182859|56197512	0.0964091	0.0318341	0.6193750	0.0498398	2

circRNA_coordinates	m6A-CTRL-S1	m6A-CTRL-S2	m6A-BTZ-S1	m6A-BTZ-S2	cluster_group
chr12:56563313|56563992	0.3542448	0.2567267	1.1611831	0.1308132	2
chr12:56742313|56743420	0.0000000	0.0000000	0.0000000	0.4401864	5
chr12:56748670|56749059	0.0000000	0.0000000	0.4644732	0.0000000	2
chr12:56956201|56965639	0.0000000	0.0855756	0.0000000	0.6540660	5
chr12:56997304|56998075	0.4765137	0.0000000	0.0000000	0.0000000	6
chr12:57059988|57064148	0.7629888	0.7701800	0.9289464	1.9621979	4
chr12:57868782|57869073	0.6267408	0.0000000	1.1611831	0.1308132	1
chr12:58142422|58142964	0.0968996	0.0000000	0.0000000	0.3924396	5
chr12:6443442|6450941	0.0000000	0.0000000	0.6967098	0.5232528	4
chr12:6657591|6657991	0.1175003	0.0000000	0.0000000	0.2990390	5
chr12:66597491|66611015	0.1634976	0.0855756	0.0515565	0.3924396	5
chr12:66597491|66622150	0.5722416	0.5134533	1.8578929	0.2616264	2
chr12:6780040|6781515	0.5722416	0.3423022	0.0000000	0.1308132	6
chr12:69210592|69222711	0.8447376	0.0000000	0.6967098	0.0000000	1
chr12:69644909|69656342	7.8206348	4.7922309	3.4835492	5.3633409	6
chr12:69983265|69987393	0.0474688	0.6846044	0.0000000	0.0000000	7
chr12:70193989|70195501	0.3814944	0.0855756	0.0000000	0.0523253	6
chr12:70671912|70704797	0.0000000	0.0000000	0.4644732	0.1308132	2
chr12:7076424|7076838	0.0703040	0.0000000	0.0000000	0.3924396	5
chr12:77191214|77199219	0.0000000	0.0000000	0.0000000	0.6540660	5
chr12:90049456|90049884	1.0082351	0.0855756	0.0000000	0.5232528	6
chr12:922808|939326	0.1907472	0.5586372	0.0000000	0.1308132	7
chr12:93192668|93196510	0.2724960	0.2567267	0.0000000	0.0000000	7
chr12:93244887|93246801	0.0000000	0.3423022	0.0000000	0.1308132	7
chr12:9450377|9459910	0.0000000	0.4278778	0.0000000	0.0000000	7

circRNA_coordinates	m6A-CTRL-S1	m6A-CTRL-S2	m6A-BTZ-S1	m6A-BTZ-S2	cluster_group
chr12:9577124|9586736	0.5722416	0.0000000	0.0000000	0.0000000	6
chr12:95876991|95897937	0.0000000	0.0000000	0.4644732	0.0000000	2
chr12:96704830|96728643	0.0000000	0.0000000	0.4644732	0.0000000	2
chr12:987378|1006847	0.0000000	0.0000000	0.6967098	0.0000000	2
chr12:98921664|98931350	0.2724960	0.0992676	0.0000000	0.2616264	5
chr13:103459496|103486902	0.0000000	0.0000000	0.6967098	0.1308132	2
chr13:113170754|113181798	1.5259775	0.1711511	1.6256563	0.2616264	1
chr13:113887491|113893865	0.0000000	0.0000000	0.4644732	0.0000000	2
chr13:113960800|113965182	0.0000000	0.0000000	0.6920651	0.0000000	2
chr13:114149817|114157903	0.4632432	0.0000000	0.0000000	0.0000000	6
chr13:114524022|114524930	0.0000000	0.2567267	0.0000000	0.2616264	5
chr13:114538629|114541041	0.4359936	0.0000000	0.6967098	0.0000000	1
chr13:114538630|114541041	0.0000000	0.4278778	0.0000000	0.0000000	7
chr13:114806476|114822949	0.0000000	0.5134533	0.0000000	0.0000000	7
chr13:114806476|114839312	0.2386792	0.0886563	0.0000000	0.1308132	6
chr13:114817527|114822949	0.1907472	0.0000000	0.2322366	0.0000000	1
chr13:114817527|114839312	0.2724960	0.0855756	1.2041468	0.0000000	2
chr13:20304379|20356931	0.8992368	0.5990289	1.6256563	2.4854507	4
chr13:20315705|20356931	0.0544992	0.0000000	0.4644732	0.3924396	4
chr13:20425495|20426330	0.0000000	0.0855756	0.0000000	0.3924396	5
chr13:20534098|20579379	0.0000000	0.0000000	0.6967098	0.1308132	2
chr13:20534098|20580726	0.0000000	0.0000000	0.9289464	0.1308132	2
chr13:20534098|20611049	0.0000000	0.0000000	0.9289464	0.0000000	2
chr13:20567203|20580726	0.5994912	0.0000000	0.0000000	0.0000000	6
chr13:20576990|20580726	0.0000000	0.0855756	0.0000000	0.3924396	5

circRNA_coordinates	m6A-CTRL-S1	m6A-CTRL-S2	m6A-BTZ-S1	m6A-BTZ-S2	cluster_group
chr13:20600752|20626451	0.0000000	0.0000000	0.0000000	0.5232528	5
chr13:21557363|21557945	0.0000000	0.0000000	0.6967098	0.0000000	2
chr13:21619824|21620369	0.0272496	0.4278778	0.0000000	0.1308132	7
chr13:21732061|21746820	0.0000000	0.3423022	0.0000000	0.5232528	5
chr13:21735929|21742538	0.7629888	0.3423022	0.0000000	0.9156923	5
chr13:21742127|21742538	22.4264196	4.6210798	3.0190760	8.2412311	6
chr13:21955573|21999817	0.0000000	0.2567267	0.2322366	0.0000000	3
chr13:23927924|23945304	0.5994912	0.8557555	1.3934197	1.0465055	2
chr13:24796957|24798720	0.9537359	0.2567267	0.2322366	0.5232528	6
chr13:24868877|24871873	0.0000000	0.0000000	0.9289464	0.0000000	2
chr13:25051839|25052414	0.6812400	0.4278778	0.0000000	0.9156923	5
chr13:26956951|26971362	0.4971689	0.0000000	0.0000000	0.0000000	6
chr13:26974590|26975761	0.0000000	0.0855756	0.0000000	0.3924396	5
chr13:28155434|28155940	0.2179968	0.2567267	0.0000000	0.5232528	5
chr13:28592604|28602425	0.4359936	0.0000000	0.0000000	0.5232528	5
chr13:28597487|28602425	0.1499818	0.0000000	0.0000000	0.5232528	5
chr13:28598998|28602425	1.0899839	0.1711511	0.0000000	0.3924396	6
chr13:28598998|28610180	0.0000000	0.0000000	0.4644732	0.0000000	2
chr13:28611322|28624359	0.0000000	0.0000000	0.0000000	0.6540660	5
chr13:28622412|28624359	0.4087440	0.0000000	0.0000000	0.1308132	6
chr13:28748409|28752072	0.1191080	0.0275553	0.3439424	0.3924396	4
chr13:28830429|28835595	0.1634976	0.2567267	0.0000000	0.1308132	7
chr13:28830429|28855516	0.9809855	0.0898543	0.0000000	0.2616264	6
chr13:30104675|30107119	0.6757900	0.0000000	0.0000000	0.0000000	6
chr13:30801549|30857928	0.0000000	0.0000000	0.0000000	0.5232528	5

circRNA_coordinates	m6A-CTRL-S1	m6A-CTRL-S2	m6A-BTZ-S1	m6A-BTZ-S2	cluster_group
chr13:31204972|31205611	2.0709695	0.4278778	1.3934197	1.5697583	1
chr13:32698423|32698818	0.0000000	0.0000000	1.1611831	0.0000000	2
chr13:32944539|32954282	0.0000000	0.0000000	0.4644732	0.0000000	2
chr13:33016525|33018263	0.4359936	0.2567267	0.0000000	0.3924396	7
chr13:33091994|33101669	1.5259775	0.3423022	1.6256563	2.3546375	4
chr13:33109906|33111164	2.0164703	3.3374465	0.0000000	2.2238243	7
chr13:41133646|41134997	0.5177424	0.0000000	0.0000000	0.0000000	6
chr13:41515057|41515506	1.3352303	0.0000000	0.9289464	0.7848792	1
chr13:41515057|41518061	0.0000000	1.5403599	0.0000000	2.2238243	5
chr13:41814397|41835051	1.0082351	0.0855756	0.0000000	0.2616264	6
chr13:41826781|41835051	0.6539904	0.5990289	0.0000000	0.1308132	7
chr13:41943226|41946966	0.2452464	0.0347437	0.0977716	0.0523253	6
chr13:42245082|42275666	0.0000000	0.0000000	0.4644732	0.0000000	2
chr13:42439872|42465723	0.0000000	0.0000000	0.9289464	0.0000000	2
chr13:45975218|45985615	0.0000000	0.1711511	0.0000000	0.3924396	5
chr13:46559432|46594692	1.0899839	0.5990289	1.1611831	0.6540660	1
chr13:46616299|46619648	0.5177424	0.0000000	0.0000000	0.0000000	6
chr13:49840015|49842195	0.1362480	0.0000000	0.6967098	0.0000000	2
chr13:50025689|50026045	0.2997456	0.0000000	0.0000000	0.1308132	6
chr13:50025689|50042096	1.0627343	0.0000000	0.0000000	0.0000000	6
chr13:50025689|50057699	0.0000000	0.0000000	0.4644732	0.0000000	2
chr13:50059766|50065046	0.4359936	0.0000000	0.0000000	0.0000000	6
chr13:50571210|50618904	0.2724960	0.2567267	0.0000000	0.2616264	7
chr13:51501543|51523641	0.1089984	0.5134533	1.3934197	0.9156923	2
chr13:52971367|52972329	0.4087440	0.0000000	0.0000000	0.0000000	6

circRNA_coordinates	m6A-CTRL-S1	m6A-CTRL-S2	m6A-BTZ-S1	m6A-BTZ-S2	cluster_group
chr13:53196124|53201713	0.0000000	0.0000000	0.0000000	0.6540660	5
chr13:60667762|60686320	0.0000000	0.0000000	0.0000000	0.6540660	5
chr13:61013822|61109367	0.0000000	0.0000000	0.6967098	0.0000000	2
chr13:72131156|72204849	0.0968996	0.0855756	0.0000000	0.2616264	5
chr13:73347822|73351631	0.4359936	0.0000000	0.2322366	0.0000000	6
chr13:73352325|73352518	0.0000000	0.1711511	0.0000000	0.2616264	5
chr13:75900333|75936743	0.5177424	0.0000000	0.0000000	0.0000000	6
chr13:76134889|76143643	1.1717327	0.0000000	0.0000000	0.7848792	6
chr13:77644766|77661768	0.0000000	0.0000000	0.6967098	0.0000000	2
chr13:77700453|77703365	0.5177424	0.1711511	0.0000000	0.2616264	6
chr13:77791975|77818086	0.0000000	0.0855756	0.0000000	0.3924396	5
chr13:77852893|77870809	0.0000000	0.3423022	0.0000000	0.5232528	5
chr13:95829961|95840796	0.0000000	0.5134533	0.0000000	0.0000000	7
chr13:95858786|95863035	0.0000000	0.0000000	0.9289464	0.0000000	2
chr13:99890681|99896878	0.0000000	0.0200247	0.3910865	0.3924396	4
chr13:99992574|100020160	0.7357392	0.0000000	0.0000000	0.0000000	6
chr14:102661275|102676199	1.0354847	0.0000000	0.0000000	0.0000000	6
chr14:103336522|103342862	0.4901385	0.1711511	0.0000000	0.0000000	6
chr14:103336522|103369766	0.0000000	0.0000000	0.4644732	0.0000000	2
chr14:103871413|103871604	0.1089984	0.0000000	0.4644732	0.0187063	2
chr14:105521724|105523617	0.4632432	0.1711511	0.0000000	0.0000000	6
chr14:105851325|105856272	0.4904928	0.0000000	0.0000000	0.0000000	6
chr14:20863609|20864889	0.0000000	0.8517335	0.0000000	0.0000000	7
chr14:20871536|20874559	0.0000000	0.1711511	0.2322366	0.0000000	3
chr14:21698478|21702388	0.4904928	0.1669579	0.1549018	0.5232528	5

circRNA_coordinates	m6A-CTRL-S1	m6A-CTRL-S2	m6A-BTZ-S1	m6A-BTZ-S2	cluster_group
chr14:21825356|21829372	0.2724960	0.6846044	0.0000000	0.0000000	7
chr14:21883733|21897494	0.3269952	0.0000000	0.4644732	0.0000000	1
chr14:21971316|21972024	11.1178362	6.0758642	10.4506475	8.8952971	1
chr14:23375404|23380612	0.9264863	0.0855756	2.3223661	0.7848792	1
chr14:23378692|23380612	0.0000000	0.4278778	0.0000000	3.4011430	5
chr14:23419523|23421892	0.1634976	0.0000000	0.0000000	0.3924396	5
chr14:23456729|23470246	0.0000000	0.0000000	0.4644732	0.0000000	2
chr14:24680621|24681035	0.0000000	0.0855756	0.6967098	0.1308132	2
chr14:31139462|31144271	0.3814944	0.0855756	1.3934197	0.0000000	2
chr14:31380983|31425448	0.0000000	0.0000000	0.4644732	0.0000000	2
chr14:31404369|31425448	0.4904928	0.9413311	1.1611831	0.6540660	3
chr14:31416296|31425448	0.2724960	0.0855756	0.0000000	0.2616264	5
chr14:31582310|31602881	0.0000000	0.2567267	0.9289464	0.1308132	2
chr14:31592120|31602881	0.0000000	0.0000000	0.4644732	0.0000000	2
chr14:31596991|31602881	0.4087440	0.0000000	3.7157858	0.7848792	2
chr14:31613317|31638674	0.0000000	0.7300450	0.0000000	0.0000000	7
chr14:31626046|31638674	0.0000000	0.0000000	0.4644732	0.0000000	2
chr14:35227910|35231429	0.0000000	0.0000000	0.6967098	0.9156923	4
chr14:35245125|35249516	0.4359936	0.0000000	0.0000000	0.3924396	5
chr14:35595941|35596817	0.0000000	0.0000000	0.1955432	0.2242138	4
chr14:39648295|39648666	3.0247054	0.4278778	0.6967098	1.7005715	6
chr14:45564424|45566208	0.0000000	0.4278778	0.0000000	0.0000000	7
chr14:50130033|50141145	0.0014170	0.0347437	0.6967098	0.0000000	2
chr14:50266193|50267414	0.9537359	0.0000000	0.0000000	0.0000000	6
chr14:50292585|50298079	0.0000000	0.0855756	0.4644732	0.0000000	2

circRNA_coordinates	m6A-CTRL-S1	m6A-CTRL-S2	m6A-BTZ-S1	m6A-BTZ-S2	cluster_group
chr14:55451441|55468903	0.0000000	0.0000000	0.6967098	0.0000000	2
chr14:57740962|57747140	0.5177424	0.0000000	0.0000000	0.0000000	6
chr14:58785260|58796887	1.5532271	0.5990289	0.6967098	0.7848792	6
chr14:60749402|60750255	0.3269952	0.3423022	0.2322366	0.0000000	3
chr14:60749402|60752459	0.4359936	0.0000000	0.0000000	0.2616264	6
chr14:60749402|60758172	0.8719872	0.7701800	0.2322366	0.7848792	7
chr14:64908172|64908751	0.4632432	0.0000000	0.0000000	0.0000000	6
chr14:66028055|66028484	4.3326862	1.3692088	5.1092055	2.7470770	1
chr14:67736418|67770316	0.0000000	0.0000000	0.0000000	0.5232528	5
chr14:68217769|68251994	0.0000000	0.0000000	0.0000000	0.5232528	5
chr14:71413632|71445365	0.0000000	0.5990289	0.0000000	0.0000000	7
chr14:71443659|71462642	0.4632432	0.0000000	0.0000000	0.0000000	6
chr14:73614503|73640415	0.4359936	0.4278778	0.6967098	0.0000000	3
chr14:73614503|73664837	0.5177424	0.0978129	0.0000000	0.0000000	6
chr14:74203701|74206958	0.0544992	0.2567267	0.0000000	0.1308132	7
chr14:75247108|75269379	0.4904928	0.2567267	0.4644732	0.0000000	6
chr14:75352289|75361112	0.4716361	0.0000000	0.0000000	0.0000000	6
chr14:76349028|76368567	0.0000000	0.0000000	0.0000000	0.5232528	5
chr14:77930941|77934851	0.0000000	0.4370343	0.0000000	0.0000000	7
chr14:81209419|81227957	0.4482831	0.0855756	0.4644732	0.1308132	1
chr14:81209419|81244390	0.9809855	0.5134533	0.0000000	0.9156923	7
chr14:81362062|81382906	0.4087440	0.0000000	0.0000000	0.0000000	6
chr14:90429452|90437615	0.2724960	0.0855756	0.6967098	0.1308132	1
chr14:90754561|90755489	0.0000000	0.0855756	0.4644732	0.0000000	2
chr14:91710515|91710640	0.0000000	0.0000000	0.4644732	0.0000000	2

circRNA_coordinates	m6A-CTRL-S1	m6A-CTRL-S2	m6A-BTZ-S1	m6A-BTZ-S2	cluster_group
chr14:91779424|91787648	0.0000000	0.0000000	0.9289464	0.0000000	2
chr14:91808724|91810001	0.0000000	0.0000000	0.6967098	0.0000000	2
chr14:91947920|91952074	0.5722416	0.0000000	0.0000000	0.0000000	6
chr14:93760204|93762503	2.3979647	0.7701800	2.0901295	1.0465055	1
chr14:97299804|97327072	0.4359936	0.0855756	0.2933148	1.7005715	5
chr14:99924616|99932150	2.8884574	1.6259355	2.3223661	2.3546375	1
chr15:100138635|100247060	0.4359936	0.0000000	0.0000000	0.0000000	6
chr15:101104897|101105470	0.1634976	0.0000000	0.6967098	0.6540660	4
chr15:101775287|101775782	0.0000000	0.0000000	0.4644732	0.5232528	4
chr15:23108752|23114292	0.4087440	0.4171808	3.4577709	0.6540660	2
chr15:23198510|23205198	0.0000000	0.0000000	0.0000000	0.7848792	5
chr15:25650608|25657118	0.4359936	0.0000000	0.0515565	0.0523253	6
chr15:28986256|28993021	0.2997456	1.0269066	0.0000000	0.0000000	7
chr15:28986256|29000043	1.7984735	1.3692088	0.0000000	3.4011430	5
chr15:28996634|29000043	0.0000000	0.0000000	1.1611831	0.0783571	2
chr15:29037080|29064144	1.4714783	0.0000000	0.0000000	1.5697583	5
chr15:30766559|30771396	0.2179968	0.4370343	0.0000000	0.0000000	7
chr15:30766559|30776889	3.7331950	1.3692088	1.6256563	3.5319562	5
chr15:32743565|32743801	1.7596701	0.0000000	0.0000000	0.0000000	6
chr15:32819014|32825569	3.1064542	2.2249643	3.0190760	1.0465055	6
chr15:40265109|40269045	0.4359936	0.0000000	0.0000000	0.0000000	6
chr15:40475915|40488975	0.0000000	0.0000000	0.4644732	0.0000000	2
chr15:40488746|40502411	0.0000000	0.0000000	0.4644732	0.0000000	2
chr15:41036245|41037457	1.4169791	0.3423022	0.9289464	0.0000000	6
chr15:41065925|41068825	0.0000000	0.0000000	0.8258334	0.0000000	2

circRNA_coordinates	m6A-CTRL-S1	m6A-CTRL-S2	m6A-BTZ-S1	m6A-BTZ-S2	cluster_group
chr15:41361768|41366763	0.0000000	0.0000000	0.6967098	0.0000000	2
chr15:41648237|41663882	0.4904928	0.0000000	0.0000000	0.0000000	6
chr15:41648237|41669502	0.2724960	0.5134533	0.0000000	0.1308132	7
chr15:41663725|41668026	0.0000000	0.0000000	0.4644732	0.0000000	2
chr15:41961026|41962156	20.1374533	6.8460441	10.4506475	15.1743303	1
chr15:41961026|42005694	0.4087440	0.0000000	0.0000000	0.0000000	6
chr15:41988273|41989221	0.3542448	0.0000000	0.0000000	0.2616264	6
chr15:41988273|41991357	5.6406669	3.4230221	5.3414421	5.2325277	1
chr15:41988273|42005694	1.3624799	0.5134533	2.0901295	0.6540660	1
chr15:42046635|42052727	0.1907472	0.3737084	0.0000000	0.0000000	7
chr15:43067333|43109295	0.8447376	0.2567267	0.9289464	0.2616264	1
chr15:43164809|43170882	0.2892545	0.3476079	0.2322366	0.1993593	7
chr15:43724392|43730614	0.0544992	0.0000000	0.0000000	0.5232528	5
chr15:43733724|43762264	0.4359936	0.3423022	0.6967098	0.1308132	3
chr15:43748090|43762264	0.7084896	0.0000000	0.6967098	0.9156923	1
chr15:44624186|44673174	0.8421761	0.0000000	0.0000000	0.0000000	6
chr15:44907561|44912601	0.0000000	0.0000000	0.0000000	0.5232528	5
chr15:44925703|44952814	0.0000000	0.0000000	0.4644732	0.0000000	2
chr15:49528048|49620327	0.0000000	0.0000000	0.9289464	0.0000000	2
chr15:50209131|50215691	0.0000000	0.0000000	0.4644732	0.0000000	2
chr15:50741597|50751359	0.4632432	0.0000000	0.0000000	0.0049709	6
chr15:50751197|50751359	0.5994912	0.0855756	0.0000000	0.1308132	6
chr15:50751197|50763992	0.0000000	0.1711511	0.6967098	0.0000000	2
chr15:50875286|50884823	0.1907472	0.2567267	0.2322366	0.5232528	5
chr15:50878589|50884823	0.2452464	0.3423022	0.0000000	0.1308132	7

circRNA_coordinates	m6A-CTRL-S1	m6A-CTRL-S2	m6A-BTZ-S1	m6A-BTZ-S2	cluster_group
chr15:50884108|50884823	0.0000000	0.4278778	0.2322366	0.0000000	3
chr15:52338027|52350994	0.4904928	0.0000000	0.0000000	0.0000000	6
chr15:56970862|56974675	0.0000000	0.0000000	0.9289464	0.0000000	2
chr15:59179174|59179739	0.6267408	0.4278778	0.2322366	0.1308132	6
chr15:59189314|59209198	0.0000000	0.0000000	0.6967098	0.0000000	2
chr15:59204762|59205895	0.3814944	0.0238756	0.0000000	0.0000000	6
chr15:59204762|59209198	7.1393948	2.4816910	6.5026251	8.7644839	1
chr15:59323003|59323901	1.2807311	0.1711511	0.6967098	0.1308132	6
chr15:59323003|59350749	0.1362480	0.1711511	0.2322366	0.0000000	3
chr15:59323003|59359282	0.0000000	0.0000000	0.0515565	0.3924396	4
chr15:59323003|59368414	0.4632432	0.2567267	0.0000000	0.3924396	7
chr15:63988323|64008672	0.2452464	0.3423022	1.8578929	1.3081319	2
chr15:63998979|64008672	0.5449920	0.6846044	0.4644732	0.0000000	3
chr15:64056295|64067848	0.0000000	0.0000000	0.4644732	0.1308132	2
chr15:64066893|64067848	0.0000000	0.0000000	0.0000000	0.6540660	5
chr15:64448361|64448923	2.4677509	0.0000000	0.1955432	0.0000000	6
chr15:64543711|64592922	0.0000000	0.0000000	0.0000000	0.7848792	5
chr15:64551400|64592922	0.4359936	0.0855756	0.0000000	0.0000000	6
chr15:64791492|64792365	37.1412028	23.9611544	46.4473223	52.1944638	4
chr15:64791492|64915251	0.2179968	0.2567267	0.0000000	0.5232528	5
chr15:65115977|65116553	0.2179968	0.0000000	0.2322366	0.9156923	5
chr15:65471272|65472542	0.1332233	0.0000000	0.1161183	0.2616264	4
chr15:65790202|65793117	0.9537359	0.0855756	0.4644732	0.1619467	6
chr15:65847225|65851069	0.0000000	0.0000000	0.0000000	0.5232528	5
chr15:66007816|66048810	0.0000000	0.0000000	0.4644732	0.0000000	2

circRNA_coordinates	m6A-CTRL-S1	m6A-CTRL-S2	m6A-BTZ-S1	m6A-BTZ-S2	cluster_group
chr15:66021410|66031213	0.0847735	0.0000000	0.4542548	0.0000000	2
chr15:66044717|66053776	0.1634976	0.0855756	0.1955432	0.0000000	1
chr15:66048478|66053776	0.4904928	0.0000000	0.0000000	0.0000000	6
chr15:66641398|66641775	0.5722416	0.0000000	0.2322366	0.7848792	4
chr15:66782102|66782555	0.6267408	0.0000000	0.0000000	0.0000000	6
chr15:68378644|68380228	0.0000000	0.0855756	2.0901295	0.0000000	2
chr15:68378644|68439038	0.4359936	0.0000000	0.0000000	0.0000000	6
chr15:68378644|68446033	0.0000000	0.0000000	0.6967098	0.0000000	2
chr15:69502655|69553668	0.6267408	0.0000000	0.0000000	0.5232528	6
chr15:72300167|72338975	0.0000000	0.0000000	0.2322366	0.6540660	4
chr15:72324835|72338975	0.8992368	0.2567267	0.0000000	1.1773187	5
chr15:72338065|72338975	1.9347215	1.2836333	0.0000000	1.9621979	7
chr15:73064124|73067438	0.0421824	0.2607487	0.0000000	0.1308132	7
chr15:75703833|75705386	0.0000000	0.1711511	0.2322366	0.1308132	2
chr15:75782549|75819602	0.2724960	0.0000000	0.6967098	0.0000000	1
chr15:76152219|76165909	0.2452464	0.3423022	0.0000000	0.5232528	5
chr15:77471132|77474382	0.0000000	0.2567267	0.0000000	0.9156923	5
chr15:78305165|78310299	0.6725201	0.5134533	0.0000000	0.0000000	7
chr15:78557099|78566766	0.0000000	0.0000000	1.2385178	0.0000000	2
chr15:80390758|80415142	0.3269952	0.2567267	0.4644732	0.0000000	3
chr15:82443806|82444764	0.0000000	0.4278778	0.0000000	0.0000000	7
chr15:85081763|85098301	0.0000000	0.1711511	0.0000000	0.2616264	5
chr15:85223944|85234875	0.2179968	0.1711511	1.3934197	0.1046506	2
chr15:85230856|85234875	0.2179968	0.0000000	0.2322366	0.0124273	1
chr15:86118362|86129054	0.2724960	0.0000000	0.0000000	0.1308132	6

circRNA_coordinates	m6A-CTRL-S1	m6A-CTRL-S2	m6A-BTZ-S1	m6A-BTZ-S2	cluster_group
chr15:90610342|90611827	0.0000000	0.0000000	0.9289464	0.0000000	2
chr15:90982564|90986710	0.5574178	0.0855756	0.6967098	0.0000000	1
chr15:90984738|90986710	0.5177424	0.0000000	1.7029911	0.0041860	1
chr15:91017717|91030321	0.0000000	0.0000000	0.4644732	0.0000000	2
chr15:91030186|91035943	0.4359936	0.1711511	0.0000000	0.0000000	6
chr15:91310140|91312816	0.0000000	0.0000000	0.4644732	0.0000000	2
chr15:91519906|91525211	0.5177424	0.2567267	0.4644732	0.3924396	1
chr15:91524114|91525211	0.8719872	0.2567267	0.0000000	0.6540660	6
chr15:93467551|93472321	0.4087440	0.0299514	0.1955432	0.0000000	6
chr15:93540187|93541851	0.2724960	0.0000000	0.0000000	0.1308132	6
chr15:93543742|93558139	0.3814944	0.0000000	0.0000000	0.9156923	5
chr15:93545407|93558139	0.0000000	0.0000000	0.4644732	0.0000000	2
chr15:93552375|93563488	0.3269952	0.0000000	0.2322366	0.6540660	4
chr16:10524468|10525312	0.3814944	0.3423022	0.0000000	0.3924396	7
chr16:11114050|11154879	0.0000000	0.0451839	0.4644732	0.1046506	2
chr16:11114050|11220003	0.5449920	0.6846044	0.0000000	0.5232528	7
chr16:11988811|11990642	0.5890546	0.0000000	0.6967098	0.3924396	1
chr16:14028049|14029600	0.5722416	0.3423022	0.0000000	0.3924396	7
chr16:14687158|14698083	0.0000000	0.1711511	0.9289464	0.3924396	2
chr16:14738131|14738466	0.5722416	0.0000000	0.0000000	0.0000000	6
chr16:15727474|15733148	0.2997456	0.0000000	0.0000000	0.1308132	6
chr16:1675974|1691188	0.0000000	0.0000000	0.0000000	1.5136395	5
chr16:17228330|17353355	0.0000000	0.7701800	0.0000000	0.0000000	7
chr16:17352845|17353355	2.3434655	0.6846044	1.1611831	2.2238243	1
chr16:18844262|18856973	0.0000000	0.0000000	0.9289464	0.0000000	2

circRNA_coordinates	m6A-CTRL-S1	m6A-CTRL-S2	m6A-BTZ-S1	m6A-BTZ-S2	cluster_group
chr16:18846214|18856973	0.0000000	0.5990289	0.4644732	0.0000000	3
chr16:18852887|18856973	2.4252143	0.6846044	0.9289464	0.5232528	6
chr16:19085243|19088696	0.0000000	0.0000000	0.0000000	0.5232528	5
chr16:19547262|19552109	0.4359936	0.0000000	0.0000000	0.0000000	6
chr16:2052755|2052991	3.7965231	0.0608442	0.0000000	0.0746943	6
chr16:22319469|22320831	0.1285636	0.5446884	0.0000000	0.0000000	7
chr16:2312279|2314761	0.2997456	0.0000000	0.2322366	0.1308132	1
chr16:24043457|24046868	0.1701192	0.0000000	0.5882553	0.0000000	1
chr16:24762047|24788679	0.0000000	0.1711511	0.4644732	0.0000000	2
chr16:2616357|2627501	0.6812400	0.0000000	0.0000000	0.0000000	6
chr16:27760905|27763245	0.2452464	0.0000000	0.4644732	0.0000000	1
chr16:2807473|2810500	0.0817488	0.0855756	0.4644732	0.1308132	2
chr16:28399379|28403463	0.3269952	0.0000000	0.0000000	0.1308132	6
chr16:28399829|28403463	0.0000000	0.0000000	0.4644732	0.0000000	2
chr16:28970038|28970421	0.6297382	0.0000000	0.0000000	0.0000000	6
chr16:30485620|30486606	0.4632432	0.0000000	0.0000000	0.0000000	6
chr16:30486627|30492905	2.9035537	0.0000000	0.0000000	0.0000000	6
chr16:30490412|30490782	0.2997456	0.0000000	0.4301022	0.3563351	1
chr16:30495148|30495584	1.5259775	0.1711511	0.2322366	0.6540660	6
chr16:30515496|30516783	0.0000000	0.0000000	0.7905334	0.1016419	2
chr16:30530041|30531144	0.0000000	0.0000000	0.4644732	0.0000000	2
chr16:30531289|30532789	0.1634976	0.0000000	1.1611831	0.0000000	2
chr16:30594790|30596442	0.0000000	0.4278778	0.0000000	0.0000000	7
chr16:30675536|30677862	0.3269952	0.0855756	0.0000000	0.1308132	6
chr16:30675536|30678943	0.0544992	0.0000000	0.2322366	0.2616264	4

circRNA_coordinates	m6A-CTRL-S1	m6A-CTRL-S2	m6A-BTZ-S1	m6A-BTZ-S2	cluster_group
chr16:3185389|3187140	0.0000000	0.0000000	0.4644732	0.0000000	2
chr16:347056|348251	1.7439743	0.5134533	0.0000000	0.0000000	6
chr16:3600523|3602195	0.0000000	0.0000000	0.2933148	0.1308132	2
chr16:3786037|3795355	0.3980621	0.0000000	0.2322366	0.0000000	6
chr16:3900298|3901010	8.0113820	1.8826621	4.4124956	6.2790332	1
chr16:396148|397106	1.2807311	0.5134533	1.6256563	0.6540660	1
chr16:4311780|4312702	2.8884574	2.6528421	2.3223661	2.7470770	7
chr16:4516154|4519466	0.4359936	0.2567267	1.8578929	0.0000000	2
chr16:4555485|4557013	0.6327629	0.0000000	0.0000000	0.0000000	6
chr16:4555485|4559539	0.4632432	0.0000000	0.0000000	0.2616264	6
chr16:4700366|4707364	0.0253149	0.0000000	0.3834226	0.0000000	2
chr16:47143394|47165936	0.2452464	0.0855756	0.0000000	0.2616264	5
chr16:48580058|48596355	0.2452464	0.0855756	0.2322366	0.3924396	4
chr16:4918834|4921302	0.0000000	0.0000000	0.4644732	0.0000000	2
chr16:50709681|50711446	0.0000000	0.0000000	1.8578929	0.0000000	2
chr16:53269097|53289691	0.0000000	0.0000000	0.9289464	0.0000000	2
chr16:53288350|53308214	0.0000000	0.5134533	0.2322366	0.5232528	5
chr16:53289512|53308214	0.1362480	0.3423022	0.0000000	0.2616264	5
chr16:57050986|57054919	0.0000000	0.0000000	0.4644732	0.0000000	2
chr16:57149463|57151378	0.4087440	0.0000000	0.0000000	0.0000000	6
chr16:57197913|57207781	9.0468667	4.0220509	7.4315716	0.0000000	6
chr16:57201005|57207781	0.0544992	0.0000000	0.0000000	0.5232528	5
chr16:57238416|57250947	0.5994912	0.0000000	0.0000000	0.2616264	6
chr16:57238416|57255327	0.2724960	0.0000000	0.2322366	0.0000000	1
chr16:58594116|58594266	0.0000000	0.0000000	0.0000000	0.6540660	5

circRNA_coordinates	m6A-CTRL-S1	m6A-CTRL-S2	m6A-BTZ-S1	m6A-BTZ-S2	cluster_group
chr16:67605051|67655494	0.4087440	0.0000000	0.0000000	0.0000000	6
chr16:67644727|67646024	3.3517006	1.7970866	1.8578929	1.0465055	6
chr16:67644727|67650781	0.4087440	0.0000000	0.4644732	0.0000000	1
chr16:67644727|67655494	0.0000000	0.5134533	0.0000000	0.0000000	7
chr16:67662273|67663436	0.4632432	0.0000000	0.0000000	0.0000000	6
chr16:68155890|68157024	0.9809855	3.0807199	2.0901295	1.4389451	3
chr16:68155890|68160513	9.1558651	6.7604686	13.2374869	13.2121325	4
chr16:68191772|68217269	0.0000000	0.6692008	0.0000000	0.1869321	7
chr16:68191772|68225678	0.5994912	0.1711511	0.0000000	0.3924396	6
chr16:68321649|68325564	0.0000000	0.0000000	0.6709316	0.0000000	2
chr16:68893787|68896964	0.1362480	0.0000000	0.0000000	0.9156923	5
chr16:68893787|68901123	0.4904928	0.0000000	0.0000000	0.0000000	6
chr16:69404386|69406258	0.0070304	0.0000000	0.4644732	0.3924396	4
chr16:69782135|69786274	0.5722416	0.0000000	0.0000000	0.0000000	6
chr16:69971556|69972901	0.1089984	0.0000000	0.4644732	0.0000000	2
chr16:70726899|70729446	0.0000000	0.0000000	0.6967098	0.0000000	2
chr16:71772845|71784235	0.0000000	0.0000000	0.0000000	0.5232528	5
chr16:71779047|71779517	0.0000000	0.0000000	1.0838483	0.0000000	2
chr16:72651082|72651452	0.2785454	0.0000000	0.0000000	0.2616264	5
chr16:74685818|74695349	0.0000000	0.2567267	0.2322366	0.0000000	3
chr16:80718435|80719026	3.0247054	0.9413311	0.9289464	1.8313847	6
chr16:84766659|84779279	0.0000000	0.0000000	0.6967098	0.0000000	2
chr16:84767041|84779279	0.7629888	0.0000000	0.2322366	0.0000000	6
chr16:84773915|84779279	3.5151982	2.3105399	4.1802590	2.7470770	1
chr16:84796595|84808831	0.1634976	0.0000000	0.6967098	0.0000000	2

circRNA_coordinates	m6A-CTRL-S1	m6A-CTRL-S2	m6A-BTZ-S1	m6A-BTZ-S2	cluster_group
chr16:85667520|85667738	3.2699518	1.6259355	3.7157858	3.2703298	1
chr16:88675342|88677944	0.4087440	0.1711511	0.2322366	0.0000000	6
chr16:88677676|88677944	0.2997456	0.0000000	0.0000000	0.1308132	6
chr16:88893274|88898405	1.0899839	0.0000000	0.0000000	0.0000000	6
chr16:89164999|89169167	0.2452464	0.0855756	0.6967098	0.0000000	1
chr16:89167070|89169167	0.2724960	0.0000000	0.0000000	0.5232528	5
chr16:89167070|89180895	0.7084896	0.0000000	0.2322366	0.0000000	6
chr16:89371614|89383486	0.1089984	0.0000000	1.3762342	0.6540660	2
chr16:8952207|8953192	0.0000000	0.0000000	1.9610059	0.0000000	2
chr16:89592877|89595853	0.0000000	0.0000000	0.0000000	0.5232528	5
chr16:89836692|89836971	0.0000000	0.0000000	0.9289464	0.1308132	2
chr16:89949759|89954125	0.0000000	0.0855756	0.8945754	0.0000000	2
chr16:89961446|89962491	0.7954703	0.0000000	0.0000000	0.0000000	6
chr17:11958206|11984847	0.6812400	0.0000000	0.6967098	0.7848792	1
chr17:11958206|11999011	0.1574482	0.0000000	0.2322366	0.1308132	1
chr17:1339914|1340449	1.2262319	0.0000000	0.6967098	0.0000000	6
chr17:15449099|15458678	1.8982615	0.0000000	0.0000000	0.0124273	6
chr17:15619373|15620627	0.0000000	0.5134533	0.0000000	0.9156923	5
chr17:15942747|15969005	0.0000000	0.4278778	0.0000000	0.0000000	7
chr17:15989596|16005119	0.0000000	0.0000000	0.4644732	0.0000000	2
chr17:16004564|16005119	2.9974558	0.5134533	0.6967098	1.4389451	6
chr17:1628081|1628281	0.0000000	0.0000000	0.9547247	0.0000000	2
chr17:1703151|1704318	0.2179968	0.2567267	0.0000000	0.0000000	7
chr17:17045941|17046983	0.7866687	0.0000000	0.0000000	0.0000000	6
chr17:17075032|17083402	0.0544992	0.3423022	0.3999114	0.0000000	3

circRNA_coordinates	m6A-CTRL-S1	m6A-CTRL-S2	m6A-BTZ-S1	m6A-BTZ-S2	cluster_group
chr17:17722236|17722326	0.0000000	0.0000000	0.4644732	0.0000000	2
chr17:18195959|18198099	0.0000000	0.0000000	0.6193750	0.0000000	2
chr17:18417903|18422988	1.1444831	0.0000000	0.0000000	0.0000000	6
chr17:18482971|18483924	0.0000000	0.4278778	0.0000000	0.0000000	7
chr17:1937137|1939237	0.0000000	0.0000000	0.9289464	0.0000000	2
chr17:19823349|19861884	0.0000000	0.4278778	0.0000000	0.0000000	7
chr17:19835118|19861884	0.6539904	0.1711511	0.0000000	0.2616264	6
chr17:19839599|19861884	0.2452464	0.0855756	0.4644732	0.1308132	1
chr17:19843026|19861884	0.5722416	0.0000000	0.0000000	0.0000000	6
chr17:19843026|19871774	0.8719872	0.0000000	0.0000000	0.0000000	6
chr17:19861327|19861884	4.4689342	2.1393888	3.7157858	2.8778902	1
chr17:20107646|20109225	5.2046733	5.6479864	5.5736787	7.8487916	5
chr17:20852416|20861715	0.0000000	0.2567267	0.6967098	0.0000000	2
chr17:2239060|2239624	0.0000000	0.0000000	0.4644732	0.0000000	2
chr17:2297337|2298748	6.8123996	2.0538132	4.6447322	7.1947256	1
chr17:25630393|25636298	0.4904928	0.0000000	0.0000000	0.0000000	6
chr17:25630393|25638642	0.2724960	0.3423022	0.0000000	0.0000000	7
chr17:26918716|26920084	0.6539904	0.5134533	0.0000000	0.0000000	7
chr17:26944365|26945808	0.0000000	0.0000000	0.9289464	0.0000000	2
chr17:27228511|27229719	0.0000000	0.0000000	0.6967098	0.0000000	2
chr17:27235801|27248826	0.0000000	0.0000000	0.6967098	0.0000000	2
chr17:27237244|27241055	0.0000000	0.1711511	0.0000000	0.3924396	5
chr17:27248706|27251320	0.3814944	0.0000000	0.2322366	0.0000000	6
chr17:27248706|27254081	1.3897295	0.3423022	0.2322366	1.3081319	5
chr17:27419512|27419872	1.0967691	0.0898543	0.0000000	0.0000000	6

circRNA_coordinates	m6A-CTRL-S1	m6A-CTRL-S2	m6A-BTZ-S1	m6A-BTZ-S2	cluster_group
chr17:27613010|27614734	1.6622255	1.1124822	1.3934197	1.1773187	6
chr17:27778473|27794234	0.1453221	0.0000000	0.2933148	0.0498398	1
chr17:27778473|27838010	0.2452464	0.0000000	0.2322366	0.0000000	1
chr17:27844471|27861318	0.4632432	0.0000000	0.0000000	0.0000000	6
chr17:27857425|27861318	0.0000000	0.5909848	0.0000000	0.0000000	7
chr17:27975162|27999147	0.1634976	0.0855756	0.6967098	0.1046506	2
chr17:28808161|28817235	0.0000000	0.0000000	0.6502625	0.0000000	2
chr17:29061915|29086557	0.6267408	0.2567267	0.0000000	0.6540660	5
chr17:33310021|33310571	0.1089984	0.0000000	0.0000000	0.3924396	5
chr17:33310021|33313150	0.8174880	0.0000000	0.6967098	0.0000000	1
chr17:33689758|33690845	1.0899839	0.5134533	0.0000000	0.7848792	7
chr17:33772350|33772674	0.0000000	0.0000000	0.4644732	0.0000000	2
chr17:33951416|33954745	0.5994912	0.0000000	0.0000000	0.0000000	6
chr17:33951416|34009843	0.0000000	0.0000000	0.4644732	0.0000000	2
chr17:34867319|34869271	0.5177424	0.5134533	0.9289464	0.0000000	3
chr17:35310186|35311207	0.6812400	0.7701800	1.3934197	1.8313847	4
chr17:35640168|35646430	0.8436748	0.0000000	0.0000000	0.0000000	6
chr17:35913217|35914161	0.0000000	0.1711511	1.3934197	0.3924396	2
chr17:35944756|35945538	0.0000000	0.0299514	0.4644732	0.0000000	2
chr17:36353601|36359042	0.7638335	0.0000000	0.0000000	0.0000000	6
chr17:36969048|36971350	0.0000000	0.0000000	0.6967098	0.0000000	2
chr17:3717616|3719564	0.0000000	0.0000000	0.0000000	0.6353597	5
chr17:37646810|37650947	0.0000000	0.0000000	0.4129167	0.0000000	2
chr17:37646810|37657692	0.2452464	0.0000000	0.6967098	0.0000000	1
chr17:37646810|37682569	0.7629888	0.3423022	0.4644732	0.0000000	6

circRNA_coordinates	m6A-CTRL-S1	m6A-CTRL-S2	m6A-BTZ-S1	m6A-BTZ-S2	cluster_group
chr17:38412643|38433436	0.0000000	0.1868970	1.3934197	0.0000000	2
chr17:38551701|38555383	0.2724960	0.1711511	0.2322366	1.0465055	5
chr17:3967655|3968123	0.0272496	0.0000000	0.9289464	0.0000000	2
chr17:3969741|3976050	0.1054559	0.0000000	0.2322366	0.2616264	4
chr17:40025873|40027941	0.1907472	0.2567267	0.2322366	0.0000000	3
chr17:41150849|41151925	0.6267408	0.0000000	0.0000000	0.0000000	6
chr17:4186093|4200109	0.2997456	0.0000000	0.0000000	0.3139517	5
chr17:42161923|42165064	0.0000000	0.1711511	0.4644732	0.0000000	2
chr17:42501717|42552271	0.2058980	0.3423022	0.0000000	0.0000000	7
chr17:43480080|43483449	0.0000000	0.0000000	0.9289464	0.0000000	2
chr17:43552466|43553092	0.7629888	0.2567267	0.0000000	0.0000000	6
chr17:43552466|43555513	0.0000000	0.1711511	0.9289464	1.5697583	4
chr17:4445210|4445758	0.4087440	0.0000000	0.0000000	0.0000000	6
chr17:45734226|45736026	0.4170279	0.0000000	0.0000000	0.0000000	6
chr17:45741525|45752148	0.5449920	0.0000000	0.0000000	0.0000000	6
chr17:4585899|4586097	0.4087440	0.0000000	0.0000000	0.0000000	6
chr17:46189393|46190763	0.2452464	0.0599029	0.0000000	0.1308132	6
chr17:463666|565135	0.0000000	0.0000000	0.6967098	0.0000000	2
chr17:48216628|48218738	0.8141635	0.0000000	0.0000000	0.1308132	6
chr17:49091593|49098726	0.3269952	0.0000000	0.0000000	0.1308132	6
chr17:49340635|49346265	0.1907472	0.0978129	0.0977716	0.0872524	6
chr17:5253746|5266311	0.0000000	0.0855756	0.4644732	0.0000000	2
chr17:5257654|5266311	0.0272496	0.0855756	0.6967098	0.0000000	2
chr17:5257654|5271763	0.4359936	0.0855756	0.0000000	0.1308132	6
chr17:5264503|5266311	0.8174880	0.0855756	0.0000000	0.0000000	6

circRNA_coordinates	m6A-CTRL-S1	m6A-CTRL-S2	m6A-BTZ-S1	m6A-BTZ-S2	cluster_group
chr17:5264503|5276749	1.1717327	0.2567267	0.0000000	0.7848792	6
chr17:5364259|5365866	0.1634976	0.5134533	0.0000000	1.5697583	5
chr17:56382748|56383004	0.5523766	0.0000000	0.0000000	0.0000000	6
chr17:56383804|56384160	0.1937719	0.0000000	0.0000000	0.2491991	5
chr17:56387328|56396655	0.5177424	0.0000000	0.0000000	0.0000000	6
chr17:57138393|57158580	0.0000000	0.0000000	0.6967098	0.0000000	2
chr17:57274905|57275150	0.1634976	0.2567267	0.2322366	0.1370922	3
chr17:57430576|57430887	0.6267408	0.3423022	0.3910865	0.1308132	6
chr17:57808782|57816308	0.1907472	0.1711511	0.4644732	0.3924396	4
chr17:58258685|58268044	0.0000000	0.0000000	0.0000000	0.5232528	5
chr17:58700882|58725443	0.1695470	0.0000000	0.2322366	0.0000000	1
chr17:59853762|59886118	0.2452464	0.5134533	0.0000000	0.0000000	7
chr17:60069901|60088594	0.0000000	0.0000000	0.0000000	0.5232528	5
chr17:60087911|60140662	0.0000000	0.0000000	0.0000000	0.7848792	5
chr17:60741909|60742310	0.0000000	0.0000000	0.0000000	0.5232528	5
chr17:62248460|62265775	0.0000000	0.6779295	0.0000000	0.0332266	7
chr17:62270919|62272455	0.0544992	0.1711511	0.4644732	0.0000000	2
chr17:62289934|62291602	1.8529727	1.4547844	0.9289464	0.3924396	6
chr17:62747925|62760666	0.0000000	0.0299514	0.0000000	0.6540660	5
chr17:62817885|62820990	0.2179968	0.0000000	0.2322366	0.0000000	1
chr17:65887960|65890281	0.8447376	0.0000000	0.6967098	0.5232528	1
chr17:65941525|65944422	0.8447376	0.7701800	0.9289464	0.0000000	3
chr17:65941525|65960520	0.7084896	0.5134533	0.6967098	0.0000000	3
chr17:65941525|65972074	1.9892207	0.8557555	2.5546027	1.7005715	1
chr17:73238417|73239651	0.4087440	0.0000000	0.0000000	0.0000000	6

circRNA_coordinates	m6A-CTRL-S1	m6A-CTRL-S2	m6A-BTZ-S1	m6A-BTZ-S2	cluster_group
chr17:73615557|73615736	0.0000000	0.2567267	0.2322366	0.0000000	3
chr17:73689520|73700894	1.6632337	0.0000000	0.0000000	0.0000000	6
chr17:74308951|74309972	1.8802223	0.0855756	0.0000000	0.6540660	6
chr17:74381736|74382049	0.0000000	0.0000000	0.4644732	0.0000000	2
chr17:74714815|74718015	0.5423488	0.0000000	0.0000000	0.0000000	6
chr17:75398141|75398785	1.7712239	0.4278778	0.0000000	1.0465055	6
chr17:76198580|76198832	0.0000000	0.1535225	0.5160298	0.0000000	2
chr17:76388557|76400170	0.3269952	0.0000000	0.0000000	0.1308132	6
chr17:76798406|76800060	0.0000000	0.0000000	0.0000000	0.6540660	5
chr17:78184828|78185869	0.0000000	0.0000000	0.9289464	0.0000000	2
chr17:79203293|79204343	0.7357392	0.0000000	0.0000000	0.0000000	6
chr17:79532531|79539114	0.4904928	0.0000000	0.0000000	0.0000000	6
chr17:79571574|79575848	0.9039237	0.0000000	1.1611831	0.0971942	1
chr17:79571574|79596831	0.0000000	0.0000000	0.0000000	0.5232528	5
chr17:79580344|79589304	0.0000000	0.0000000	0.4972186	0.0000000	2
chr17:79653342|79664028	0.0000000	0.0000000	0.4386950	0.0000000	2
chr17:79654126|79655705	0.5449920	0.0000000	0.0000000	0.0000000	6
chr17:80016721|80017823	0.7357392	0.2567267	0.9289464	0.1308132	1
chr17:80115624|80151706	0.6812400	0.1711511	0.2322366	0.6540660	5
chr17:80521230|80526077	0.2426032	0.2567267	0.4644732	0.1308132	2
chr17:80714041|80739597	0.0000000	0.0000000	0.6967098	0.0000000	2
chr17:80863812|80869665	0.0000000	0.0000000	0.6193750	0.0000000	2
chr17:80914571|81006446	0.0000000	0.0000000	1.3934197	0.0000000	2
chr17:953290|1003975	0.0000000	0.8557555	0.0000000	0.2616264	7
chr17:953290|1028702	0.4359936	0.0000000	0.6967098	0.3924396	1

circRNA_coordinates	m6A-CTRL-S1	m6A-CTRL-S2	m6A-BTZ-S1	m6A-BTZ-S2	cluster_group
chr17:953290|995090	0.0000000	0.0000000	0.2322366	0.3924396	4
chr17:970317|995090	0.0000000	0.0000000	0.4890903	0.0000000	2
chr17_ctg5_hap1:167529|168155	0.0000000	0.0000000	0.4644732	0.0000000	2
chr17_ctg5_hap1:167529|170575	0.4632432	0.0000000	0.0000000	0.1569758	6
chr18:12366964|12371690	0.2997456	0.0984975	0.6967098	0.0000000	1
chr18:12814202|12836890	0.0000000	0.0000000	0.6967098	0.0000000	2
chr18:12999420|13019205	0.0000000	0.0855756	2.0901295	0.5232528	2
chr18:12999420|13030607	0.3814944	0.3423022	0.9289464	1.0465055	4
chr18:12999420|13042333	0.0817488	0.8557555	0.2322366	0.3924396	7
chr18:13048858|13059311	0.0000000	0.5990289	0.0000000	0.6540660	5
chr18:13048858|13073184	0.3269952	0.1711511	0.4644732	0.2616264	1
chr18:13071038|13092526	0.5177424	0.0000000	0.0000000	0.0000000	6
chr18:13681604|13682104	2.0437199	0.6846044	0.0000000	0.0000000	6
chr18:19345733|19359646	0.5449920	0.4278778	1.6256563	0.6540660	2
chr18:19345733|19399607	0.9809855	1.6259355	0.9289464	0.7848792	7
chr18:21644104|21663045	0.1634976	0.0855756	0.2322366	0.2616264	4
chr18:21860806|21957499	0.8174880	0.0000000	1.3934197	1.3081319	1
chr18:21912906|21946930	0.0000000	0.0000000	0.4644732	0.5232528	4
chr18:22775124|22902151	0.0000000	0.5134533	1.6256563	0.7848792	2
chr18:22901972|22907412	0.3716300	0.0855756	0.2322366	0.5232528	4
chr18:22901972|22925311	0.5813157	0.0000000	0.0000000	0.3288644	6
chr18:2884962|2892484	1.0899839	0.5134533	0.0000000	0.3924396	6
chr18:2890559|2892484	1.9619711	1.5403599	6.7348617	1.9621979	2
chr18:29470698|29497630	0.0000000	0.0000000	0.4644732	0.0000000	2
chr18:29622142|29625695	0.0000000	0.0000000	0.0000000	0.5232528	5

circRNA_coordinates	m6A-CTRL-S1	m6A-CTRL-S2	m6A-BTZ-S1	m6A-BTZ-S2	cluster_group
chr18:29691717|29693823	0.2179968	0.1711511	0.0000000	0.3488788	5
chr18:32093479|32093901	0.4359936	0.0000000	0.0000000	0.0000000	6
chr18:33606863|33613800	0.4087440	0.2567267	0.0000000	0.0000000	7
chr18:34646853|34647508	0.0000000	0.0000000	0.4644732	0.1308132	2
chr18:34646853|34664195	0.1089984	0.5134533	0.0000000	0.0000000	7
chr18:39607407|39629569	0.5177424	0.0855756	0.1955432	0.3924396	1
chr18:44470543|44483598	0.6812400	0.0000000	2.5546027	1.1773187	2
chr18:44526020|44526886	0.3269952	0.0855756	0.0000000	0.2616264	6
chr18:45391430|45396935	0.0000000	0.0000000	0.9289464	0.0498398	2
chr18:45391430|45423180	4.9049277	1.0269066	3.2513126	2.2238243	1
chr18:45394694|45423180	0.2179968	0.0000000	0.0000000	0.2616264	5
chr18:46284290|46288060	0.6539904	0.0855756	0.2322366	1.0465055	4
chr18:47799354|47799703	0.2179968	0.0000000	0.0000000	0.2093011	5
chr18:48444480|48458730	0.5177424	0.0000000	0.0000000	0.0000000	6
chr18:48581151|48593557	0.0000000	0.0000000	0.0000000	0.7848792	5
chr18:51804073|51813781	0.0000000	0.0000000	0.2322366	0.3924396	4
chr18:54423814|54426184	3.3789502	0.6846044	4.8769688	2.3546375	1
chr18:54547175|54547396	2.4406375	0.0000000	0.0000000	0.0000000	6
chr18:55398646|55399255	0.0000000	0.0855756	0.0000000	1.1773187	5
chr18:56601665|56621031	0.4640334	0.0000000	0.0000000	0.0000000	6
chr18:61645533|61652479	0.5722416	0.0000000	0.0000000	0.0000000	6
chr18:74082483|74092259	1.5532271	0.1711511	0.0000000	0.0000000	6
chr18:74083422|74092259	0.0000000	0.0000000	2.0901295	0.0000000	2
chr18:74090810|74092259	2.0982191	0.5134533	0.6967098	2.8778902	5
chr18:74153201|74155167	7.0031468	2.4816910	5.1092055	3.9243958	1

circRNA_coordinates	m6A-CTRL-S1	m6A-CTRL-S2	m6A-BTZ-S1	m6A-BTZ-S2	cluster_group
chr18:74153201|74175097	1.0354847	0.3423022	0.6967098	0.1308132	6
chr18:74561482|74580819	0.0000000	0.0855756	0.0000000	0.5232528	5
chr18:74561482|74583781	0.0000000	0.0000000	1.5483215	0.0000000	2
chr18:74561482|74593468	0.0000000	0.3423022	0.2322366	0.0523253	3
chr18:74620276|74672811	0.0000000	0.0000000	0.4644732	0.0000000	2
chr18:74807324|74817242	1.6240761	0.0000000	0.0000000	0.0000000	6
chr18:77170403|77171501	2.5887119	1.1980577	0.4644732	0.9156923	6
chr18:77170403|77193738	0.2179968	0.0000000	0.0000000	0.3924396	5
chr18:77455225|77464917	2.5212146	0.0000000	0.0000000	0.0000000	6
chr18:77470346|77478016	0.2179968	0.0000000	0.0000000	0.2616264	5
chr18:77470346|77496521	0.0000000	0.2567267	0.4644732	0.2616264	2
chr18:77488907|77496521	1.0627343	0.5990289	2.7868393	0.0000000	2
chr18:9182380|9221997	1.2262319	1.1980577	0.9289464	0.3924396	3
chr18:9195549|9221997	0.0000000	0.6083566	0.9289464	0.1308132	3
chr18:9524592|9525849	0.3550078	0.0000000	0.0000000	0.2616264	6
chr18:9583115|9595100	0.0000000	0.4278778	0.0773348	0.0000000	7
chr19:10257027|10262523	0.0000000	0.5404096	0.0000000	0.0000000	7
chr19:10259560|10267185	0.0000000	0.0000000	0.4644732	0.0000000	2
chr19:10262074|10273424	1.4714783	0.6846044	0.9289464	0.5232528	6
chr19:10264969|10265732	0.1054559	0.0000000	8.0509466	0.0000000	2
chr19:10270334|10273424	2.9641568	0.0000000	0.0000000	0.0000000	6
chr19:10270334|10288043	0.4087440	0.5134533	0.0000000	0.1308132	7
chr19:10274002|10288043	0.4632432	0.1711511	0.2322366	0.0000000	6
chr19:1032391|1032695	0.3542448	0.0338023	0.8098091	0.7848792	4
chr19:10602253|10602938	0.6267408	0.0000000	0.0000000	0.0000000	6

circRNA_coordinates	m6A-CTRL-S1	m6A-CTRL-S2	m6A-BTZ-S1	m6A-BTZ-S2	cluster_group
chr19:1073148|1084345	0.0000000	0.0000000	0.6967098	0.0000000	2
chr19:10906048|10916643	0.4087440	0.0000000	0.0000000	0.0000000	6
chr19:1090907|1091906	0.0000000	0.0000000	0.2942438	0.1457259	2
chr19:10909249|10912940	0.2661468	0.6846044	0.2322366	0.0000000	7
chr19:11168934|11170863	0.0000000	0.0000000	0.0000000	0.6540660	5
chr19:11258809|11259769	0.0000000	0.5214974	0.0000000	0.0000000	7
chr19:11623871|11625036	0.7357392	0.0000000	0.4644732	0.5232528	1
chr19:1255061|1255401	1.8711482	0.0000000	0.0000000	0.1308132	6
chr19:12825635|12826553	0.3542448	0.1711511	0.0000000	0.6540660	5
chr19:12830162|12831692	0.5737675	0.0000000	0.4644732	0.0000000	1
chr19:13039156|13039661	0.6812400	0.1711511	1.6256563	0.2616264	1
chr19:14523363|14524036	0.0000000	0.0000000	0.4644732	0.0000000	2
chr19:15364963|15383944	0.0000000	0.5134533	0.0000000	0.0000000	7
chr19:15375215|15383944	0.0000000	0.1711511	0.4644732	0.0000000	2
chr19:15379716|15383944	0.0000000	0.0855756	0.4644732	0.0000000	2
chr19:15480957|15484876	0.0000000	0.0000000	0.6967098	0.0000000	2
chr19:16192723|16199930	0.0000000	0.0000000	3.2513126	0.0000000	2
chr19:16264019|16265181	0.0000000	0.0000000	0.4644732	0.0000000	2
chr19:16790795|16793319	0.1634976	0.0000000	0.1955432	0.0523253	1
chr19:17212470|17213367	8.4201259	2.3105399	6.0381519	7.0639124	1
chr19:17286472|17291849	0.0000000	0.0000000	0.5963836	0.0000000	2
chr19:17378337|17379581	0.4632432	0.0000000	0.0000000	0.1308132	6
chr19:17533268|17534285	0.0000000	0.0000000	0.6967098	0.0000000	2
chr19:17533268|17534286	0.0000000	0.0855756	0.0000000	0.5232528	5
chr19:17678215|17679522	0.0000000	0.0000000	0.4746916	0.0000000	2

circRNA_coordinates	m6A-CTRL-S1	m6A-CTRL-S2	m6A-BTZ-S1	m6A-BTZ-S2	cluster_group
chr19:17889694|17892015	0.4087440	0.0000000	0.0000000	0.0000000	6
chr19:18258253|18258434	0.2724960	0.0000000	0.2322366	0.0000000	1
chr19:18305897|18306780	0.5177424	0.0000000	0.0000000	0.0000000	6
chr19:18648411|18649246	1.6894751	0.2567267	0.6967098	0.9156923	1
chr19:18650181|18650530	0.4485556	0.0000000	0.0000000	0.0419910	6
chr19:18853720|18864395	0.4689383	0.0000000	0.0000000	0.0000000	6
chr19:18968343|18971111	0.5449920	0.0000000	0.0000000	0.0000000	6
chr19:19153520|19166205	0.0000000	0.0000000	0.6967098	0.0000000	2
chr19:19455736|19456088	4.1219378	0.0000000	0.0000000	0.0000000	6
chr19:19546940|19576423	0.2179968	0.0000000	0.0000000	0.5232528	5
chr19:19576149|19576423	0.0000000	0.5134533	1.6256563	2.8778902	4
chr19:19576154|19576423	0.2179968	0.2567267	0.4644732	0.5232528	4
chr19:19603115|19603521	0.8568636	0.0000000	0.8971300	0.0000000	1
chr19:19750894|19751380	0.9537359	0.5990289	0.6967098	0.6540660	6
chr19:2076810|2078678	0.0000000	1.6259355	1.1611831	1.1773187	5
chr19:2078139|2078678	0.0000000	0.4278778	0.0000000	0.0000000	7
chr19:2109228|2109871	0.2452464	0.1711511	0.3910865	0.5232528	4
chr19:21216262|21216990	1.7984735	0.9413311	0.6967098	0.3924396	6
chr19:2211842|2213515	0.5994912	0.0000000	0.0000000	0.0000000	6
chr19:2213538|2214595	1.5830927	0.0000000	0.0000000	0.0000000	6
chr19:2214596|2216257	0.4359936	0.0000000	0.0000000	0.0000000	6
chr19:2246881|2247661	0.0000000	0.0000000	0.0000000	1.1835978	5
chr19:23844916|23845960	0.5177424	0.0000000	0.0000000	0.0000000	6
chr19:33116780|33119755	0.0000000	0.0000000	0.6709316	0.0000000	2
chr19:33390763|33392318	0.0000000	0.2567267	0.2322366	0.0000000	3

circRNA_coordinates	m6A-CTRL-S1	m6A-CTRL-S2	m6A-BTZ-S1	m6A-BTZ-S2	cluster_group
chr19:33604673|33617639	0.0605486	0.0000000	0.9289464	0.0000000	2
chr19:3381710|3382241	1.0627343	0.0000000	0.9289464	0.1308132	1
chr19:3381710|3435205	0.0000000	0.0000000	0.0000000	1.1773187	5
chr19:3381710|3456633	0.4359936	0.0000000	0.0000000	0.0000000	6
chr19:3425104|3449137	0.0000000	0.0000000	0.9289464	0.0000000	2
chr19:3449012|3452664	0.0000000	0.0000000	0.6767375	0.0000000	2
chr19:34921481|34925873	0.1907472	0.0855756	0.0000000	0.3924396	5
chr19:34921481|34929671	0.3269952	0.0898543	0.3095714	0.1308132	1
chr19:34921481|34955036	0.2724960	0.0000000	0.2322366	0.0498398	1
chr19:34921481|34957919	0.3269952	0.0000000	0.0000000	0.7848792	5
chr19:34934749|34936026	0.0000000	0.4519245	0.0000000	0.0000000	7
chr19:3494131|3496538	0.0000000	0.0000000	0.6967098	0.0000000	2
chr19:34942886|34955036	0.9809855	0.0000000	0.0000000	0.0000000	6
chr19:35762045|35769580	0.5449920	0.0000000	0.0000000	0.2616264	6
chr19:3647408|3648622	1.2654986	0.0000000	0.0000000	0.0000000	6
chr19:3651824|3653587	0.0000000	0.0855756	0.0000000	0.3924396	5
chr19:37916770|37917280	0.5722416	0.0000000	0.0000000	0.0000000	6
chr19:38597177|38610522	0.7629888	0.1711511	0.2322366	0.3924396	6
chr19:38631824|38633350	0.3144604	0.0855756	0.0000000	0.1811763	6
chr19:39191240|39196771	0.5056163	0.0000000	0.0000000	0.0000000	6
chr19:39191240|39200116	0.0000000	0.0000000	1.5111636	0.0000000	2
chr19:39943996|39950600	0.5994912	0.0000000	0.0000000	0.0000000	6
chr19:4048273|4053968	0.0000000	0.3423022	1.3934197	0.0000000	2
chr19:40747845|40748594	0.4119049	0.1530091	0.0000000	0.0000000	6
chr19:41088257|41089623	0.1362480	0.0855756	0.0000000	0.2616264	5

circRNA_coordinates	m6A-CTRL-S1	m6A-CTRL-S2	m6A-BTZ-S1	m6A-BTZ-S2	cluster_group
chr19:41088373|41089279	0.0000000	0.1711511	0.6967098	0.0000000	2
chr19:41088373|41089281	0.0000000	1.8826621	0.0000000	0.0000000	7
chr19:4117417|4117627	0.0000000	0.0000000	0.4644732	0.0000000	2
chr19:41231245|41239269	0.0000000	0.0000000	0.0000000	0.5232528	5
chr19:41774128|41787180	0.7629888	0.0000000	0.0000000	0.6540660	6
chr19:41774128|41800591	0.0000000	0.0855756	0.6967098	0.0000000	2
chr19:42392350|42392790	0.4949617	0.0000000	0.0000000	0.0000000	6
chr19:4251169|4254327	0.0000000	0.0000000	0.0000000	0.7848792	5
chr19:4405909|4409756	5.2591725	1.1980577	2.3223661	1.0465055	6
chr19:4405909|4418073	0.3269952	0.2567267	0.4644732	0.0000000	3
chr19:4408900|4409756	1.1717327	0.8557555	0.0000000	0.9156923	7
chr19:44169634|44171732	0.5449920	0.0855756	0.0000000	0.0000000	6
chr19:4418017|4433536	0.0000000	0.2567267	0.0000000	0.3924396	5
chr19:4418017|4442338	0.1089984	0.0000000	0.4644732	0.0000000	2
chr19:44251594|44254899	0.0000000	0.0000000	0.4644732	0.0000000	2
chr19:4430546|4432204	0.3764532	0.0000000	0.0000000	0.1308132	6
chr19:4430546|4433536	1.3079807	0.0000000	0.0000000	0.5232528	6
chr19:45781181|45783992	0.2179968	0.1711511	0.6063698	0.1308132	2
chr19:46191006|46191644	0.6026249	0.0000000	0.0000000	0.0000000	6
chr19:46334642|46334846	0.0000000	0.0000000	3.5436985	0.0000000	2
chr19:46355831|46357432	0.4359936	0.0000000	0.0000000	0.0000000	6
chr19:4700228|4705982	0.0000000	0.0000000	0.4644732	0.0000000	2
chr19:47192977|47193856	0.0000000	0.0000000	0.4644732	0.0000000	2
chr19:47421745|47425613	0.2724960	0.0855756	0.0000000	0.2616264	5
chr19:47421745|47440665	0.0000000	0.3423022	0.0000000	0.7848792	5

circRNA_coordinates	m6A-CTRL-S1	m6A-CTRL-S2	m6A-BTZ-S1	m6A-BTZ-S2	cluster_group
chr19:47539388|47542259	0.0000000	0.0000000	0.4644732	0.0000000	2
chr19:47584770|47585552	0.0000000	0.0000000	0.9844510	0.0000000	2
chr19:47767860|47768203	1.9347215	1.2836333	0.9289464	2.7470770	5
chr19:47856011|47856992	0.3542448	0.1711511	0.6967098	0.2616264	1
chr19:47856011|47865950	1.6077263	0.2567267	0.6967098	1.7005715	1
chr19:47995403|47996217	0.1362480	0.0855756	0.4644732	0.6540660	4
chr19:48173700|48184533	0.0000000	0.0000000	0.4644732	0.0000000	2
chr19:48185233|48198731	0.3814944	0.0000000	0.4644732	0.0000000	1
chr19:48630534|48631277	0.4632432	0.0000000	0.0000000	0.0000000	6
chr19:48630534|48634428	0.0000000	0.0000000	0.5935968	0.0000000	2
chr19:48653020|48654596	0.4632432	0.0000000	0.6967098	0.0000000	1
chr19:48653020|48654642	0.0000000	1.3775952	0.0000000	0.0000000	7
chr19:48734917|48745901	0.0000000	0.4278778	0.0000000	0.0000000	7
chr19:48735801|48737659	0.2724960	0.0000000	0.0000000	0.3924396	5
chr19:4929234|4932968	0.4087440	0.0000000	0.0000000	0.0000000	6
chr19:4954362|4956824	0.0000000	0.0000000	0.9289464	0.0000000	2
chr19:49593548|49593777	0.2555740	0.0000000	0.2322366	0.0000000	1
chr19:50102529|50105175	0.3269952	0.0000000	1.8578929	0.3924396	2
chr19:5016268|5041262	0.0000000	0.0000000	0.4256897	0.0000000	2
chr19:50188295|50188745	0.5032728	0.0000000	0.0000000	0.0000000	6
chr19:5047487|5082515	0.0000000	0.1711511	2.4254792	0.1084441	2
chr19:50823674|50823849	0.0000000	0.0000000	0.4644732	0.0000000	2
chr19:50880844|50883136	0.0000000	0.0000000	1.2385178	0.0000000	2
chr19:50902105|50902741	0.4632432	0.0000000	0.0000000	0.5232528	5
chr19:50902108|50902741	1.3624799	1.3692088	1.0706108	0.5232528	3

circRNA_coordinates	m6A-CTRL-S1	m6A-CTRL-S2	m6A-BTZ-S1	m6A-BTZ-S2	cluster_group
chr19:50909775|50910208	0.0000000	0.0000000	0.6967098	0.0000000	2
chr19:51169449|51172542	0.1907472	0.1711511	0.0000000	0.1308132	7
chr19:52785364|52786666	1.6572116	0.0000000	0.0000000	0.0000000	6
chr19:54308534|54318242	0.0817488	0.0000000	0.9289464	0.6540660	4
chr19:54625974|54626805	0.0000000	0.2567267	0.0000000	0.3924396	5
chr19:54646665|54657577	0.3814944	0.0855756	0.0000000	0.0000000	6
chr19:54705478|54710120	0.0000000	0.3423022	0.0000000	0.1308132	7
chr19:55855366|55857562	0.0000000	0.1711511	0.0000000	0.5232528	5
chr19:5591759|5595508	0.4632432	0.0855756	0.0000000	0.6540660	5
chr19:5591759|5600271	0.6267408	0.0000000	0.0000000	0.3924396	6
chr19:5591759|5613538	0.0000000	0.0000000	0.9289464	0.0000000	2
chr19:5604594|5604947	0.3814944	0.2567267	0.6967098	0.6540660	4
chr19:5610006|5613538	0.6539904	0.0000000	0.0000000	0.0000000	6
chr19:56171588|56171843	0.0000000	0.0000000	0.4644732	0.0000000	2
chr19:5641605|5641957	0.3269952	0.0855756	0.1955432	0.0000000	6
chr19:5645348|5649986	0.5177424	0.0000000	0.0000000	0.0000000	6
chr19:5648055|5648963	0.4359936	0.0000000	0.0000000	0.0000000	6
chr19:5705783|5713264	0.2724960	0.0000000	0.0000000	0.2616264	5
chr19:5711782|5714282	0.3542448	0.0000000	0.0000000	0.3924396	5
chr19:5787390|5787599	1.1234737	0.0898543	0.3910865	0.0000000	6
chr19:58805464|58806947	0.4904928	0.0000000	0.0000000	0.0000000	6
chr19:6826623|6829929	0.0000000	0.0000000	0.6967098	0.0000000	2
chr19:7163043|7174742	0.0000000	0.0000000	2.7868393	0.0000000	2
chr19:7184327|7184648	0.7586561	0.0000000	0.0000000	0.0000000	6
chr19:7267356|7267907	0.4904928	0.2567267	0.4644732	0.6540660	4

circRNA_coordinates	m6A-CTRL-S1	m6A-CTRL-S2	m6A-BTZ-S1	m6A-BTZ-S2	cluster_group
chr19:7996023|8000030	0.4087440	0.0000000	0.0000000	0.0000000	6
chr19:829707|830687	0.0000000	0.0000000	2.3739226	0.0000000	2
chr19:843461|844034	0.5722416	0.2759812	0.0000000	0.0000000	7
chr19:844035|846123	1.2807311	0.0000000	0.0000000	0.0000000	6
chr19:8520289|8528570	0.0000000	0.1278499	0.2322366	0.1308132	2
chr19:8531273|8532404	0.4632432	0.0000000	0.0000000	0.0000000	6
chr19:855795|855935	0.0000000	0.1228865	0.2322366	0.2616264	5
chr19:8606889|8609180	0.0000000	0.0000000	0.4644732	0.0000000	2
chr19:8612920|8613201	0.0000000	0.0000000	3.8565212	0.0000000	2
chr19:8615605|8616623	0.3542448	0.3423022	0.2322366	1.0465055	5
chr19:8619361|8620680	0.0000000	0.0000000	0.4644732	0.0000000	2
chr1:10463128|10473308	0.3269952	0.0000000	0.1755709	0.0567729	6
chr1:10527247|10529395	1.5001449	0.0000000	0.4851423	0.0000000	6
chr1:108247172|108247683	0.0000000	0.0000000	0.9289464	0.0000000	2
chr1:11150611|11151654	0.0000000	0.0000000	0.4644732	0.0000000	2
chr1:113140593|113159517	0.0000000	0.0000000	0.4888581	0.0000000	2
chr1:113189832|113202401	0.5177424	0.2567267	0.0000000	0.0000000	7
chr1:113196220|113202401	0.3480046	0.0000000	0.4644732	0.0000000	1
chr1:114267381|114301355	0.4087440	0.0000000	0.0000000	0.0000000	6
chr1:114377532|114397671	0.6812400	0.0000000	0.2322366	0.0000000	6
chr1:114483004|114484081	0.3542448	0.3423022	0.0000000	0.0000000	7
chr1:1158624|1159348	1.3865686	0.4966805	0.2322366	0.3924396	6
chr1:117944808|117948267	0.1040935	1.1124822	0.6967098	1.0465055	5
chr1:117944808|117957453	1.7712239	1.7115110	0.2322366	4.1860222	5
chr1:117944808|117963271	2.3434655	3.6797487	3.4835492	3.9243958	5

circRNA_coordinates	m6A-CTRL-S1	m6A-CTRL-S2	m6A-BTZ-S1	m6A-BTZ-S2	cluster_group
chr1:117944808|117984947	0.8174880	0.6846044	0.0000000	1.1773187	5
chr1:117944808|118009049	0.0000000	0.3423022	0.4644732	0.5232528	5
chr1:121115832|121116815	0.5177424	0.0000000	0.0000000	0.0000000	6
chr1:12335882|12338095	0.2179968	0.3423022	0.0000000	0.6540660	5
chr1:14099573|14109326	0.7084896	0.9413311	0.0000000	0.1308132	7
chr1:14104913|14109326	1.1989823	0.5990289	0.2322366	0.0000000	6
chr1:145227041|145281704	0.1634976	0.5134533	0.0000000	0.0000000	7
chr1:146661756|146696658	0.0000000	0.0855756	0.6967098	0.0000000	2
chr1:149239868|149255307	2.5887119	0.0000000	0.0000000	0.0000000	6
chr1:150305155|150312953	0.2179968	0.0000000	0.0000000	0.2616264	5
chr1:150416727|150418877	0.4904928	0.0000000	0.0000000	0.0000000	6
chr1:150464887|150469384	0.4359936	0.2086332	0.1955432	0.0000000	6
chr1:150464887|150470223	0.5449920	0.0000000	0.0000000	0.0000000	6
chr1:150923841|150935195	0.1089984	0.7701800	0.9289464	0.0000000	3
chr1:150933039|150934634	1.0627343	0.5134533	0.2322366	0.0000000	6
chr1:151089868|151090615	0.2452464	0.0855756	0.2322366	0.2616264	1
chr1:151090616|151092176	0.6327629	0.0000000	0.0000000	0.0000000	6
chr1:151139410|151139890	0.0017985	0.0000000	0.2322366	0.3139517	4
chr1:151196721|151219441	0.0000000	0.0000000	0.6967098	0.0000000	2
chr1:151288049|151288985	0.9809855	0.0000000	0.0000000	0.0000000	6
chr1:151395873|151403317	0.0000000	0.0000000	0.4644732	0.0000000	2
chr1:154197560|154218851	0.0000000	0.1711511	0.4644732	0.0000000	2
chr1:154207067|154207767	0.8447376	0.4278778	0.4644732	0.2616264	6
chr1:154941955|154942507	0.0000000	0.0000000	0.5866297	0.0000000	2
chr1:155180419|155181891	0.0000000	0.4278778	0.4644732	0.1308132	3

circRNA_coordinates	m6A-CTRL-S1	m6A-CTRL-S2	m6A-BTZ-S1	m6A-BTZ-S2	cluster_group
chr1:155327107|155327540	0.1362480	0.0573356	0.0000000	0.3924396	5
chr1:155340295|155408859	0.0000000	0.0000000	0.4644732	0.0000000	2
chr1:155365250|155429689	0.3814944	1.5403599	0.6967098	0.6540660	7
chr1:155385535|155408859	0.5177424	0.2567267	0.4644732	0.2616264	1
chr1:155385535|155429689	1.1172335	0.6846044	2.0901295	0.0000000	3
chr1:155408118|155408859	1.6622255	1.4547844	1.3934197	2.6162639	5
chr1:155408118|155429689	14.7692824	9.1565840	16.0243262	14.2586380	1
chr1:155490891|155491409	0.6539904	0.0000000	0.0000000	0.1308132	6
chr1:155640111|155649303	0.7357392	0.2567267	0.0000000	0.0000000	6
chr1:155644801|155649303	0.8447376	0.1711511	1.8063364	0.2616264	1
chr1:155646339|155649303	1.5804767	0.0855756	0.4644732	0.9156923	1
chr1:155733103|155747611	0.0000000	0.0000000	0.4644732	0.0000000	2
chr1:155823067|155823597	1.9347215	1.9682377	3.0190760	1.5697583	2
chr1:155896467|155896947	0.0000000	0.1711511	0.3910865	0.0000000	2
chr1:156169626|156170263	0.0000000	0.0000000	1.1611831	0.0000000	2
chr1:156303338|156304709	0.0000000	0.0000000	0.4644732	0.0000000	2
chr1:156445052|156446803	0.0000000	0.0000000	0.0000000	0.5232528	5
chr1:156568935|156571200	0.0000000	0.0000000	0.4644732	0.0000000	2
chr1:156713444|156715165	0.0000000	0.0000000	0.6967098	0.0000000	2
chr1:156752074|156756966	0.7084896	0.0000000	0.0000000	0.3924396	6
chr1:15855583|15863309	0.5449920	0.0000000	0.0000000	0.0000000	6
chr1:15860732|15874923	0.1089984	0.0855756	0.0000000	0.5232528	5
chr1:1586823|1588948	0.0000000	0.0000000	0.7224881	0.0000000	2
chr1:15964802|15970145	0.2724960	0.0855756	0.0977716	0.2616264	5
chr1:160195381|160213824	0.0000000	0.0000000	0.9289464	0.0000000	2

circRNA_coordinates	m6A-CTRL-S1	m6A-CTRL-S2	m6A-BTZ-S1	m6A-BTZ-S2	cluster_group
chr1:160206925|160210160	0.0000000	0.0000000	0.4644732	0.0000000	2
chr1:160206925|160213824	0.3814944	0.0000000	0.0000000	0.3924396	5
chr1:160268635|160295442	0.0000000	0.0000000	0.4644732	0.0000000	2
chr1:16046229|16047883	2.6756381	0.0000000	0.0000000	0.0000000	6
chr1:16199311|16203173	0.0000000	0.0000000	0.0000000	0.5232528	5
chr1:16528277|16529069	0.6812400	0.0000000	1.1611831	0.0000000	1
chr1:165859441|165860559	0.2724960	0.0000000	0.2933148	0.0000000	1
chr1:171492360|171496983	0.2109119	0.5562411	0.0000000	0.0000000	7
chr1:171537386|171544267	0.2155171	0.0000000	0.4902515	0.0000000	1
chr1:171548493|171556890	0.3814944	0.0000000	0.0000000	0.0355812	6
chr1:1724684|1756938	0.0000000	0.0000000	0.0000000	3.1270894	5
chr1:1735858|1770677	0.2179968	0.2567267	0.0000000	0.6540660	5
chr1:1738007|1747194	0.0000000	0.0000000	0.0000000	0.5232528	5
chr1:173961893|173962273	0.4087440	0.0000000	0.0000000	0.1308132	6
chr1:1747195|1756938	0.6903141	0.2567267	0.9289464	0.6540660	1
chr1:1747195|1770677	0.1907472	0.1028618	1.7601213	0.6540660	2
chr1:1756836|1770677	0.0670885	0.0000000	0.4695824	0.0000000	2
chr1:176132005|176153828	0.3314369	0.0000000	0.0977716	0.0000000	6
chr1:179012850|179023773	0.3542448	0.0000000	0.6967098	0.0000000	1
chr1:179825193|179834989	0.0000000	0.0000000	1.1611831	0.0000000	2
chr1:179955304|179966310	0.0000000	0.0000000	0.0000000	0.4671339	5
chr1:179955304|179972422	0.0000000	0.0000000	0.9289464	0.0000000	2
chr1:179955304|179975702	0.0000000	0.5134533	0.0000000	0.0000000	7
chr1:179965688|179966310	0.1089984	0.0855756	0.6967098	0.0000000	2
chr1:180953813|180962561	0.4087440	0.1999045	0.0977716	0.1569758	6

circRNA_coordinates	m6A-CTRL-S1	m6A-CTRL-S2	m6A-BTZ-S1	m6A-BTZ-S2	cluster_group
chr1:184675777|184681011	0.7231498	0.0000000	0.0000000	0.0000000	6
chr1:186862143|186880521	0.5062158	0.0855756	0.4888581	0.0000000	1
chr1:193110980|193121574	0.3027703	0.0000000	0.0000000	0.1046506	6
chr1:19644094|19644335	0.0000000	0.0000000	0.7078572	0.0000000	2
chr1:203816312|203816815	0.5722416	0.0000000	0.0000000	0.6540660	5
chr1:203830842|203831308	0.0000000	0.0000000	0.4888581	0.0000000	2
chr1:204082043|204083560	0.0000000	0.0000000	0.7224881	0.0000000	2
chr1:204437998|204439014	0.7084896	0.0000000	0.0000000	0.0000000	6
chr1:205585606|205593019	2.7522095	2.7384176	2.7868393	1.7005715	3
chr1:21083659|21100103	0.8992368	0.4403718	0.4644732	0.6540660	6
chr1:21083659|21107033	0.0000000	0.5909848	0.0000000	0.0000000	7
chr1:21167349|21191755	0.0000000	0.1711511	0.6967098	0.2616264	2
chr1:21180053|21191205	0.4037573	0.0000000	0.0000000	0.0000000	6
chr1:21180053|21231464	0.0000000	0.0000000	0.6967098	0.0000000	2
chr1:21212694|21231464	0.4632432	0.0000000	0.0000000	0.0000000	6
chr1:21220010|21268823	0.0817488	0.0000000	0.4644732	0.3924396	4
chr1:21231376|21268823	0.6267408	0.5990289	0.0000000	0.9156923	5
chr1:21267984|21268823	0.8992368	0.0000000	0.9289464	0.0000000	1
chr1:21267984|21276601	0.0000000	0.0840352	0.6967098	0.2616264	2
chr1:21276496|21299592	1.4252085	0.0000000	0.0000000	0.0000000	6
chr1:215759838|215768813	0.2997456	0.0000000	0.2322366	0.0000000	1
chr1:220170325|220195860	0.4359936	0.0000000	0.0000000	0.0000000	6
chr1:222897434|222898897	0.0000000	0.4345527	0.0000000	0.0000000	7
chr1:2234417|2236024	0.0000000	0.1339257	2.9932977	0.2616264	2
chr1:224599129|224601037	0.0000000	0.0000000	0.2322366	0.2093011	4

circRNA_coordinates	m6A-CTRL-S1	m6A-CTRL-S2	m6A-BTZ-S1	m6A-BTZ-S2	cluster_group
chr1:227149078|227153438	0.0817488	0.0855756	0.9289464	0.9718112	4
chr1:22816373|22818026	4.2509374	2.8239932	2.3223661	3.2703298	6
chr1:231829572|231837770	0.0000000	0.0000000	0.6967098	0.0000000	2
chr1:232649603|232651354	0.4632432	0.0000000	0.0000000	0.2616264	6
chr1:233482188|233498039	0.0000000	0.0000000	0.4644732	0.0000000	2
chr1:23356962|23377013	0.3542448	0.0855756	0.6967098	0.0000000	1
chr1:235299420|235301191	0.0000000	0.0000000	0.9289464	0.0000000	2
chr1:235357328|235377341	0.8447376	0.0855756	0.0977716	0.5232528	6
chr1:235954863|235954998	0.0000000	0.0000000	0.4644732	0.0000000	2
chr1:235993526|235996967	0.2997456	0.1682415	0.9289464	0.3924396	2
chr1:236966728|236979843	0.0000000	0.0000000	0.9289464	0.0000000	2
chr1:242042051|242048809	0.0000000	0.1711511	0.6967098	0.0000000	2
chr1:244715624|244724447	0.3542448	0.0000000	0.0000000	0.3924396	5
chr1:245008679|245009905	0.0000000	0.0000000	0.6967098	0.0000000	2
chr1:245165423|245180628	0.0903869	0.2567267	0.0000000	0.5232528	5
chr1:245165423|245222760	0.1634976	0.0000000	0.2322366	0.1495195	1
chr1:246021798|246093239	0.0544992	0.0000000	0.4644732	0.0000000	2
chr1:246754814|246755243	0.4087440	0.0000000	0.4644732	0.0000000	1
chr1:247322308|247323115	0.0000000	0.3423022	0.2322366	0.3844600	5
chr1:24987210|24996806	0.7084896	0.0000000	0.4644732	0.0000000	6
chr1:24993306|24996806	0.6267408	0.0855756	1.1611831	0.1308132	1
chr1:25551493|25554726	0.4359936	0.0000000	0.0000000	0.0000000	6
chr1:26566253|26596105	0.0000000	0.0000000	0.4644732	0.0000000	2
chr1:26581662|26582356	0.3542448	0.0000000	0.0000000	0.5232528	5
chr1:26584088|26586293	0.0000000	0.0000000	1.8578929	0.0000000	2

circRNA_coordinates	m6A-CTRL-S1	m6A-CTRL-S2	m6A-BTZ-S1	m6A-BTZ-S2	cluster_group
chr1:26594974|26596105	0.2452464	0.9705123	0.7740446	0.1308132	3
chr1:26873475|26873654	0.1089984	0.1711511	0.2322366	0.0000000	3
chr1:27056142|27059283	0.4632432	1.1124822	0.4644732	0.7848792	7
chr1:27267948|27268309	0.2724960	0.3423022	1.1611831	0.6540660	2
chr1:27269151|27269556	2.3162159	0.0000000	1.1611831	1.4389451	1
chr1:28362055|28384605	0.4087440	0.1711511	0.0000000	0.9156923	5
chr1:28800066|28800663	0.7357392	0.0855756	0.4644732	0.7848792	1
chr1:28800066|28802803	0.7629888	0.5990289	1.6256563	1.7005715	4
chr1:28800066|28807116	0.2724960	0.0000000	0.6967098	0.0000000	1
chr1:28907072|28907741	0.0000000	0.1711511	0.1031131	0.1308132	5
chr1:29068915|29070498	3.1337038	2.6528421	5.5736787	3.4011430	2
chr1:29313943|29314417	0.7629888	0.0000000	0.4644732	1.4389451	4
chr1:29313943|29323831	0.0000000	0.5990289	0.0000000	0.5232528	5
chr1:29313943|29379824	0.6267408	0.0000000	0.0000000	0.0000000	6
chr1:29481208|29481422	0.0000000	0.5736129	0.6967098	0.0000000	3
chr1:31452909|31468067	0.5722416	0.0000000	0.0000000	0.0000000	6
chr1:31465237|31468067	0.4632432	0.3423022	0.6967098	0.0435608	3
chr1:31465237|31479949	0.0000000	0.4278778	0.2322366	0.0000000	3
chr1:31478700|31479949	0.4331324	0.0000000	0.3483549	0.0000000	1
chr1:31528160|31532424	0.5722416	0.0855756	2.1674643	0.6540660	1
chr1:31810022|31811895	0.0000000	0.4171808	0.0000000	0.0000000	7
chr1:31810022|31821821	0.0000000	0.0000000	0.4644732	0.0000000	2
chr1:32381496|32385259	0.0817488	0.4278778	0.0000000	0.0000000	7
chr1:32620189|32625132	0.0000000	0.0000000	0.0000000	0.6540660	5
chr1:32790080|32793278	0.0000000	0.0000000	0.9289464	0.0000000	2

circRNA_coordinates	m6A-CTRL-S1	m6A-CTRL-S2	m6A-BTZ-S1	m6A-BTZ-S2	cluster_group
chr1:33244983|33256855	0.0000000	0.0000000	0.0000000	0.5232528	5
chr1:33413823|33415375	0.0000000	0.0000000	0.5866297	0.0000000	2
chr1:35457835|35496314	0.0000000	0.0000000	0.4644732	0.0000000	2
chr1:35824526|35827390	2.8612078	1.7970866	1.8578929	0.7848792	6
chr1:35846860|35854683	0.0000000	0.0000000	1.1611831	0.0000000	2
chr1:35846860|35855699	0.7084896	0.3423022	0.0000000	0.3924396	6
chr1:35851043|35854683	0.6439353	0.0000000	0.0000000	0.0000000	6
chr1:35857813|35865177	0.0000000	0.3423022	0.4644732	0.0000000	3
chr1:35859196|35879684	0.0000000	0.0000000	0.4644732	0.0000000	2
chr1:36028235|36028766	3.2541743	0.0000000	0.0000000	0.0000000	6
chr1:36809896|36814300	0.1634976	0.0000000	0.0000000	0.2616264	5
chr1:39851106|39854330	0.0000000	0.0855756	0.4644732	0.5232528	4
chr1:41471767|41473088	0.0000000	0.5134533	0.0000000	0.0000000	7
chr1:41536267|41541123	0.7629888	0.2567267	0.4644732	0.6540660	1
chr1:42693554|42744343	0.4087440	0.0855756	0.0000000	0.1308132	6
chr1:42730786|42744343	0.0000000	0.4278778	0.0977716	1.0465055	5
chr1:42730786|42776781	0.0000000	0.0000000	0.6967098	0.3924396	2
chr1:44137176|44149475	0.1907472	0.0000000	0.4644732	0.0000000	1
chr1:44137176|44156720	0.5994912	0.1901489	0.0000000	0.0000000	6
chr1:44157983|44159642	0.0000000	0.2567267	0.0000000	0.5232528	5
chr1:44303891|44366505	0.1907472	0.1711511	0.2322366	0.1308132	3
chr1:44303891|44386600	0.0000000	0.0000000	0.5970803	0.0000000	2
chr1:44386076|44386600	1.1246727	0.1005513	0.0000000	1.1337579	5
chr1:44877653|44878394	21.9631764	10.5257928	24.3848442	33.6189905	4
chr1:45223228|45226374	0.0000000	0.0000000	0.0000000	0.5232528	5

circRNA_coordinates	m6A-CTRL-S1	m6A-CTRL-S2	m6A-BTZ-S1	m6A-BTZ-S2	cluster_group
chr1:47745913|47748131	0.9537359	0.5990289	2.5546027	0.5232528	2
chr1:47761437|47767420	0.6539904	0.0855756	0.0000000	0.0000000	6
chr1:51001041|51061888	0.2179968	0.0855756	0.9289464	0.9156923	4
chr1:51005264|51061888	0.0000000	0.0000000	0.6967098	0.0000000	2
chr1:51032749|51061888	0.5994912	0.0000000	0.0000000	0.1308132	6
chr1:52269486|52275073	0.0000000	0.0000000	0.4644732	0.0000000	2
chr1:58971732|59004982	0.6539904	0.7701800	0.0000000	0.6540660	7
chr1:59139245|59155921	0.4087440	0.0000000	0.0000000	0.0000000	6
chr1:59147457|59150923	0.1907472	0.0855756	0.2322366	0.2616264	4
chr1:6142335|6145242	0.3269952	0.2567267	0.4644732	0.0000000	3
chr1:6252990|6257816	0.0000000	0.0000000	0.8655459	0.0000000	2
chr1:63269390|63307218	0.0000000	0.1711511	0.0000000	0.2616264	5
chr1:63282246|63307218	0.4087440	0.0000000	0.0000000	0.0000000	6
chr1:63944435|63974241	0.1362480	0.0855756	0.0000000	0.5232528	5
chr1:65316487|65335157	0.4087440	0.0000000	0.0000000	0.0000000	6
chr1:6679863|6680397	0.4087440	0.0000000	0.2322366	0.0000000	6
chr1:6727769|6741097	0.0000000	0.0855756	0.0000000	0.3924396	5
chr1:78097520|78107340	0.0000000	0.2567267	0.0000000	0.3924396	5
chr1:78097535|78107340	0.0000000	0.4278778	0.0000000	1.0465055	5
chr1:78183552|78191447	0.1657321	0.0000000	0.9289464	0.0000000	2
chr1:7837220|7838229	0.0847735	0.3423022	0.2064583	0.1308132	3
chr1:78478735|78479303	0.0000000	0.0000000	0.4644732	0.3924396	4
chr1:85331068|85331821	0.2724960	0.0000000	0.0000000	0.1308132	6
chr1:8674620|8716500	0.6539904	0.0000000	0.0000000	0.0000000	6
chr1:8716032|8716500	0.0000000	0.3423022	0.0000000	0.6540660	5

circRNA_coordinates	m6A-CTRL-S1	m6A-CTRL-S2	m6A-BTZ-S1	m6A-BTZ-S2	cluster_group
chr1:89206671|89251896	0.1210972	0.0000000	0.0000000	0.5232528	5
chr1:89225905|89237562	0.8195589	0.0000000	0.0000000	0.0000000	6
chr1:92754471|92757094	0.0000000	0.0000000	0.6967098	0.0000000	2
chr1:93657557|93672955	0.0000000	0.0000000	0.4644732	0.0000000	2
chr1:93676359|93682303	0.2724960	0.3423022	0.0000000	0.0000000	7
chr20:10536879|10541468	0.0000000	0.0173718	0.0000000	0.6540660	5
chr20:16351231|16362392	0.0000000	0.0299514	0.0000000	0.3924396	5
chr20:16506737|16509085	0.5449920	0.0855756	0.0000000	1.0465055	5
chr20:17602627|17605220	0.2724960	0.1520678	0.0000000	0.0000000	7
chr20:17928130|17932239	0.4087440	0.0000000	0.4644732	0.0000000	1
chr20:17928130|17937681	0.0000000	0.5134533	0.0000000	1.0465055	5
chr20:17934640|17936119	0.0000000	0.0000000	0.4644732	0.0000000	2
chr20:17949124|17949342	0.0000000	0.0000000	0.6967098	0.0000000	2
chr20:18122852|18123563	3.1882030	0.4278778	0.0000000	0.2616264	6
chr20:18122852|18131586	0.4087440	0.0000000	0.0000000	0.2616264	6
chr20:1905524|1908500	0.5177424	0.0000000	0.0000000	0.0000000	6
chr20:20493157|20501744	0.0544992	0.0000000	0.4644732	0.0000000	2
chr20:2097312|2098061	0.7084896	0.5134533	0.0000000	0.2616264	7
chr20:21306917|21349228	0.1362480	0.5134533	0.0000000	0.2616264	7
chr20:21319682|21324846	0.0000000	0.1158693	0.5418080	0.0000000	2
chr20:21346059|21349228	0.3254147	0.7701800	0.0000000	0.3924396	7
chr20:2473345|2474690	0.7084896	0.0000000	0.0000000	0.0000000	6
chr20:25201970|25203446	0.3269952	0.0000000	0.0000000	0.2616264	6
chr20:25271259|25273009	0.0000000	0.6518290	0.0000000	0.0000000	7
chr20:25394426|25398831	0.2350278	0.0000000	0.1031131	0.1869321	1

circRNA_coordinates	m6A-CTRL-S1	m6A-CTRL-S2	m6A-BTZ-S1	m6A-BTZ-S2	cluster_group
chr20:2928628|2945848	0.8174880	0.1711511	0.9289464	0.0000000	1
chr20:2944918|2945848	2.6432111	2.5672665	3.4835492	1.5697583	3
chr20:30380538|30382351	0.0000000	0.0855756	0.0000000	0.9156923	5
chr20:30904021|30904677	1.1444831	0.0855756	0.0000000	0.0000000	6
chr20:30954187|30956926	0.0000000	0.0855756	0.2322366	0.2616264	4
chr20:32684463|32686439	0.0000000	0.0434724	0.1955432	0.2616264	4
chr20:34300941|34313077	0.3269952	0.1711511	0.0000000	0.6540660	5
chr20:34302107|34313077	0.5449920	1.2836333	0.6967098	0.7848792	7
chr20:34302107|34320057	0.4087440	0.3423022	0.0000000	0.3924396	7
chr20:34304662|34313077	0.1634976	2.2944517	0.0000000	0.2616264	7
chr20:34309662|34313077	0.0000000	0.0000000	0.4644732	0.0000000	2
chr20:34309662|34320057	0.1089984	0.2567267	0.0000000	0.1308132	7
chr20:34317234|34320057	0.0000000	0.0608442	0.6967098	1.1511561	4
chr20:34317234|34326939	0.0000000	0.0000000	0.0000000	0.5232528	5
chr20:34388016|34459751	0.3814944	0.4278778	0.0000000	0.2616264	7
chr20:34389413|34459751	0.4087440	0.0000000	0.0000000	0.5232528	5
chr20:34446224|34459751	0.4087440	0.1711511	0.2322366	0.0000000	6
chr20:34450935|34459751	0.1089984	0.4278778	0.0000000	0.0000000	7
chr20:34450935|34487570	0.0000000	0.2567267	0.2322366	0.0000000	3
chr20:34501171|34505584	0.0000000	0.0000000	0.9289464	0.0000000	2
chr20:35421652|35467844	0.0000000	0.5990289	1.1611831	0.6540660	2
chr20:35693827|35696589	0.4087440	0.1711511	0.0000000	0.0000000	6
chr20:35812583|35812776	0.1634976	0.0652086	0.4644732	0.9156923	4
chr20:36685934|36694658	0.1362480	0.0825804	0.1955432	0.3924396	4
chr20:3802941|3804497	0.0000000	0.0000000	1.0838483	0.0000000	2

circRNA_coordinates	m6A-CTRL-S1	m6A-CTRL-S2	m6A-BTZ-S1	m6A-BTZ-S2	cluster_group
chr20:3888573|3893281	0.0000000	0.0000000	2.3223661	0.0000000	2
chr20:3888573|3899443	0.0000000	0.0000000	0.0000000	0.5232528	5
chr20:40141485|40143591	0.0000000	0.0000000	0.8051643	0.0000000	2
chr20:40141485|40162209	0.5994912	0.5990289	0.0000000	0.6540660	7
chr20:40141485|40179999	0.3814944	0.1711511	0.0000000	0.0000000	6
chr20:40161689|40162209	0.0000000	0.0000000	1.8578929	0.1308132	2
chr20:40161689|40179999	3.2972014	1.9682377	4.4124956	3.7935826	1
chr20:42331130|42333998	0.9537359	0.1711511	1.1611831	0.1308132	1
chr20:42338703|42340100	0.6267408	0.0000000	0.0000000	0.0000000	6
chr20:43607084|43615937	0.6297382	0.0000000	0.0000000	0.0000000	6
chr20:44980892|44983520	0.5722416	0.1477890	0.2322366	0.1308132	6
chr20:45874752|45875261	1.4714783	0.2567267	1.3934197	0.9156923	1
chr20:45874752|45878183	0.2997456	0.9413311	0.0000000	1.1773187	5
chr20:45891032|45905539	0.9537359	0.2567267	1.3934197	0.2616264	1
chr20:45905058|45910961	0.0000000	0.0000000	0.6967098	0.0000000	2
chr20:45905058|45916028	1.0354847	0.1711511	0.4644732	0.9156923	1
chr20:45905058|45976670	0.0000000	0.5134533	0.0000000	0.0000000	7
chr20:45910846|45976670	4.5498110	0.0000000	0.0000000	0.0000000	6
chr20:47567860|47570327	0.0000000	0.0000000	0.4644732	0.0000000	2
chr20:47570093|47580435	1.4169791	0.3423022	0.0000000	0.3924396	6
chr20:47570093|47592736	0.4087440	0.1711511	0.0000000	0.1495195	6
chr20:47881253|47888287	0.2452464	0.0000000	0.0000000	0.2616264	5
chr20:47886479|47888287	0.9809855	0.2567267	0.0000000	0.0000000	6
chr20:48257015|48273239	0.0000000	0.0000000	0.7022835	0.0000000	2
chr20:4864288|4880358	0.4971689	0.0000000	0.0000000	0.0456538	6

circRNA_coordinates	m6A-CTRL-S1	m6A-CTRL-S2	m6A-BTZ-S1	m6A-BTZ-S2	cluster_group
chr20:50048604|50052298	0.0000000	0.2567267	2.3223661	1.0465055	2
chr20:50048604|50140649	0.4632432	0.0000000	0.0000000	0.0000000	6
chr20:50071085|50140135	0.2724960	0.0855756	0.4644732	0.0000000	1
chr20:50133323|50140135	0.0000000	0.0000000	0.6967098	0.0000000	2
chr20:50133323|50140649	1.9074719	0.0000000	0.9289464	0.7848792	1
chr20:54956489|54961589	0.2997456	0.4278778	0.4644732	0.0000000	3
chr20:54956489|54963258	0.5449920	0.0000000	0.0000000	0.0000000	6
chr20:57014001|57016139	0.4632432	0.5134533	0.2322366	0.0000000	3
chr20:58318162|58330419	0.0000000	0.2567267	1.1611831	0.6540660	2
chr20:58318162|58349545	0.5449920	0.3423022	0.6967098	0.2616264	1
chr20:58348334|58349545	0.7357392	0.0000000	3.0964107	1.0465055	1
chr20:61537239|61545758	0.5722416	0.0855756	0.2322366	0.2616264	1
chr20:61833639|61835159	0.2452464	0.0000000	0.9289464	0.6540660	4
chr20:62407031|62422143	2.6432111	1.3692088	2.7868393	2.7470770	1
chr20:62559688|62562535	0.0000000	0.0000000	1.4449762	0.0000000	2
chr21:17135210|17138460	0.2452464	0.2567267	0.0000000	0.1308132	7
chr21:22652898|22746333	0.1089984	0.3423022	0.0000000	0.0000000	7
chr21:27113884|27121431	0.0000000	0.0000000	0.4644732	0.0000000	2
chr21:30677525|30702014	0.3269952	0.0000000	0.4644732	0.0000000	1
chr21:30693542|30699714	0.0000000	0.0000000	0.4644732	0.0000000	2
chr21:30693542|30702014	0.0000000	0.7701800	1.6256563	0.7848792	2
chr21:33694915|33696800	0.4087440	0.0000000	0.0000000	0.0000000	6
chr21:37619815|37620866	0.4087440	0.0898543	0.0000000	0.0523253	6
chr21:37620867|37623436	0.0000000	0.0000000	0.6967098	0.0000000	2
chr21:38480692|38501363	0.1506630	0.0000000	0.0000000	0.2616264	5

circRNA_coordinates	m6A-CTRL-S1	m6A-CTRL-S2	m6A-BTZ-S1	m6A-BTZ-S2	cluster_group
chr21:38792601|38794168	0.1362480	0.0855756	2.2835826	0.7848792	2
chr21:38792601|38845182	0.2997456	0.0000000	1.7547798	0.2287923	2
chr21:40578034|40584633	0.0000000	0.0855756	0.0000000	0.7848792	5
chr21:40578034|40601362	0.0000000	0.1711511	0.0000000	0.2616264	5
chr21:40596334|40610499	0.2179968	0.0000000	0.0000000	0.2616264	5
chr21:40600426|40601362	0.0000000	0.0000000	0.4644732	0.2616264	2
chr21:40641825|40646398	0.1332233	0.0000000	0.2322366	0.2616264	4
chr21:44424436|44448984	0.0903869	0.0855756	0.6967098	0.0000000	2
chr21:45786637|45789226	0.4359936	0.0000000	0.0000000	0.0000000	6
chr21:45826649|45833748	0.0000000	0.0000000	0.4644732	0.0000000	2
chr21:46275125|46281186	0.3268044	0.0000000	0.0977716	0.0000000	6
chr21:46354322|46354848	0.0000000	0.0000000	0.4644732	0.0000000	2
chr21:47703982|47705308	0.0000000	0.3423022	0.0000000	0.1308132	7
chr21:47783395|47787054	0.5994912	0.0855756	0.0000000	0.2616264	6
chr21:47808657|47811291	0.5177424	0.0000000	0.0000000	0.0000000	6
chr22:17584384|17585700	0.0000000	0.0000000	0.4105943	0.0000000	2
chr22:18165980|18185152	0.7902384	0.1233999	0.0000000	0.0000000	6
chr22:18304193|18304936	0.0000000	0.0000000	0.9805030	0.0000000	2
chr22:20126717|20131279	0.3814944	0.0855756	0.0000000	0.0000000	6
chr22:20800730|20825802	0.0000000	0.5134533	0.0000000	0.2616264	7
chr22:21288067|21288532	4.4689342	2.1393888	2.0901295	2.6162639	6
chr22:24032586|24037359	0.1907472	0.0000000	0.4644732	0.0000000	1
chr22:24143269|24145455	0.3542448	0.0855756	0.0000000	0.0000000	6
chr22:26936755|26940641	0.0000000	0.0855756	0.0000000	0.3924396	5
chr22:28559080|28559587	0.6267408	0.3423022	0.0000000	0.2616264	6

circRNA_coordinates	m6A-CTRL-S1	m6A-CTRL-S2	m6A-BTZ-S1	m6A-BTZ-S2	cluster_group
chr22:29115383|29121355	0.0000000	0.0000000	0.6967098	0.0000000	2
chr22:30738899|30740921	0.3542448	0.0855756	0.0000000	0.0000000	6
chr22:30980741|30983272	0.0000000	0.2567267	0.0000000	0.2616264	5
chr22:31998198|31998771	1.7897809	0.0000000	0.0000000	0.0000000	6
chr22:32154532|32164849	0.0000000	0.0782161	0.4644732	0.0000000	2
chr22:35653551|35658284	0.0000000	0.0000000	0.4644732	0.0000000	2
chr22:36714414|36715584	1.0606089	0.0000000	0.0000000	0.0000000	6
chr22:36737415|36745300	0.0000000	0.0000000	0.0000000	0.6950105	5
chr22:37330037|37331361	0.0000000	0.0000000	0.4644732	0.0000000	2
chr22:38019334|38019640	0.3269952	0.0000000	0.0000000	0.1308132	6
chr22:38641941|38644025	0.0000000	1.0429948	0.1955432	0.0000000	7
chr22:38890635|38897285	0.2724960	0.1711511	0.0000000	0.0000000	7
chr22:38895405|38897285	0.0000000	0.0000000	2.5018850	0.0896070	2
chr22:38917613|38964294	0.0544992	0.0299514	0.9289464	0.3924396	2
chr22:38933597|38964294	0.5994912	0.0000000	0.0000000	0.0000000	6
chr22:39709756|39710111	0.0000000	0.0000000	0.0000000	0.5232528	5
chr22:40642019|40697337	0.1362480	0.0855756	0.2322366	0.0000000	3
chr22:40825610|40831558	0.2358453	0.0855756	0.6321481	0.0000000	1
chr22:41265003|41282519	0.1362480	0.0000000	0.6967098	0.1308132	2
chr22:41521868|41537226	0.9039237	0.0000000	0.0000000	0.0000000	6
chr22:41664101|41677086	0.2724960	0.0000000	1.6514345	0.0000000	2
chr22:41928770|41936702	0.0000000	0.4278778	0.0000000	0.0000000	7
chr22:42204879|42206295	0.1634976	0.2567267	0.1031131	0.0000000	7
chr22:42557387|42565852	0.5994912	0.0000000	0.0000000	0.0000000	6
chr22:42605657|42611347	0.0000000	0.0855756	0.6967098	0.0000000	2

circRNA_coordinates	m6A-CTRL-S1	m6A-CTRL-S2	m6A-BTZ-S1	m6A-BTZ-S2	cluster_group
chr22:43627912|43634843	0.6539904	0.0000000	0.0000000	0.0000000	6
chr22:45574119|45574781	1.0899839	0.4278778	0.0000000	0.1308132	6
chr22:45818170|45822055	0.0817488	0.0000000	0.0000000	0.3924396	5
chr22:46125305|46136418	0.0000000	0.0000000	0.0000000	0.5232528	5
chr22:46134611|46136418	0.8496970	0.0000000	0.0000000	0.0000000	6
chr22:46493891|46494349	1.2419277	0.1991343	0.9289464	0.1308132	6
chr22:47022648|47033857	0.3542448	0.0631548	0.0000000	0.0000000	6
chr22:50187682|50198008	0.1362480	0.4278778	0.0000000	0.2616264	7
chr22:50197852|50217979	0.5449920	0.1711511	0.0000000	0.0000000	6
chr22:50688155|50688288	0.0000000	0.0000000	0.5866297	0.0000000	2
chr22:50810449|50832564	0.3269952	0.5134533	1.8578929	3.6627694	4
chr22:50810449|50845304	0.0544992	0.0000000	1.6256563	0.5232528	2
chr2:100065798|100081447	0.1332233	0.0855756	0.0000000	0.2616264	5
chr2:100623094|100625394	2.7249599	1.7115110	1.8578929	2.2238243	6
chr2:100623094|100628033	3.3244510	2.1393888	3.2513126	3.5319562	1
chr2:10281981|10282502	0.0000000	0.0000000	0.4644732	0.0692002	2
chr2:106471504|106471745	0.0000000	0.0000000	0.4644732	0.0000000	2
chr2:106761646|106782539	1.9892207	1.2254419	1.8578929	1.1773187	1
chr2:106774514|106782539	0.1634976	0.5134533	1.3934197	0.3924396	2
chr2:10729169|10747437	0.0000000	0.0855756	0.6967098	0.0000000	2
chr2:10927407|10930015	0.0000000	0.0000000	0.0000000	0.5232528	5
chr2:109388157|109389502	0.2452464	0.0855756	0.0000000	0.7848792	5
chr2:111406811|111419418	0.0000000	0.4278778	0.0000000	0.0000000	7
chr2:112821739|112824644	0.5785090	0.0000000	0.0000000	0.0000000	6
chr2:112994139|112996105	0.0000000	0.0000000	0.0000000	0.5232528	5

circRNA_coordinates	m6A-CTRL-S1	m6A-CTRL-S2	m6A-BTZ-S1	m6A-BTZ-S2	cluster_group
chr2:118693071|118698873	0.0000000	0.0000000	0.5866297	0.0000000	2
chr2:122260743|122287901	0.0120988	0.0000000	0.4644732	0.1308132	2
chr2:122363277|122363756	1.5532271	1.1980577	0.4644732	1.8313847	5
chr2:128252448|128281367	0.0000000	0.4278778	0.0000000	0.0000000	7
chr2:128260391|128281367	0.6267408	0.0855756	0.0000000	0.0000000	6
chr2:128627028|128628933	0.5875558	0.0000000	0.0000000	0.0000000	6
chr2:128627028|128631846	0.0000000	0.5134533	0.0000000	0.0000000	7
chr2:128744359|128758028	0.0000000	0.0000000	0.6967098	0.0000000	2
chr2:128914826|128922399	0.0000000	0.0000000	0.0000000	1.3455445	5
chr2:128922304|128928858	1.5668247	0.0000000	0.7998229	0.1308132	6
chr2:135010666|135012215	1.6894751	0.5990289	3.0190760	1.9621979	1
chr2:145274845|145274986	0.1089984	0.2567267	0.0000000	0.2616264	5
chr2:148730308|148739650	0.7084896	0.0000000	0.2322366	0.0000000	6
chr2:149539213|149542570	0.0000000	0.0000000	0.6967098	0.0000000	2
chr2:15415608|15567918	0.4359936	0.0000000	0.0000000	0.0000000	6
chr2:159519958|159522886	0.0000000	0.0000000	0.9289464	0.0000000	2
chr2:160229556|160245996	0.0000000	0.0855756	0.4644732	0.0000000	2
chr2:160294814|160473112	0.4087440	0.0000000	0.0000000	0.0000000	6
chr2:160585520|160619504	0.0000000	0.0000000	0.4644732	0.2616264	2
chr2:160602281|160605414	0.0000000	0.0000000	0.0000000	0.9156923	5
chr2:160602281|160609082	0.0000000	0.0000000	0.2322366	0.3394602	4
chr2:160604625|160605308	0.0000000	0.0000000	0.6967098	0.2616264	2
chr2:170387097|170401345	0.0000000	0.0000000	0.2322366	0.3924396	4
chr2:170393737|170413807	0.7629888	0.2567267	0.0000000	0.2616264	6
chr2:170396561|170413807	0.0000000	0.0000000	0.0000000	1.0465055	5

circRNA_coordinates	m6A-CTRL-S1	m6A-CTRL-S2	m6A-BTZ-S1	m6A-BTZ-S2	cluster_group
chr2:170401231|170413807	5.7496653	3.5085976	4.1802590	2.7470770	6
chr2:170411634|170413807	0.0000000	0.1497572	0.2933148	0.0000000	3
chr2:171902684|171906684	0.0000000	0.0000000	0.6967098	0.0000000	2
chr2:172193963|172196064	0.9809855	0.4278778	0.2322366	0.7848792	6
chr2:174819601|174820960	2.5342127	1.6259355	3.9480224	4.3168354	4
chr2:179400459|179407088	0.8447376	0.4278778	0.2322366	1.1773187	5
chr2:180842917|180853371	0.0000000	0.0000000	0.4644732	0.0000000	2
chr2:182786675|182793005	0.2410499	0.0000000	0.4644732	0.2616264	1
chr2:191520703|191537878	0.9537359	0.0000000	0.0000000	0.6540660	6
chr2:191523884|191537878	0.3269952	0.1711511	1.1611831	0.6540660	2
chr2:196544756|196548630	1.8529727	0.7701800	0.0000000	0.6540660	6
chr2:200173483|200233430	0.6363599	0.0000000	0.0000000	0.0000000	6
chr2:200173483|200298237	1.2474321	0.0000000	0.0000000	0.0000000	6
chr2:200188526|200233430	0.0000000	0.4278778	0.4644732	0.0000000	3
chr2:200188526|200246543	0.0000000	0.0000000	0.4644732	0.0000000	2
chr2:200213424|200298237	0.0000000	0.0000000	1.1611831	0.0000000	2
chr2:200233328|200298237	0.0000000	0.2695630	5.1607620	0.1308132	2
chr2:200245087|200298237	0.2724960	0.3423022	0.0000000	1.0216510	5
chr2:201994452|202014558	0.2997456	0.0000000	1.3934197	0.2616264	2
chr2:202005080|202028807	3.8507770	0.0000000	0.0000000	0.0000000	6
chr2:202010101|202014558	0.0407926	0.0000000	5.7285805	0.3924396	2
chr2:202625604|202626541	0.9809855	0.2567267	0.9289464	0.7848792	1
chr2:20478344|20527139	0.3269952	0.5134533	0.0000000	0.0000000	7
chr2:20507739|20527139	0.4359936	0.3423022	0.1720873	0.0000000	6
chr2:208461638|208462207	0.0000000	0.1711511	0.0000000	0.2616264	5

circRNA_coordinates	m6A-CTRL-S1	m6A-CTRL-S2	m6A-BTZ-S1	m6A-BTZ-S2	cluster_group
chr2:208841375|208842310	3.7331950	3.0807199	8.8249912	7.0639124	4
chr2:210962810|211019335	0.2452464	0.1711511	0.0000000	0.0000000	7
chr2:210968828|211019335	0.0272496	0.2567267	0.9289464	0.7848792	4
chr2:210993755|211019335	0.0000000	0.0000000	0.4644732	0.1308132	2
chr2:211018219|211019335	0.5994912	0.2567267	0.2322366	0.0000000	6
chr2:215609791|215646233	0.0544992	0.4278778	0.0000000	0.0000000	7
chr2:215609791|215661841	0.0000000	0.0000000	0.4644732	0.0000000	2
chr2:215617171|215646233	0.7357392	0.3423022	0.2322366	0.0000000	6
chr2:215632206|215646233	6.1856589	2.0538132	3.2513126	3.4011430	1
chr2:215632206|215661841	1.3079807	0.9413311	0.0000000	0.5232528	7
chr2:215645284|215661841	0.7357392	0.0000000	0.0000000	0.1308132	6
chr2:217279370|217285255	0.3542448	0.0000000	0.0000000	0.1308132	6
chr2:219520873|219521186	0.7084896	0.0000000	0.0000000	0.0000000	6
chr2:224640618|224642586	0.0000000	0.0000000	1.1611831	0.0000000	2
chr2:227729320|227732034	0.3814944	0.0855756	1.3934197	0.2616264	2
chr2:227729320|227779067	0.6539904	0.1711511	1.1611831	0.6540660	1
chr2:230705538|230725247	0.0000000	0.0000000	0.0000000	0.6540660	5
chr2:230723488|230725247	0.0000000	0.0855756	0.0977716	1.0465055	5
chr2:230723488|230744844	0.7357392	0.0000000	0.0000000	0.0000000	6
chr2:231305191|231314970	0.0000000	0.0000000	0.0000000	1.0465055	5
chr2:231738273|231740303	0.0000000	0.0000000	0.0000000	0.5232528	5
chr2:231940225|231951895	0.2179968	0.0130075	0.4644732	0.3924396	1
chr2:233651860|233660931	0.0000000	0.2567267	0.2322366	1.7005715	5
chr2:234171776|234178713	0.3814944	0.0000000	0.0000000	0.1308132	6
chr2:234343026|234347025	0.3269952	0.0855756	0.3095714	0.0000000	1

circRNA_coordinates	m6A-CTRL-S1	m6A-CTRL-S2	m6A-BTZ-S1	m6A-BTZ-S2	cluster_group
chr2:234343026|234350641	0.0000000	0.0000000	0.6967098	0.0000000	2
chr2:239090706|239093928	0.2179968	0.0000000	0.2322366	0.0000000	1
chr2:239090706|239103511	0.0272496	0.0000000	0.9289464	0.2616264	2
chr2:240929491|240946787	0.0000000	0.0855756	0.0257783	0.6540660	5
chr2:24098699|24118840	0.0000000	0.0000000	0.4644732	0.0000000	2
chr2:241439375|241494472	0.0000000	0.0000000	0.4644732	0.0000000	2
chr2:241463302|241494472	0.0000000	0.2567267	0.2322366	0.6540660	5
chr2:241465883|241468453	0.0000000	0.6846044	0.0000000	0.0000000	7
chr2:242194466|242196221	0.4632432	0.0000000	0.0000000	0.0000000	6
chr2:242572332|242573494	1.0354847	0.3423022	2.0901295	0.7848792	1
chr2:242608101|242610122	0.0000000	0.3423022	0.0000000	0.5232528	5
chr2:242644068|242651486	0.5994912	0.0000000	2.0901295	0.0000000	1
chr2:242651393|242662686	0.4904928	0.2567267	0.4644732	0.2616264	1
chr2:25044539|25045379	2.0160615	0.0000000	0.0000000	0.0000000	6
chr2:25990452|25994409	0.3542448	0.0000000	0.6967098	0.3924396	1
chr2:26321531|26332775	0.2179968	0.0173718	0.0000000	0.2616264	5
chr2:27324965|27333836	0.4087440	0.1711511	0.4644732	0.0000000	1
chr2:27424985|27425680	0.0000000	0.0000000	0.9289464	0.0000000	2
chr2:27456009|27456150	0.0000000	0.1797087	0.1955432	0.1046506	2
chr2:27591031|27591238	0.0000000	0.0000000	0.6967098	0.0000000	2
chr2:27597626|27597905	0.0000000	0.0000000	0.4644732	0.0000000	2
chr2:29124853|29159819	0.0000000	0.0000000	0.4644732	0.0000000	2
chr2:3197770|3200788	0.4866233	0.0000000	0.0000000	0.0000000	6
chr2:32142995|32157204	0.1089984	0.0898543	0.5866297	0.0000000	2
chr2:32142995|32168452	0.2452464	0.0000000	0.0000000	0.3924396	5

circRNA_coordinates	m6A-CTRL-S1	m6A-CTRL-S2	m6A-BTZ-S1	m6A-BTZ-S2	cluster_group
chr2:32602656|32631625	0.0000000	0.0000000	0.4644732	0.1308132	2
chr2:32626231|32658872	0.4359936	0.1711511	0.2322366	0.0000000	6
chr2:32631567|32641231	0.4087440	0.0000000	0.0000000	0.0000000	6
chr2:37227729|37235991	0.1089984	0.0000000	0.9289464	0.0000000	2
chr2:37543380|37544322	1.7712239	1.1980577	1.3934197	0.5232528	6
chr2:40655613|40657444	1.0082351	0.3423022	0.4644732	0.7848792	1
chr2:44132831|44175657	0.0000000	0.0212227	0.6967098	0.0000000	2
chr2:44428325|44436466	0.9809855	1.3692088	3.2513126	2.0930111	2
chr2:44428325|44445676	0.0000000	0.5990289	0.6967098	1.7005715	5
chr2:45773871|45789895	0.1907472	0.3423022	0.2322366	0.6540660	5
chr2:48049357|48050499	0.0000000	0.1711511	0.0000000	0.3737333	5
chr2:48555700|48573890	1.5259775	1.1124822	0.0000000	0.0000000	7
chr2:48555700|48586286	0.4087440	0.0000000	0.0000000	0.5232528	5
chr2:48573340|48573890	0.8447376	0.4278778	0.0000000	1.1773187	5
chr2:48701822|48718309	0.0000000	0.0855756	0.6967098	0.0000000	2
chr2:48807726|48809702	1.1717327	0.1711511	0.0000000	0.0000000	6
chr2:53921021|53978078	0.1634976	0.0000000	0.0000000	0.6540660	5
chr2:55867762|55870560	0.0000000	0.0000000	0.4644732	0.0000000	2
chr2:58311224|58316858	0.3269952	0.0000000	0.0000000	0.2616264	6
chr2:58449077|58459247	0.1634976	0.0261005	0.2933148	0.1308132	1
chr2:61505300|61508377	0.0000000	0.0000000	0.0000000	0.5232528	5
chr2:61719170|61724142	0.0000000	0.1711511	0.4644732	0.0000000	2
chr2:61749746|61761038	0.5994912	0.0855756	0.4644732	0.5232528	1
chr2:62103230|62110683	0.0000000	0.0000000	0.4644732	0.0000000	2
chr2:63206323|63223901	0.0000000	0.2567267	0.0000000	0.3924396	5

circRNA_coordinates	m6A-CTRL-S1	m6A-CTRL-S2	m6A-BTZ-S1	m6A-BTZ-S2	cluster_group
chr2:63631183|63631792	0.0000000	0.0000000	0.4644732	0.0000000	2
chr2:63711738|63798760	0.0000000	0.0000000	0.6967098	0.0000000	2
chr2:63798509|63798760	0.0000000	0.0000000	0.4644732	0.1308132	2
chr2:64083440|64085070	0.3542448	0.1043166	0.0000000	0.1308132	6
chr2:64189192|64211153	0.3133704	0.2567267	0.0000000	0.0000000	7
chr2:64778577|64780543	0.0000000	0.6846044	0.0000000	0.0000000	7
chr2:65318117|65325200	0.0000000	0.0000000	0.4644732	0.0000000	2
chr2:68413600|68415822	3.6082011	0.0000000	0.0000000	0.0000000	6
chr2:70387757|70409129	0.0000000	0.0000000	0.0000000	0.7848792	5
chr2:70396689|70409129	0.2724960	0.0000000	0.6967098	0.2616264	1
chr2:70402805|70409129	0.6539904	0.0000000	0.0000000	0.2616264	6
chr2:70406664|70409129	1.4169791	0.0000000	1.8578929	0.5232528	1
chr2:70451686|70457986	0.7629888	0.2567267	0.0000000	0.0000000	6
chr2:71582849|71597135	0.0000000	0.5134533	0.0000000	0.0000000	7
chr2:71590283|71595658	0.0000000	0.0000000	0.4644732	0.0000000	2
chr2:71590283|71597135	0.0000000	0.5134533	0.0000000	0.0000000	7
chr2:71623271|71645769	0.0000000	0.4278778	0.0000000	0.1308132	7
chr2:72945232|72960247	0.0000000	0.0000000	0.2933148	0.2616264	4
chr2:74213107|74230293	0.0000000	0.0000000	0.0000000	0.5232528	5
chr2:74273405|74275538	0.0000000	0.0855756	0.6967098	0.2616264	2
chr2:85595809|85596953	0.4632432	0.0000000	0.0000000	0.0000000	6
chr2:85595809|85598685	0.5177424	0.0855756	0.2322366	0.0000000	6
chr2:86335473|86346167	0.4632432	0.0000000	0.0000000	0.0000000	6
chr2:86378420|86393767	0.0000000	0.0000000	0.6967098	0.0000000	2
chr2:96950344|96950917	0.0000000	0.0599029	0.6967098	0.0000000	2

circRNA_coordinates	m6A-CTRL-S1	m6A-CTRL-S2	m6A-BTZ-S1	m6A-BTZ-S2	cluster_group
chr2:99786013|99787892	0.7629888	0.0898543	0.0000000	0.1308132	6
chr3:10136048|10136856	0.0000000	0.0000000	0.4644732	0.0000000	2
chr3:101374948|101391057	0.9809855	0.1711511	0.0000000	0.0000000	6
chr3:101389974|101391057	1.8529727	0.5134533	1.8578929	1.7005715	1
chr3:10301821|10302354	0.0000000	0.4278778	0.0000000	0.0000000	7
chr3:114069121|114070725	0.3269952	0.0000000	0.0000000	0.6540660	5
chr3:11849307|11858811	0.0000000	0.0000000	0.4644732	0.0000000	2
chr3:119582266|119624699	0.2724960	0.0000000	0.7310809	0.0000000	1
chr3:120469370|120469847	0.5449920	0.0855756	0.4644732	0.0746943	1
chr3:121354582|121356103	0.0000000	0.0000000	2.1674643	0.0000000	2
chr3:121563300|121591635	0.0544992	0.0000000	0.4644732	0.0000000	2
chr3:123649949|123667979	0.0000000	0.5990289	0.2322366	0.5232528	5
chr3:124996570|125032500	0.0000000	0.4278778	0.0000000	0.6540660	5
chr3:124996570|125050082	1.6622255	0.0000000	0.0000000	0.0000000	6
chr3:125006947|125033897	0.0000000	0.0000000	0.0000000	0.6540660	5
chr3:125006947|125050082	0.9537359	0.4278778	0.4644732	0.5232528	6
chr3:125032152|125032500	1.7439743	0.2567267	0.2322366	0.0000000	6
chr3:125032152|125042288	0.0000000	0.3423022	0.6967098	0.6540660	5
chr3:125032152|125050077	1.1172335	0.3423022	0.0000000	0.3924396	6
chr3:125032152|125050082	48.5315350	15.4035993	35.0677283	34.5346828	1
chr3:12538017|12545283	0.5449920	0.0000000	0.0000000	0.0000000	6
chr3:127358088|127381154	0.3814944	0.0000000	0.2322366	0.0000000	6
chr3:127379221|127381154	2.6977103	1.2836333	0.2322366	0.1308132	6
chr3:127783881|127785767	1.5457335	0.0000000	0.0000000	0.0000000	6
chr3:128350791|128363826	0.0000000	0.6649220	0.0000000	0.0000000	7

circRNA_coordinates	m6A-CTRL-S1	m6A-CTRL-S2	m6A-BTZ-S1	m6A-BTZ-S2	cluster_group
chr3:129174957|129183624	0.0000000	0.0000000	0.4644732	0.0000000	2
chr3:129182403|129188260	0.0000000	0.1477890	0.2933148	0.0000000	3
chr3:129546646|129551669	0.4087440	0.0855756	1.3934197	0.7848792	2
chr3:12976948|12983365	0.5994912	0.1711511	0.0000000	0.0000000	6
chr3:13381832|13383247	0.0000000	0.1711511	0.2322366	0.0000000	3
chr3:133894453|133914026	0.2997456	0.0000000	0.0000000	0.1308132	6
chr3:135979321|135980907	0.1634976	0.2567267	0.0000000	0.2616264	7
chr3:136664425|136665137	0.0000000	0.0000000	0.4644732	0.0000000	2
chr3:138244332|138291826	0.2997456	0.0000000	0.0000000	0.1308132	6
chr3:138289160|138291774	0.7357392	0.0000000	0.6967098	1.7005715	4
chr3:138289160|138291826	0.0000000	0.0000000	0.4644732	0.1308132	2
chr3:14513826|14518686	0.0000000	0.0000000	0.4644732	0.0000000	2
chr3:14763160|14770195	0.0000000	0.0000000	0.0000000	0.6540660	5
chr3:149570303|149613347	0.0000000	0.0000000	0.6967098	0.0000000	2
chr3:152017194|152018156	1.6894751	1.0269066	2.3223661	2.6162639	4
chr3:152017194|152018762	0.3269952	0.0855756	0.0000000	0.3924396	5
chr3:152132730|152165562	0.0817488	0.3893688	0.0000000	0.0218458	7
chr3:152132730|152174150	0.0000000	0.0000000	0.4644732	0.0000000	2
chr3:155545971|155560408	0.0000000	0.1711511	0.0000000	0.3924396	5
chr3:155551257|155560408	0.0000000	0.0000000	0.0000000	0.5232528	5
chr3:155611307|155629068	0.0000000	0.0000000	0.0000000	0.4858402	5
chr3:155628481|155643155	0.3542448	0.0000000	0.2322366	0.5232528	4
chr3:156395446|156413814	0.2452464	0.0855756	0.0000000	0.1308132	6
chr3:157839892|157841780	2.0437199	0.2567267	0.4644732	1.4389451	1
chr3:157839892|157921034	0.0000000	0.0855756	0.6967098	0.2616264	2

circRNA_coordinates	m6A-CTRL-S1	m6A-CTRL-S2	m6A-BTZ-S1	m6A-BTZ-S2	cluster_group
chr3:167240103|167254788	0.0000000	0.0855756	0.2322366	0.1046506	2
chr3:167240103|167345080	0.0000000	0.1711511	0.2322366	0.0000000	3
chr3:167272438|167338391	0.0000000	0.4953969	0.0000000	0.0000000	7
chr3:167272438|167345080	0.2997456	0.0000000	0.0000000	0.3924396	5
chr3:167293706|167345080	0.2179968	0.0000000	0.0000000	0.2616264	5
chr3:167319915|167345080	0.5449920	0.2567267	2.3223661	0.5232528	2
chr3:167754624|167759262	0.8719872	0.4278778	0.2322366	0.0000000	6
chr3:167754624|167766153	0.4087440	0.0000000	0.0000000	0.0000000	6
chr3:169835014|169847340	0.0000000	0.2567267	0.4644732	0.0000000	3
chr3:169840379|169847340	0.6267408	0.8557555	0.9289464	0.9156923	5
chr3:169854207|169867032	0.8174880	0.0855756	0.5780369	0.5232528	1
chr3:169854207|169896726	0.5722416	0.0853188	0.0000000	0.0000000	6
chr3:169863211|169867032	0.0000000	0.1711511	0.2408294	1.7005715	5
chr3:170077487|170079217	0.0000000	0.0000000	0.2322366	0.3924396	4
chr3:17051166|17056403	1.9892207	0.9413311	0.4644732	0.6540660	6
chr3:171944661|171969331	0.3269952	0.0000000	0.0000000	0.1308132	6
chr3:171965323|171969331	1.5804767	0.3423022	1.1611831	1.5697583	1
chr3:179287612|179294701	0.0000000	0.0000000	1.1611831	0.0000000	2
chr3:179310432|179319578	0.0000000	0.0000000	1.8578929	0.0000000	2
chr3:180685839|180688146	0.4359936	0.1711511	0.2322366	0.0000000	6
chr3:183361268|183369064	1.2262319	0.2567267	0.0000000	0.7848792	6
chr3:183361268|183371877	0.0000000	0.0000000	0.9289464	0.0000000	2
chr3:183361268|183382827	0.1634976	0.0000000	0.4644732	0.5232528	4
chr3:183368084|183369064	0.8719872	0.5990289	0.0000000	1.3081319	5
chr3:183454506|183504089	0.0000000	0.0000000	1.1611831	0.0000000	2

circRNA_coordinates	m6A-CTRL-S1	m6A-CTRL-S2	m6A-BTZ-S1	m6A-BTZ-S2	cluster_group
chr3:183465460|183480067	0.0000000	0.0000000	0.4644732	0.1308132	2
chr3:183519317|183521017	0.4632432	0.0000000	0.0000000	0.0000000	6
chr3:18393484|18462483	0.2997456	0.2567267	0.2322366	0.0996797	3
chr3:18419662|18438782	0.0000000	0.3423022	0.4644732	0.0000000	3
chr3:18419662|18458570	2.0164703	1.0269066	0.0000000	2.4854507	5
chr3:18419662|18462483	32.3997726	28.9245364	61.0782288	43.0375404	2
chr3:18427891|18462483	0.0000000	0.1711511	0.9289464	0.2616264	2
chr3:18435954|18462483	1.5259775	0.0000000	0.6967098	0.3924396	1
chr3:18456603|18462483	2.2072175	1.5403599	1.8578929	1.8313847	1
chr3:185638892|185639914	0.3542448	0.0000000	0.4751561	0.6540660	4
chr3:185638892|185641772	0.2724960	0.0529713	0.0000000	0.2616264	5
chr3:186501429|186502197	0.0000000	0.0463819	1.1611831	0.0062790	2
chr3:194371844|194387301	0.0000000	0.0000000	0.6967098	0.0000000	2
chr3:195012435|195018010	0.0000000	0.0000000	0.6967098	0.0000000	2
chr3:195415404|195416309	0.9809855	1.2836333	0.0000000	1.8313847	5
chr3:195605124|195615477	0.1089984	0.0000000	1.1869613	0.2616264	2
chr3:195798267|195803993	0.0000000	0.0000000	0.6967098	0.0000000	2
chr3:196118684|196120490	0.5994912	0.0000000	0.0000000	0.6540660	5
chr3:196118684|196129890	2.5614623	0.8557555	2.5546027	2.0930111	1
chr3:196214270|196215554	0.0817488	0.0855756	0.6967098	0.0000000	2
chr3:196532223|196539722	0.3269952	0.5990289	0.0000000	0.0000000	7
chr3:196533450|196534785	0.8356634	0.0000000	1.3934197	0.0000000	1
chr3:196533450|196539722	0.4087440	0.0521155	0.0000000	0.0000000	6
chr3:196612022|196613565	0.2997456	0.3423022	0.0000000	0.3924396	7
chr3:197009550|197009716	0.2847311	0.0000000	0.0000000	0.2616264	5

circRNA_coordinates	m6A-CTRL-S1	m6A-CTRL-S2	m6A-BTZ-S1	m6A-BTZ-S2	cluster_group
chr3:197557641|197566268	0.0000000	0.0000000	2.7868393	0.0000000	2
chr3:197592294|197602646	0.2853850	0.0855756	0.4888581	0.2616264	1
chr3:27490142|27493989	0.0000000	0.0000000	0.5866297	0.0523253	2
chr3:31617888|31621588	0.7902384	0.0000000	0.6967098	0.0000000	1
chr3:3178944|3182332	1.3079807	0.0599029	0.4644732	0.7848792	1
chr3:3178944|3189871	0.0000000	0.0000000	0.0000000	0.5232528	5
chr3:3209318|3216953	0.0000000	0.0855756	0.0000000	0.5232528	5
chr3:32582529|32587457	0.4904928	0.4278778	0.4644732	1.3081319	5
chr3:33725851|33738425	0.2530943	0.0303793	0.4644732	0.1308132	1
chr3:41265512|41280845	0.0000000	0.0855756	0.0000000	0.3924396	5
chr3:43341246|43345284	0.8719872	0.0000000	0.1549018	0.2616264	1
chr3:44826337|44835918	0.2997456	0.1198058	0.0000000	0.0000000	6
chr3:47079156|47088111	0.9992700	0.0000000	0.0000000	0.0000000	6
chr3:47079156|47098980	0.0000000	0.0000000	0.6967098	0.0000000	2
chr3:47079156|47103836	0.0000000	0.3423022	0.9289464	0.0000000	2
chr3:47079156|47108608	1.2262319	1.8826621	0.6967098	1.4389451	7
chr3:47087977|47108608	0.0817488	0.0855756	2.3223661	0.2616264	2
chr3:47098311|47098980	0.0000000	0.0000000	1.3934197	0.0000000	2
chr3:47098311|47103836	1.5259775	0.3423022	1.1611831	0.5232528	1
chr3:47098311|47108608	2.7249599	1.7970866	3.7157858	2.0930111	1
chr3:47108560|47139571	0.6812400	0.2567267	0.0000000	1.0465055	5
chr3:47464737|47465422	0.0000000	0.0000000	1.0838483	0.0000000	2
chr3:47727539|47734790	0.1332233	0.0855756	0.2322366	0.0716856	1
chr3:47956307|47958664	0.4632432	0.0000000	0.0000000	0.0000000	6
chr3:4821330|4824268	0.0000000	0.0000000	0.4644732	0.0000000	2

circRNA_coordinates	m6A-CTRL-S1	m6A-CTRL-S2	m6A-BTZ-S1	m6A-BTZ-S2	cluster_group
chr3:48789018|48793877	0.0605486	0.3476079	0.0000000	0.0000000	7
chr3:48960181|48965246	1.3079807	0.0000000	0.9289464	0.2616264	1
chr3:48964895|48965246	0.1362480	0.0000000	0.4644732	0.3924396	4
chr3:48964895|48982614	0.1907472	0.0000000	0.2322366	0.0000000	1
chr3:48964895|48986529	0.0000000	0.0000000	1.3934197	0.0000000	2
chr3:48964895|49002415	0.1089984	0.3423022	0.0000000	0.2616264	5
chr3:49081823|49114471	0.0000000	0.1711511	0.2322366	0.0000000	3
chr3:49094295|49095323	0.2997456	0.0000000	0.4644732	0.6540660	4
chr3:49147617|49156714	0.0000000	0.0000000	0.4644732	0.0000000	2
chr3:49323531|49323758	0.0000000	0.0000000	0.0000000	0.7848792	5
chr3:50004903|50012825	0.1089984	0.8557555	0.6967098	0.9156923	5
chr3:50102464|50103935	0.3269952	0.0855756	0.2322366	0.0000000	6
chr3:50131153|50142575	0.0000000	0.0855756	0.4386950	0.0000000	2
chr3:51474986|51477919	1.4442287	0.0000000	0.0000000	0.0000000	6
chr3:52238709|52240767	0.8447376	0.4278778	0.0000000	0.9156923	5
chr3:52441503|52441973	0.0000000	0.0000000	0.4644732	0.0000000	2
chr3:52446827|52448603	0.0000000	0.3128642	1.9610059	0.5606653	2
chr3:52964345|52966324	0.1362480	0.0000000	0.0000000	0.3924396	5
chr3:56626998|56628056	0.2724960	0.4278778	0.0977716	0.3924396	5
chr3:63898264|63898901	0.7902384	0.1520678	0.5866297	0.2616264	1
chr3:67546222|67579610	0.0000000	0.0000000	0.4644732	0.0000000	2
chr3:71064700|71102924	0.0000000	0.1435958	0.0000000	0.2616264	5
chr3:71090479|71102924	0.1089984	0.0000000	1.6256563	0.1308132	2
chr3:81691932|81720104	0.0000000	0.0000000	0.2933148	0.1308132	2
chr3:93722503|93722752	0.2724960	0.0855756	0.1549018	0.3924396	4

circRNA_coordinates	m6A-CTRL-S1	m6A-CTRL-S2	m6A-BTZ-S1	m6A-BTZ-S2	cluster_group
chr3:9486732|9487424	0.0000000	0.0000000	0.4644732	0.1308132	2
chr4:10090392|10099334	0.0000000	0.0000000	0.4644732	0.0000000	2
chr4:10099335|10105610	0.0000000	0.0000000	0.4644732	0.0000000	2
chr4:113483527|113506881	0.0000000	0.2567267	0.3613602	0.0000000	3
chr4:123848789|123900541	0.2997456	0.0029096	1.8578929	0.0000000	2
chr4:125631155|125632429	0.0000000	0.0000000	0.4644732	0.0000000	2
chr4:128995615|128999117	0.0000000	0.0000000	0.4644732	0.0000000	2
chr4:128995615|129003460	0.2452464	0.1711511	0.6967098	0.0000000	2
chr4:128995615|129012299	0.0000000	0.0000000	0.9289464	0.2616264	2
chr4:128995615|129012667	0.5994912	0.0299514	0.0000000	0.0000000	6
chr4:129913322|129925031	1.8802223	0.0000000	1.6256563	0.0000000	1
chr4:129960196|130003528	0.4087440	0.0000000	0.0000000	0.0000000	6
chr4:13583861|13589395	0.0000000	0.0000000	0.0000000	0.6540660	5
chr4:139981478|139994721	0.0000000	0.2567267	2.0901295	0.0000000	2
chr4:140046318|140060651	0.4359936	0.0000000	0.2322366	0.1308132	1
chr4:140314745|140325644	0.2724960	0.2567267	0.0000000	0.0000000	7
chr4:144464662|144465125	0.0000000	0.0000000	0.2322366	1.5697583	4
chr4:148575003|148575717	0.1907472	0.1711511	0.4644732	0.0000000	2
chr4:148800383|148803083	0.0783426	0.0000000	0.0000000	0.3924396	5
chr4:148867768|148887990	0.1907472	0.0898543	0.0000000	0.1308132	7
chr4:151719233|151753128	0.0000000	0.0000000	0.4644732	0.0000000	2
chr4:151727423|151753128	0.5722416	0.0000000	0.0000000	0.0000000	6
chr4:153332455|153333681	18.3662294	7.0171952	10.2184109	7.7179784	6
chr4:154315414|154318485	0.1907472	0.0599029	1.1611831	0.3924396	2
chr4:15626875|15646280	0.0000000	0.4278778	0.0000000	0.1308132	7

circRNA_coordinates	m6A-CTRL-S1	m6A-CTRL-S2	m6A-BTZ-S1	m6A-BTZ-S2	cluster_group
chr4:15626875|15646331	0.0000000	0.0000000	0.0000000	0.5232528	5
chr4:159789254|159791621	0.4359936	0.0000000	0.0000000	0.0000000	6
chr4:1696501|1705427	0.0000000	0.0000000	0.0000000	0.5232528	5
chr4:170498079|170511960	0.0000000	0.0000000	0.6967098	0.0000000	2
chr4:1729435|1730514	0.5994912	0.2567267	0.0000000	0.0000000	6
chr4:1737562|1738943	0.0000000	0.0000000	0.4644732	0.0124273	2
chr4:174169131|174169591	2.6977103	0.7701800	1.6256563	0.7848792	6
chr4:178260937|178274882	0.2997456	0.4278778	0.0000000	0.0000000	7
chr4:178260937|178281831	0.0000000	0.0000000	0.4644732	0.0000000	2
chr4:178272534|178274882	1.1444831	1.5403599	0.9289464	1.1773187	7
chr4:178272534|178281831	0.0000000	0.4278778	0.4644732	0.0000000	3
chr4:178274462|178274882	0.8447376	1.4547844	1.1611831	1.8313847	5
chr4:178274462|178281831	6.4581548	6.4181664	8.1282814	3.1395166	3
chr4:185573177|185580591	0.0000000	0.0000000	0.4644732	0.0000000	2
chr4:1902353|1906105	2.7794590	0.5134533	0.0000000	1.5697583	6
chr4:1902353|1920350	1.1444831	0.9413311	0.6967098	1.0465055	7
chr4:1902353|1932497	3.0792046	1.8826621	2.0901295	1.0465055	6
chr4:1902353|1936989	1.6622255	1.1980577	0.0000000	1.9621979	5
chr4:1902353|1941505	0.4359936	0.8557555	0.0000000	0.9156923	5
chr4:1902353|1952930	1.1444831	0.4278778	1.1611831	0.0000000	1
chr4:1905943|1920350	0.9685052	0.0000000	0.0000000	0.3924396	6
chr4:1905943|1936989	0.0000000	0.0000000	0.2322366	0.3924396	4
chr4:1918598|1920350	0.0000000	0.0000000	0.0000000	0.9156923	5
chr4:25766948|25792196	0.0000000	0.0000000	0.9289464	0.0000000	2
chr4:2701407|2702271	0.2724960	0.0000000	0.0000000	0.9156923	5

circRNA_coordinates	m6A-CTRL-S1	m6A-CTRL-S2	m6A-BTZ-S1	m6A-BTZ-S2	cluster_group
chr4:2951661|2952972	1.9347215	0.6846044	1.1611831	1.0465055	1
chr4:2951661|2955372	0.4632432	0.1711511	1.1611831	0.9156923	4
chr4:2954002|2958538	0.5177424	0.0000000	0.0000000	0.0000000	6
chr4:38037209|38055959	0.0000000	0.5134533	0.6967098	0.3924396	2
chr4:38045983|38104778	0.1634976	0.0000000	0.9289464	0.0000000	2
chr4:39313065|39329376	0.2724960	0.0000000	0.0000000	0.2616264	5
chr4:39839476|39843676	0.0000000	0.0000000	0.4644732	0.0000000	2
chr4:39839476|39847500	0.5994912	0.0000000	0.0000000	0.0000000	6
chr4:40075075|40115128	0.0000000	0.2567267	0.0000000	0.3924396	5
chr4:40439788|40440941	0.0000000	0.0000000	0.2322366	0.6540660	4
chr4:4281174|4281476	0.0000000	0.0521155	0.0000000	0.3924396	5
chr4:48172265|48173466	0.0000000	0.0000000	0.6967098	0.0000000	2
chr4:48853823|48859382	0.2724960	0.0000000	0.4644732	0.1308132	1
chr4:52729603|52758017	0.0000000	0.0000000	0.0000000	0.9156923	5
chr4:56277781|56284152	0.0847735	0.0855756	0.2064583	0.1308132	2
chr4:56749989|56763066	0.0000000	0.0000000	0.0000000	0.5232528	5
chr4:56877578|56878151	0.1634976	0.0000000	0.0000000	0.3924396	5
chr4:6925100|6925838	0.6539904	0.0000000	0.0000000	0.0000000	6
chr4:6925100|6969151	0.2724960	0.1711511	0.0000000	0.0000000	7
chr4:73944359|73958017	0.0000000	0.0855756	0.2322366	0.1308132	2
chr4:73950966|73958017	1.3624799	0.3423022	1.6256563	0.7848792	1
chr4:73950966|73991029	0.0000000	0.0000000	0.6967098	0.0000000	2
chr4:73956384|73958017	1.8802223	1.1124822	4.8769688	3.0087034	2
chr4:73984405|73991029	0.0000000	0.0000000	0.4644732	0.0000000	2
chr4:76573823|76581054	0.0000000	0.0855756	0.0257783	0.3924396	5

circRNA_coordinates	m6A-CTRL-S1	m6A-CTRL-S2	m6A-BTZ-S1	m6A-BTZ-S2	cluster_group
chr4:77065302|77065626	0.0000000	0.0000000	0.2322366	0.3924396	4
chr4:78694235|78697546	0.5994912	0.4278778	0.2580149	0.0000000	6
chr4:83900036|83906029	0.0000000	0.0855756	0.6967098	0.1308132	2
chr4:87967318|87968746	21.2819364	17.3718369	44.3571928	39.5055842	4
chr4:87967318|88005316	0.0000000	0.0000000	0.0000000	0.5232528	5
chr4:87967318|88016121	0.2179968	0.4278778	0.0000000	0.1308132	7
chr4:88016067|88036451	0.0000000	0.6846044	0.0000000	0.1308132	7
chr4:88026780|88036451	0.5177424	0.5990289	0.9289464	1.4389451	4
chr4:88029311|88036451	0.4904928	0.2567267	0.0000000	0.2616264	6
chr4:88029311|88046295	0.4904928	0.0000000	0.0000000	0.0000000	6
chr4:88035519|88036451	0.7902384	0.2567267	0.0000000	0.3924396	6
chr4:88091231|88116842	0.0000000	0.4278778	0.6967098	0.0000000	3
chr4:88104355|88116842	0.0000000	0.7701800	0.0000000	0.0000000	7
chr4:88116476|88116842	0.0000000	0.0000000	0.6967098	0.9156923	4
chr5:100191807|100231489	0.0000000	0.5134533	0.0000000	0.0000000	7
chr5:102432245|102433485	0.9537359	0.0000000	0.0000000	0.0000000	6
chr5:10258223|10261023	0.0000000	0.0000000	0.4644732	0.0000000	2
chr5:10261024|10261630	0.1634976	0.0000000	0.4644732	0.1308132	1
chr5:1032328|1033417	0.4941442	0.0000000	0.0000000	0.0000000	6
chr5:107684100|107703654	0.1362480	0.3423022	0.2322366	0.0000000	3
chr5:112198281|112200129	0.0000000	0.0000000	0.9289464	0.0000000	2
chr5:113740135|113740553	7.1666444	1.5403599	5.5736787	2.4854507	1
chr5:122868263|122893258	0.4087440	0.0000000	0.0000000	0.3924396	5
chr5:122881111|122881535	0.4359936	0.0000000	0.0000000	0.0000000	6
chr5:122881111|122888841	0.0000000	0.0855756	0.0000000	0.7848792	5

circRNA_coordinates	m6A-CTRL-S1	m6A-CTRL-S2	m6A-BTZ-S1	m6A-BTZ-S2	cluster_group
chr5:122881111|122893258	0.8992368	1.1980577	1.6256563	3.1395166	4
chr5:126140468|126147590	0.3269952	0.0000000	0.0000000	0.2068157	6
chr5:126140468|126168493	0.3542448	0.0855756	0.6967098	0.5232528	1
chr5:130515788|130522802	0.3542448	0.0000000	0.3910865	0.0000000	1
chr5:130766552|130769351	0.1089984	0.0855756	0.0000000	0.2616264	5
chr5:131052258|131080383	0.0000000	0.0087287	0.4644732	0.0000000	2
chr5:132232015|132235333	0.0000000	0.0000000	0.4644732	0.0000000	2
chr5:132232015|132240096	0.0000000	0.0000000	0.4644732	0.0000000	2
chr5:132262813|132272885	0.4904928	0.0000000	0.0000000	0.0000000	6
chr5:132400672|132406188	0.0662983	0.3389648	0.0000000	0.0000000	7
chr5:133534777|133541822	0.0000000	0.0000000	0.6967098	0.3488788	2
chr5:134076753|134079742	0.5994912	0.2567267	0.0000000	0.0000000	6
chr5:134116823|134121276	0.0000000	0.5134533	0.0000000	0.0000000	7
chr5:134670854|134678949	0.0000000	0.5134533	0.0000000	0.2616264	7
chr5:134670856|134678949	0.0000000	0.0000000	0.4644732	0.0000000	2
chr5:134679156|134681657	0.5994912	0.0000000	0.0000000	0.0000000	6
chr5:135483521|135489852	0.5177424	0.0000000	0.2322366	0.3924396	1
chr5:135489281|135489852	0.4087440	0.0000000	0.0000000	0.0000000	6
chr5:137278564|137278905	0.1634976	0.0000000	0.2064583	0.0523253	1
chr5:137320946|137324004	1.2807311	0.3423022	1.8578929	0.9156923	1
chr5:137320946|137354835	0.4087440	0.0000000	0.0000000	0.0000000	6
chr5:137342679|137356886	0.1089984	0.1711511	0.0000000	0.2616264	5
chr5:137593328|137600237	0.0000000	0.0000000	0.4644732	0.0000000	2
chr5:137721711|137735701	0.4632432	0.3423022	1.1611831	0.0000000	2
chr5:137893091|137903411	0.6539904	0.0000000	0.0000000	0.0000000	6

circRNA_coordinates	m6A-CTRL-S1	m6A-CTRL-S2	m6A-BTZ-S1	m6A-BTZ-S2	cluster_group
chr5:138699448|138700432	0.0000000	0.1711511	0.2933148	0.0000000	3
chr5:139815689|139844361	0.2179968	0.0000000	0.0000000	0.2616264	5
chr5:139819704|139825560	0.0000000	0.1506130	0.0000000	0.3737333	5
chr5:142281500|142283234	0.0000000	0.0000000	1.7029911	0.0000000	2
chr5:142779221|142780417	4.1146894	2.4816910	3.0190760	3.0087034	1
chr5:149213342|149214207	0.0000000	0.0000000	0.4644732	0.0000000	2
chr5:149215826|149221940	0.4087440	0.0855756	0.4644732	0.0000000	1
chr5:149215826|149225477	1.2262319	0.3423022	1.6256563	0.0000000	1
chr5:151169884|151170623	0.0000000	0.0000000	0.3910865	0.7848792	4
chr5:154169885|154191200	0.4087440	0.0000000	0.4644732	0.5232528	1
chr5:167915607|167921655	0.5177424	0.0000000	0.0000000	0.0000000	6
chr5:167986037|167996003	3.7331950	0.5990289	3.0190760	1.1773187	1
chr5:167993018|167996003	1.2262319	0.1711511	0.0000000	0.5232528	6
chr5:167995651|167996003	2.5614623	1.7115110	0.6967098	2.0930111	7
chr5:169108748|169116337	0.1634976	0.4635628	1.4965327	0.0000000	2
chr5:169108748|169267856	0.0000000	0.0000000	0.4644732	0.0000000	2
chr5:171482592|171484477	0.1089984	0.0000000	0.1999557	0.6789205	4
chr5:175109712|175111312	0.5449920	0.4278778	0.6967098	0.0000000	3
chr5:175109712|175118362	0.0000000	0.0855756	0.6967098	0.0000000	2
chr5:175716657|175717958	1.0899839	0.1711511	0.4644732	0.7848792	1
chr5:175716657|175723323	0.0000000	0.7701800	1.3934197	0.0000000	3
chr5:175716657|175764149	0.0000000	0.0000000	0.6967098	0.0000000	2
chr5:175825067|175837257	0.0000000	0.0000000	0.6967098	0.0000000	2
chr5:176370336|176385155	0.5207671	0.0000000	0.0000000	0.0000000	6
chr5:176618885|176639196	0.2724960	0.2567267	0.2322366	0.0000000	3

circRNA_coordinates	m6A-CTRL-S1	m6A-CTRL-S2	m6A-BTZ-S1	m6A-BTZ-S2	cluster_group
chr5:176636637|176639196	2.2344671	0.9413311	0.6967098	1.3081319	6
chr5:176963359|176966148	0.2997456	0.2567267	0.0000000	0.0000000	7
chr5:177053437|177059739	1.9074719	0.9413311	0.9289464	1.4389451	6
chr5:178287129|178287975	0.0000000	0.0000000	0.0000000	0.5232528	5
chr5:178989548|178996426	0.7874316	0.0000000	0.0000000	0.0000000	6
chr5:179018626|179020646	0.0817488	0.0855756	0.6967098	0.1308132	2
chr5:179136874|179147561	1.5532271	0.0000000	0.2322366	0.0000000	6
chr5:179300275|179301901	0.0000000	0.0855756	0.0000000	0.3924396	5
chr5:180665271|180666066	1.6391451	0.0000000	0.0000000	0.0000000	6
chr5:32135678|32143986	0.2997456	0.2477412	0.0977716	0.7474666	5
chr5:353845|376831	0.1634976	0.5134533	0.2322366	0.0000000	7
chr5:36953720|36976504	1.2534815	0.0000000	0.0000000	0.3924396	6
chr5:36984778|36986403	0.0817488	0.0000000	0.0000000	0.3924396	5
chr5:38991051|39021238	0.0000000	0.0000000	0.5866297	0.0000000	2
chr5:39201928|39203089	0.2179968	0.0000000	0.6967098	0.0000000	1
chr5:43122141|43162033	0.0000000	0.0000000	0.0000000	0.5232528	5
chr5:43161351|43162033	0.0000000	0.3423022	0.0000000	0.1308132	7
chr5:43675613|43677908	0.0000000	0.1564321	0.3910865	0.1569758	2
chr5:49698120|49707217	0.0000000	0.0855756	0.2322366	0.3924396	4
chr5:50045985|50059076	0.5036816	0.0173718	0.6967098	0.0000000	1
chr5:50073903|50093067	0.2997456	1.6259355	1.6256563	1.1773187	2
chr5:50084357|50093067	0.4904928	0.1711511	0.6967098	0.7848792	4
chr5:5441225|5457854	0.0000000	0.0000000	0.4644732	0.0000000	2
chr5:60768508|60768864	0.0000000	0.5990289	0.6967098	0.3924396	2
chr5:60768508|60790237	0.4359936	0.0000000	0.6967098	0.5232528	1

circRNA_coordinates	m6A-CTRL-S1	m6A-CTRL-S2	m6A-BTZ-S1	m6A-BTZ-S2	cluster_group
chr5:619105|648031	0.4904928	0.3423022	0.0000000	0.2616264	7
chr5:619105|655699	0.0000000	0.1711511	0.0000000	0.5232528	5
chr5:64863340|64868113	1.3079807	1.0269066	0.6967098	0.9156923	6
chr5:65284463|65290692	0.1634976	0.2567267	0.0000000	0.5232528	5
chr5:65349234|65350779	0.1089984	1.0269066	0.9289464	1.8313847	5
chr5:6604251|6605521	0.3542448	0.0000000	0.0000000	0.0996797	6
chr5:6737623|6746540	0.0000000	0.0299514	0.9289464	0.0000000	2
chr5:67522118|67522837	0.1089984	0.1711511	0.0000000	0.2616264	5
chr5:68470704|68471364	2.3707151	0.5134533	0.9289464	1.1773187	1
chr5:68590620|68609870	0.0000000	0.0000000	0.0000000	0.7474666	5
chr5:70848943|70856064	0.0000000	0.0000000	0.4644732	0.0000000	2
chr5:73930509|73932323	0.4359936	0.0000000	0.0000000	0.0000000	6
chr5:74981032|74998635	0.0000000	0.0000000	1.1611831	0.1308132	2
chr5:75902004|75936928	0.5722416	0.0000000	0.0000000	0.0000000	6
chr5:76758920|76760634	0.1634976	0.0000000	0.6967098	0.2616264	1
chr5:77385217|77425131	0.4632432	0.0855756	0.0000000	0.7848792	5
chr5:77684661|77714727	0.0000000	0.0000000	0.6967098	0.0000000	2
chr5:77805607|77806221	0.3814944	0.0000000	0.4644732	0.5232528	1
chr5:78251118|78281381	1.7167247	0.8557555	0.0000000	0.5232528	6
chr5:87566343|87583439	0.0605486	0.0000000	0.1955432	0.6540660	4
chr5:94204038|94230540	0.0000000	0.0000000	0.4129167	0.0000000	2
chr5:94204038|94259726	0.2997456	0.0000000	0.0000000	0.6540660	5
chr5:96314842|96322374	0.8174880	0.2567267	0.0000000	0.0000000	6
chr6:101214441|101296583	0.1634976	0.0000000	0.6967098	0.3924396	2
chr6:101246589|101296583	0.0000000	0.6846044	0.0000000	0.0000000	7

circRNA_coordinates	m6A-CTRL-S1	m6A-CTRL-S2	m6A-BTZ-S1	m6A-BTZ-S2	cluster_group
chr6:101296024|101296583	0.1907472	0.0000000	0.0000000	0.7848792	5
chr6:105192035|105233194	0.9264863	0.0000000	0.0000000	0.0000000	6
chr6:10702602|10702872	0.2997456	0.0000000	0.2322366	0.6540660	4
chr6:10703638|10705077	0.2862570	0.0000000	0.4644732	0.0000000	1
chr6:108225833|108246136	0.0000000	0.3423022	0.4644732	0.0000000	3
chr6:108984658|108986092	3.8694430	1.4547844	1.8578929	0.0000000	6
chr6:10935291|10956475	0.0000000	0.0000000	0.6967098	0.0000000	2
chr6:109962721|109993370	0.4087440	0.0000000	0.0000000	0.0000000	6
chr6:110036281|110064975	0.5722416	0.0000000	0.0000000	0.0000000	6
chr6:110048312|110064975	0.1634976	0.1711511	0.0000000	0.2616264	5
chr6:111208708|111211559	0.3754450	0.0000000	0.0000000	0.0523253	6
chr6:111708968|111714175	0.4087440	0.0000000	0.0000000	0.0000000	6
chr6:118832461|118887479	0.0000000	0.0000000	0.4644732	0.0000000	2
chr6:126332399|126360071	0.0000000	0.0000000	0.4644732	0.0000000	2
chr6:127635961|127652175	0.0000000	0.0000000	0.4644732	0.0000000	2
chr6:130343372|130425720	0.0000000	0.2567267	0.2322366	0.0000000	3
chr6:135507041|135524462	0.0000000	0.0000000	0.0000000	0.5232528	5
chr6:135513462|135524462	0.0000000	0.5134533	0.0000000	0.0000000	7
chr6:13632602|13644961	0.4087440	0.8557555	1.8578929	1.1773187	2
chr6:13639795|13644961	0.4230500	0.0000000	1.3934197	0.0000000	1
chr6:137015278|137019820	0.0783426	0.0000000	0.6967098	0.0000000	2
chr6:139487421|139488461	0.7084896	0.0000000	0.0000000	0.2616264	6
chr6:144086398|144086935	1.2262319	0.2567267	0.0000000	0.3924396	6
chr6:144808684|144814592	0.3269952	0.0000000	0.4644732	0.0523253	1
chr6:144811207|144814592	0.0000000	0.0000000	0.6967098	0.0000000	2

circRNA_coordinates	m6A-CTRL-S1	m6A-CTRL-S2	m6A-BTZ-S1	m6A-BTZ-S2	cluster_group
chr6:146125508|146127557	0.4632432	0.0000000	0.0000000	0.0000000	6
chr6:146209156|146216113	0.0000000	0.0000000	1.3934197	0.0000000	2
chr6:146234614|146243484	0.0000000	0.0000000	0.4644732	0.0000000	2
chr6:149691045|149700654	0.3814944	0.0000000	0.6967098	1.4389451	4
chr6:149997396|150001593	2.3434655	1.1980577	1.6256563	0.0000000	6
chr6:150001011|150001593	0.0000000	0.0000000	0.0000000	0.7848792	5
chr6:150016210|150023402	0.4359936	0.1711511	0.0000000	0.0000000	6
chr6:151330955|151358253	2.2538143	0.0000000	0.0000000	0.0000000	6
chr6:15496363|15497401	0.6267408	0.6846044	0.6967098	0.0000000	3
chr6:155095123|155116273	0.0000000	0.0299514	0.0000000	0.7848792	5
chr6:155108995|155114110	0.0000000	0.5990289	0.0000000	0.0000000	7
chr6:157150361|157222659	1.1285966	0.0000000	0.0000000	0.0000000	6
chr6:158994452|159010814	0.0000000	0.0000000	0.2322366	0.7848792	4
chr6:159004986|159010814	0.5722416	0.0000000	0.0000000	0.0000000	6
chr6:160205698|160209175	0.4087440	0.0000000	0.0000000	0.0000000	6
chr6:160467530|160469575	0.2179968	0.0855756	0.0000000	0.3924396	5
chr6:161455291|161471011	1.6349759	0.3423022	1.1611831	0.0000000	6
chr6:161469648|161471011	0.8174880	0.2567267	0.0000000	0.0000000	6
chr6:16326625|16328701	4.9866765	3.5941732	9.2894645	5.1017145	1
chr6:163876311|163899928	0.0000000	0.2567267	0.0000000	0.3924396	5
chr6:16586011|16753578	0.0000000	0.0000000	0.5160298	0.0000000	2
chr6:167435897|167447459	0.0000000	0.0000000	0.6967098	0.0000000	2
chr6:169982932|170049371	0.0000000	0.0000000	0.4644732	0.0000000	2
chr6:170058372|170064382	0.4901385	0.0000000	0.0000000	0.2616264	6
chr6:170626458|170628212	0.5994912	0.0000000	0.0000000	0.0000000	6

circRNA_coordinates	m6A-CTRL-S1	m6A-CTRL-S2	m6A-BTZ-S1	m6A-BTZ-S2	cluster_group
chr6:170626458|170632347	0.0000000	2.2249643	0.0000000	1.0465055	7
chr6:170626458|170639638	0.1362480	0.9413311	1.8578929	1.1773187	2
chr6:170632167|170639638	0.0000000	0.0000000	5.5736787	0.0000000	2
chr6:17624792|17640295	0.0000000	0.5990289	0.0000000	0.0000000	7
chr6:17624792|17649531	1.1717327	1.2836333	0.6967098	0.0000000	3
chr6:17632881|17640295	0.0000000	0.0000000	0.4644732	0.1308132	2
chr6:17637384|17640295	0.4359936	0.0000000	0.0000000	0.2616264	6
chr6:17665470|17669259	0.1490553	0.4056281	0.1549018	0.3924396	5
chr6:18160114|18166609	0.0000000	0.2567267	0.0000000	1.1773187	5
chr6:20480077|20488479	0.0363237	0.8557555	0.0000000	0.0000000	7
chr6:20546577|20548936	0.4359936	0.0000000	0.0000000	0.0000000	6
chr6:20781376|21108694	0.0000000	0.0000000	0.4644732	0.0000000	2
chr6:24416625|24418806	0.3269952	0.0000000	0.0977716	0.0000000	6
chr6:25097289|25106757	0.0272496	0.0000000	0.9289464	0.1308132	2
chr6:28327302|28329248	0.4904928	0.0000000	0.0000000	0.0000000	6
chr6:30638187|30639051	0.4087440	0.0000000	0.9289464	0.5232528	1
chr6:30679189|30682955	0.3542448	0.0855756	0.0000000	0.0000000	6
chr6:32822067|32823889	0.0000000	0.0000000	0.6967098	0.0000000	2
chr6:33658887|33659368	0.2452464	0.0000000	0.2322366	0.0000000	1
chr6:34574332|34574681	0.3542448	0.0000000	0.6967098	0.1308132	1
chr6:34574332|34614575	0.6812400	0.8557555	0.2322366	0.9156923	7
chr6:35586873|35610620	0.0000000	0.4278778	0.0000000	0.0622671	7
chr6:35825038|35840505	0.6539904	0.0855756	0.0000000	0.1308132	6
chr6:36685197|36685768	0.0000000	0.0000000	0.0000000	0.7848792	5
chr6:37336154|37339350	0.8174880	0.0000000	0.6967098	0.0000000	1

circRNA_coordinates	m6A-CTRL-S1	m6A-CTRL-S2	m6A-BTZ-S1	m6A-BTZ-S2	cluster_group
chr6:37336260|37339350	0.4087440	0.1711511	0.0000000	0.1308132	6
chr6:41748565|41749048	0.0000000	0.0000000	0.4644732	0.1569758	2
chr6:42235892|42237586	0.9537359	0.3423022	1.1611831	0.0000000	1
chr6:42541472|42562042	0.0000000	0.0000000	0.4644732	0.0000000	2
chr6:42559889|42562042	0.0000000	0.3423022	0.0000000	0.1308132	7
chr6:42571326|42574389	0.0000000	0.0000000	0.6967098	0.1569758	2
chr6:42941741|42942776	0.6825752	0.0000000	0.0000000	0.0000000	6
chr6:42993880|42994886	0.5722416	0.0000000	0.0000000	0.0000000	6
chr6:43501644|43519190	0.1089984	0.4278778	0.0000000	0.0000000	7
chr6:43555009|43568828	0.0000000	0.4301883	0.0000000	0.0000000	7
chr6:43592821|43593099	0.0000000	0.0000000	0.4644732	1.0465055	4
chr6:43592823|43593099	0.2997456	0.2503085	0.0000000	0.1308132	7
chr6:43592826|43593099	0.0000000	0.0000000	0.0000000	0.6166534	5
chr6:47251674|47254331	0.2452464	0.2567267	0.0000000	0.0000000	7
chr6:4891947|4892613	136.7384854	51.6876331	106.8288413	131.7288849	1
chr6:4891947|4937971	0.0000000	0.0000000	0.6967098	0.1308132	2
chr6:52132663|52134024	1.2807311	0.3042211	0.0000000	0.3924396	6
chr6:5368783|5369415	1.1989823	0.3423022	0.4644732	0.0000000	6
chr6:5368783|5431405	0.4359936	0.0000000	0.0000000	0.0000000	6
chr6:7176888|7189555	1.7167247	1.7970866	3.9480224	2.4854507	2
chr6:7176888|7211942	0.0000000	0.0000000	0.4644732	0.1308132	2
chr6:7181357|7189555	0.6267408	0.3423022	1.1611831	0.1308132	1
chr6:7187667|7232140	0.0000000	0.1711511	0.2322366	0.0000000	3
chr6:7402836|7411684	0.0000000	0.0000000	0.4644732	0.0000000	2
chr6:74175932|74176329	0.2452464	0.2086332	0.0000000	0.1308132	7

circRNA_coordinates	m6A-CTRL-S1	m6A-CTRL-S2	m6A-BTZ-S1	m6A-BTZ-S2	cluster_group
chr6:7602856|7606428	0.5722416	0.0000000	0.0000000	0.0000000	6
chr6:76344423|76373234	0.4904928	0.0000000	0.6967098	0.1308132	1
chr6:76344423|76380436	0.1089984	0.3423022	0.0000000	0.1308132	7
chr6:76368978|76380436	0.0000000	0.0855756	0.0000000	0.5232528	5
chr6:76372937|76380436	0.0000000	0.0119806	0.4644732	0.0000000	2
chr6:76412361|76412788	1.5532271	0.2567267	0.4644732	0.6540660	1
chr6:76412361|76421132	1.3079807	0.5134533	0.0000000	0.0000000	6
chr6:79655017|79664999	0.0000000	0.0000000	0.4644732	0.0000000	2
chr6:79752560|79770535	0.9809855	0.2567267	0.0859275	0.2616264	6
chr6:82923944|82924546	0.0000000	0.1711511	0.9289464	0.1308132	2
chr6:84894905|84896341	0.0000000	0.4278778	0.4644732	0.0000000	3
chr6:88058530|88060219	0.0000000	0.0000000	0.0000000	0.6540660	5
chr6:88224146|88231242	0.4632432	0.0000000	0.0000000	0.0000000	6
chr6:89554076|89614674	0.4087440	0.0000000	0.0000000	0.0000000	6
chr6:90052043|90053475	0.0000000	0.4171808	0.0000000	0.0000000	7
chr6:90400392|90403902	0.2724960	0.0000000	0.0000000	0.1308132	6
chr6:90481280|90484452	0.4904928	0.0000000	0.0000000	0.0000000	6
chr6:99347144|99365595	0.2724960	0.4278778	0.0000000	0.0000000	7
chr6:99887645|99913012	0.0000000	0.2567267	0.0000000	0.3924396	5
chr6_cox_hap2:4383910|4385242	0.3542448	0.7701800	0.6967098	0.1308132	3
chr6_dbb_hap3:4568350|4568481	0.0000000	0.0000000	0.0000000	0.9156923	5
chr6_mcf_hap5:3314468|3314600	0.0000000	0.0000000	0.4888581	0.0000000	2
chr6_mcf_hap5:3468376|3473857	0.0000000	0.0000000	0.0000000	0.9343986	5
chr6_qbl_hap6:2838232|2838501	0.0000000	0.2567267	0.1955432	0.0000000	3
chr7:100282796|100282966	0.4359936	0.0000000	0.0000000	0.0000000	6

circRNA_coordinates	m6A-CTRL-S1	m6A-CTRL-S2	m6A-BTZ-S1	m6A-BTZ-S2	cluster_group
chr7:100410369|100410830	0.0817488	0.0855756	0.0000000	0.5232528	5
chr7:101801840|101821937	0.4087440	0.1711511	0.2322366	0.1308132	6
chr7:101801840|101848450	0.0000000	0.0000000	0.4644732	0.0000000	2
chr7:101870647|101870949	0.5881553	0.2567267	0.2322366	0.3924396	6
chr7:102755507|102769239	0.4632432	0.4278778	0.0000000	0.2616264	7
chr7:102962379|102963241	0.0000000	0.6846044	0.2322366	0.1046506	7
chr7:105103068|105108910	7.0778107	0.0000000	0.0000000	0.0000000	6
chr7:11080299|11101712	0.0000000	0.4278778	0.0000000	0.1308132	7
chr7:11101418|11151094	0.6539904	0.0000000	0.2322366	0.0000000	6
chr7:115889074|115892530	0.0000000	0.0000000	0.2322366	0.5232528	4
chr7:127013396|127017383	0.0000000	0.0855756	0.9289464	0.1308132	2
chr7:128655033|128658211	1.0899839	0.5134533	2.5546027	1.9621979	4
chr7:129297183|129367205	0.0553712	0.0000000	0.0000000	0.3924396	5
chr7:129679304|129688984	0.1210972	0.0299514	0.2933148	0.0000000	1
chr7:129756285|129762042	0.0000000	0.0000000	0.4644732	0.0000000	2
chr7:129760589|129762042	0.0000000	0.0000000	0.4644732	0.0000000	2
chr7:131113792|131130662	0.5177424	0.0000000	0.0000000	0.3924396	6
chr7:135078670|135082978	0.0000000	0.1711511	0.9289464	0.1308132	2
chr7:138758602|138774505	0.1634976	0.1711511	0.9289464	0.1308132	2
chr7:138763298|138764989	0.3269952	0.4953969	0.4644732	0.2616264	3
chr7:138921751|138951149	0.0000000	0.6846044	0.0000000	0.2616264	7
chr7:138943234|138957186	0.0000000	0.0000000	0.6967098	0.0000000	2
chr7:139415731|139416814	0.2179968	0.0000000	0.0000000	0.5232528	5
chr7:140439612|140508795	0.1907472	0.0000000	0.2322366	0.0000000	1
chr7:140476712|140508795	0.4904928	0.4278778	0.0000000	0.0000000	7

circRNA_coordinates	m6A-CTRL-S1	m6A-CTRL-S2	m6A-BTZ-S1	m6A-BTZ-S2	cluster_group
chr7:140534409|140534672	0.9400839	0.0000000	0.0000000	0.0000000	6
chr7:148543562|148544397	0.2543205	0.2086332	0.2322366	0.1308132	6
chr7:148711996|148718239	0.2148358	0.0000000	0.2322366	0.0000000	1
chr7:148716084|148718239	1.3624799	0.2567267	0.0000000	0.9156923	6
chr7:148851037|148851432	2.9157070	1.3692088	0.0000000	1.1773187	6
chr7:151935792|151948051	0.0000000	0.3423022	0.0000000	0.5232528	5
chr7:152007051|152012423	0.7084896	0.0652086	0.0000000	0.2616264	6
chr7:155457869|155473602	0.0272496	0.5990289	0.2322366	3.7935826	5
chr7:155457869|155477746	0.9264863	0.0855756	0.4129167	0.1308132	6
chr7:155465561|155473602	15.6685192	2.2249643	9.0572279	7.9796047	1
chr7:155471302|155473602	0.2179968	0.2567267	0.0000000	0.1777751	7
chr7:155499554|155511137	0.1907472	0.4278778	0.0000000	0.6540660	5
chr7:155499554|155538296	0.0000000	0.2567267	0.2322366	0.0000000	3
chr7:155503867|155511137	0.6658984	0.2567267	0.0000000	0.0000000	6
chr7:155530224|155538296	0.4087440	0.6846044	1.6256563	0.0000000	2
chr7:156956504|157000629	0.0000000	0.0000000	0.4644732	0.0000000	2
chr7:157159196|157160177	0.0000000	0.0000000	1.1611831	0.1869321	2
chr7:158448179|158448951	0.0000000	0.0000000	0.4644732	0.0000000	2
chr7:158552177|158557544	1.0082351	0.0855756	0.6967098	1.0465055	1
chr7:158580695|158591763	0.6267408	0.0000000	0.6967098	0.6540660	1
chr7:18535885|18706099	0.0000000	0.0000000	0.0000000	0.7848792	5
chr7:18624904|18688306	0.0000000	0.0000000	0.4644732	0.0000000	2
chr7:18668973|18706099	0.0000000	0.0000000	0.4644732	0.0000000	2
chr7:18674250|18688306	0.0000000	0.0000000	0.0000000	0.6727722	5
chr7:18705836|18706099	0.5722416	0.0000000	0.4644732	0.3924396	1

circRNA_coordinates	m6A-CTRL-S1	m6A-CTRL-S2	m6A-BTZ-S1	m6A-BTZ-S2	cluster_group
chr7:1937836|1976533	0.0000000	0.0000000	0.0000000	0.5232528	5
chr7:1937836|2108973	0.1634976	0.0000000	0.6967098	0.0000000	2
chr7:1976323|1997354	0.0000000	0.0000000	0.6604809	0.0000000	2
chr7:21943728|21948125	0.0000000	0.0000000	0.4644732	0.0000000	2
chr7:2252847|2259091	0.5177424	0.1711511	0.0000000	0.1370922	6
chr7:2252847|2262389	0.2452464	0.0000000	0.4644732	0.0000000	1
chr7:2252847|2265185	0.6539904	0.0000000	0.0000000	0.3924396	6
chr7:2262210|2270359	0.4287452	0.0000000	0.0000000	0.0000000	6
chr7:2269619|2270362	0.8719872	0.0000000	0.4024660	0.0000000	6
chr7:23224689|23226765	0.0000000	0.0000000	0.6193750	0.2616264	2
chr7:2403267|2404164	0.3796414	0.0490348	0.0000000	0.0000000	6
chr7:24659671|24708279	0.3535363	0.2567267	0.0000000	0.9156923	5
chr7:26232115|26237352	0.0000000	0.0000000	0.4644732	0.0000000	2
chr7:26724355|26729981	0.0000000	0.0000000	0.6967098	0.5232528	4
chr7:27668990|27689252	0.4359936	0.0000000	0.0000000	0.0000000	6
chr7:30590252|30601744	0.8992368	0.2567267	0.6967098	1.7005715	4
chr7:32582753|32599076	0.0000000	0.6846044	0.0000000	0.5232528	5
chr7:32582753|32609766	0.2179968	0.2567267	0.2322366	0.0000000	3
chr7:40027198|40027857	0.4359936	0.6846044	0.6967098	1.4389451	4
chr7:40027198|40041630	0.0000000	1.0269066	0.9289464	0.0000000	3
chr7:40037093|40087476	0.0000000	0.0000000	0.4644732	0.0000000	2
chr7:47407962|47409218	1.9074719	0.5134533	0.0000000	0.7848792	6
chr7:4780469|4780654	0.0000000	0.0000000	0.0000000	0.5762321	5
chr7:4795015|4796597	0.0000000	0.0855756	0.4644732	0.0000000	2
chr7:50358644|50367353	0.4087440	0.0000000	0.0000000	0.0000000	6

circRNA_coordinates	m6A-CTRL-S1	m6A-CTRL-S2	m6A-BTZ-S1	m6A-BTZ-S2	cluster_group
chr7:5352134|5353494	0.2452464	0.5990289	0.0000000	0.5232528	5
chr7:5391450|5399200	0.1634976	0.0000000	0.2322366	0.1308132	1
chr7:5410006|5410995	1.7167247	0.0000000	0.0000000	0.0000000	6
chr7:5680785|5681007	0.5544203	0.0000000	0.0000000	0.1951733	6
chr7:5778907|5781275	0.0000000	0.0000000	0.0000000	0.5232528	5
chr7:5778907|5781446	0.2997456	0.9413311	0.4644732	0.6540660	7
chr7:5780604|5781446	0.0000000	0.3423022	0.4644732	0.0000000	3
chr7:6026390|6027251	0.0000000	0.0000000	0.4644732	0.0000000	2
chr7:6068246|6068663	0.0000000	0.0000000	0.7097151	0.0000000	2
chr7:6187370|6190137	0.0000000	0.0000000	0.4644732	0.0000000	2
chr7:64004085|64004810	0.2997456	0.1711511	1.1611831	0.6540660	2
chr7:65705312|65706257	0.0000000	0.0000000	0.0000000	1.0465055	5
chr7:66456124|66459328	0.4904928	0.0000000	0.0000000	0.1308132	6
chr7:66458204|66459328	0.4087440	0.0299514	0.0000000	0.0000000	6
chr7:6776707|6777567	0.3814944	0.2567267	0.0000000	0.0000000	7
chr7:72302182|72302378	0.0000000	0.1497572	0.4644732	0.0000000	2
chr7:72861594|72884813	0.0000000	0.0000000	0.6967098	0.0000000	2
chr7:72873866|72884813	0.8174880	0.5990289	0.9289464	1.1773187	4
chr7:73100966|73101425	0.0000000	0.0000000	0.0000000	0.6540660	5
chr7:73654268|73663448	0.4087440	0.0000000	0.0000000	0.0000000	6
chr7:73752778|73753334	0.0000000	0.0000000	0.0000000	0.5232528	5
chr7:75048757|75050791	0.7357392	0.5990289	0.0000000	0.0000000	7
chr7:77200395|77230123	0.2179968	0.2567267	0.0000000	0.0000000	7
chr7:77214860|77230123	1.4161616	0.0000000	0.5866297	0.0000000	6
chr7:91924203|91936970	0.2179968	0.1711511	0.4644732	0.6540660	4

circRNA_coordinates	m6A-CTRL-S1	m6A-CTRL-S2	m6A-BTZ-S1	m6A-BTZ-S2	cluster_group
chr7:91924203|91957214	0.1634976	0.5134533	3.0190760	0.0000000	2
chr7:97820040|97823884	0.9264863	0.5134533	0.9289464	0.5232528	1
chr7:98930948|98935908	0.0000000	0.0035086	0.6967098	0.0000000	2
chr7:99001021|99002576	0.0000000	0.1760289	0.4644732	0.6540660	4
chr7:99123436|99124041	0.1089984	0.2567267	1.3934197	0.0000000	2
chr7:99616854|99621930	0.0000000	0.0000000	0.6967098	0.1308132	2
chr7:99621042|99621930	40.5746522	15.1468726	50.8598179	38.0666390	1
chr7:99621042|99627998	0.4087440	0.1711511	0.0000000	0.0000000	6
chr7:99717486|99719840	0.0000000	0.0000000	0.4644732	0.0000000	2
chr8:100108540|100133673	0.2452464	0.2567267	0.6967098	0.1308132	2
chr8:100108540|100205285	0.0000000	0.1711511	0.0000000	0.5232528	5
chr8:100568678|100733275	0.0000000	0.0000000	0.6967098	0.0000000	2
chr8:101299729|101300495	0.5722416	0.2567267	0.0000000	1.3081319	5
chr8:102211423|102213971	0.2711880	0.0000000	0.0000000	0.9156923	5
chr8:102731480|103032517	0.4359936	0.0000000	0.0000000	0.0000000	6
chr8:103299661|103300494	0.0440081	0.1711511	0.2322366	0.0000000	3
chr8:103372299|103373854	0.1907472	0.2824849	0.3910865	0.2093011	2
chr8:103846417|103852051	0.0000000	0.0504040	0.4644732	0.1308132	2
chr8:104417004|104420012	1.6077263	0.2567267	0.0000000	0.7848792	6
chr8:107715134|107726213	0.2997456	0.0000000	0.4644732	0.0000000	1
chr8:109462052|109462721	0.2997456	0.6846044	0.0000000	0.0000000	7
chr8:109462052|109468159	0.0544992	0.3423022	0.0977716	0.2616264	5
chr8:117668095|117671219	0.2724960	0.0000000	0.0000000	0.3924396	5
chr8:117861185|117869010	0.4087440	0.0000000	0.0000000	0.0000000	6
chr8:124238740|124243997	0.0000000	0.0000000	0.6967098	0.0000000	2

circRNA_coordinates	m6A-CTRL-S1	m6A-CTRL-S2	m6A-BTZ-S1	m6A-BTZ-S2	cluster_group
chr8:124238740|124251384	0.7084896	0.0072739	0.0000000	0.7848792	5
chr8:124243540|124243997	0.0000000	0.4278778	0.0000000	0.7848792	5
chr8:124243540|124251384	1.0899839	0.8557555	1.3934197	0.0000000	3
chr8:124335178|124351686	0.0000000	0.1711511	0.9289464	0.0000000	2
chr8:124349865|124351686	0.2179968	0.0000000	0.0000000	0.3924396	5
chr8:124368629|124369976	0.0000000	0.0000000	0.4644732	0.0000000	2
chr8:125332327|125343033	0.6658984	0.0000000	0.0000000	0.1046506	6
chr8:126015418|126019729	0.0000000	0.0000000	0.6967098	0.0000000	2
chr8:126114256|126194498	1.0003600	0.0000000	0.0000000	0.0000000	6
chr8:128902835|128903244	6.1584093	1.7115110	4.6447322	4.1860222	1
chr8:130915558|130924761	0.3269952	0.0000000	0.0000000	0.1308132	6
chr8:131164982|131181313	0.5177424	0.0000000	0.0000000	0.0000000	6
chr8:131164982|131193126	0.5177424	0.8557555	3.2513126	0.0000000	2
chr8:132952746|132991668	0.7357392	0.0000000	0.0000000	0.0000000	6
chr8:132952746|132999949	0.1907472	0.0000000	0.0000000	0.2616264	5
chr8:135596075|135622898	0.4087440	0.0000000	0.2322366	0.0000000	6
chr8:135612679|135622898	3.7331950	2.1393888	3.9480224	2.6162639	1
chr8:141407719|141415797	0.0000000	0.0000000	0.5882553	0.0000000	2
chr8:141874411|141900868	0.0000000	0.1711511	0.4644732	0.1308132	2
chr8:141889570|141900868	0.7357392	0.0000000	0.0000000	0.0000000	6
chr8:142154247|142161052	0.0000000	0.0000000	0.6709316	0.0000000	2
chr8:142154247|142178624	0.3269952	0.0855756	0.0000000	0.0000000	6
chr8:142160933|142178624	0.2997456	0.0000000	0.9289464	0.0000000	1
chr8:142160933|142200516	0.0000000	0.0000000	0.6967098	0.0000000	2
chr8:142165948|142178624	0.5449920	0.0855756	0.2322366	0.0000000	6

circRNA_coordinates	m6A-CTRL-S1	m6A-CTRL-S2	m6A-BTZ-S1	m6A-BTZ-S2	cluster_group
chr8:142170731|142173528	0.3692866	0.0000000	0.7568591	0.1775135	1
chr8:142264088|142264728	161.6718680	52.6289641	103.3452921	126.3655440	1
chr8:143412262|143427253	0.4359936	0.0855756	0.6967098	0.1308132	1
chr8:143425327|143427253	2.1254687	0.5134533	0.2322366	2.2238243	5
chr8:144669047|144671160	0.0000000	0.0000000	0.4644732	0.0000000	2
chr8:145193717|145193894	0.0000000	0.0000000	0.6967098	0.0000000	2
chr8:145533141|145537717	0.3542448	0.0000000	0.0000000	0.2107401	6
chr8:145657867|145658970	0.4087440	0.2567267	0.0000000	0.1308132	6
chr8:145668213|145668520	0.9809855	0.0000000	0.0000000	0.3924396	6
chr8:145668215|145668520	0.0000000	0.7701800	0.4888581	1.1773187	5
chr8:145739120|145739311	0.1362480	0.3423022	0.2322366	0.0000000	3
chr8:19679950|19694671	0.0000000	0.0000000	0.6967098	0.0000000	2
chr8:27151597|27151827	0.2179968	0.5359597	0.4644732	0.1308132	3
chr8:27293866|27294606	0.0000000	0.0000000	0.4644732	0.0000000	2
chr8:27297959|27300352	0.0000000	0.4278778	0.0000000	0.0000000	7
chr8:28013459|28019595	0.4359936	0.2567267	0.0515565	0.2616264	6
chr8:28357450|28360719	0.4087440	0.0000000	0.2322366	0.2616264	1
chr8:28570974|28575724	0.8174880	0.7701800	0.0000000	0.0000000	7
chr8:29927158|29927575	0.4632432	0.0000000	1.1611831	0.5232528	1
chr8:30694319|30695577	0.9809855	0.3423022	0.4644732	0.0000000	6
chr8:30982038|31015046	0.0000000	0.0000000	0.4644732	0.0000000	2
chr8:33246481|33247316	0.2997456	0.7701800	0.0000000	0.0000000	7
chr8:37623044|37623873	0.7902384	0.4278778	0.6967098	0.6540660	1
chr8:37727938|37732840	0.8174880	0.0855756	0.0000000	0.0000000	6
chr8:37727938|37735069	13.1343065	7.8729507	17.8822191	20.1452317	4

circRNA_coordinates	m6A-CTRL-S1	m6A-CTRL-S2	m6A-BTZ-S1	m6A-BTZ-S2	cluster_group
chr8:37734627|37735069	1.0354847	0.4278778	0.4644732	0.6540660	6
chr8:37971710|37976881	0.5177424	0.0000000	0.2477965	0.1308132	1
chr8:37971710|37978667	0.2724960	0.0583625	0.2064583	1.1773187	5
chr8:37971710|37993284	0.1907472	0.0000000	0.4644732	0.0000000	1
chr8:37990972|37993284	0.0000000	0.1434246	0.1955432	0.2616264	5
chr8:38194823|38205733	0.0000000	0.0000000	0.0000000	1.1773187	5
chr8:38205015|38205733	1.7984735	0.0000000	2.7868393	2.0930111	1
chr8:38646222|38678153	0.8992368	0.0000000	0.0000000	0.2616264	6
chr8:38677040|38678153	0.5994912	0.6846044	0.0000000	0.0000000	7
chr8:41455811|41456823	0.8447376	0.6846044	0.0000000	0.0000000	7
chr8:41905896|41907225	2.6977103	1.8826621	2.3223661	2.3546375	1
chr8:42146247|42147653	0.0000000	0.0000000	0.6967098	0.0000000	2
chr8:42176194|42176762	0.0000000	0.0855756	0.2322366	0.7848792	4
chr8:42183488|42186732	0.0000000	0.0000000	0.8945754	0.0000000	2
chr8:42259306|42260979	0.0817488	0.0000000	1.3934197	0.0000000	2
chr8:42294507|42295095	0.3542448	0.2567267	0.0000000	0.0000000	7
chr8:42317414|42330172	0.8719872	0.2567267	0.6967098	0.6540660	1
chr8:42812237|42819617	0.0544992	0.0000000	2.6837263	0.0000000	2
chr8:43013718|43028886	0.0000000	0.0855756	0.0000000	0.5232528	5
chr8:48192450|48206619	0.0000000	0.5134533	0.6967098	0.0000000	3
chr8:48308936|48320523	0.8992368	0.7701800	0.2322366	1.5697583	5
chr8:48308936|48353104	0.5449920	0.0000000	0.0000000	0.0000000	6
chr8:48793977|48805947	0.3542448	0.0855756	0.0000000	0.0000000	6
chr8:48830837|48840450	0.0000000	0.0000000	0.6967098	0.0000000	2
chr8:48830837|48843347	0.0000000	0.0000000	0.0000000	1.0839181	5

circRNA_coordinates	m6A-CTRL-S1	m6A-CTRL-S2	m6A-BTZ-S1	m6A-BTZ-S2	cluster_group
chr8:48839754|48840450	0.5177424	0.0000000	0.0000000	0.2616264	6
chr8:48874076|48882617	0.0000000	0.4278778	0.0000000	0.0000000	7
chr8:48882358|48884028	0.6267408	0.1711511	0.4644732	0.3924396	1
chr8:48882358|48888408	0.4087440	0.0000000	0.2322366	0.0000000	6
chr8:52744004|52773806	0.7084896	0.4278778	0.2322366	0.9156923	5
chr8:52758221|52773806	0.0000000	0.7701800	0.0000000	0.5232528	5
chr8:52773405|52773806	4.0329406	8.3008285	8.3605180	16.6132755	5
chr8:52773421|52773806	0.0000000	0.5134533	0.4644732	0.6540660	5
chr8:56675194|56676539	0.4087440	0.0000000	0.0000000	0.3924396	5
chr8:56695307|56708711	0.0000000	0.5990289	0.0000000	0.9156923	5
chr8:56695307|56717595	0.0817488	0.5990289	0.0000000	0.0000000	7
chr8:56854414|56866543	0.0000000	0.0855756	0.2322366	0.1308132	2
chr8:59535779|59536339	0.0000000	0.0000000	0.4644732	0.0000000	2
chr8:61484605|61504528	0.3766440	0.0121517	0.2322366	0.0000000	6
chr8:61653818|61655656	2.5614623	2.3961154	3.2513126	1.4389451	3
chr8:618598|624047	1.0899839	0.0000000	0.0000000	0.6540660	6
chr8:62531537|62566219	0.3814944	0.4278778	0.0000000	0.5232528	7
chr8:62593527|62596747	2.8067086	0.6846044	0.0000000	1.3081319	6
chr8:640898|642612	0.0817488	0.0000000	0.0000000	0.5606653	5
chr8:64098706|64100303	0.8174880	1.4547844	0.6967098	1.0465055	7
chr8:67988717|68007967	1.1444831	0.0855756	0.6967098	0.0000000	6
chr8:68005778|68007967	2.8339582	0.6846044	0.2322366	1.0465055	6
chr8:68005778|68018210	0.0000000	0.0000000	0.0000000	0.5232528	5
chr8:68007528|68024300	0.5722416	0.0000000	0.0000000	0.0000000	6
chr8:68044186|68049838	0.3269952	0.1711511	0.0000000	0.3924396	5

circRNA_coordinates	m6A-CTRL-S1	m6A-CTRL-S2	m6A-BTZ-S1	m6A-BTZ-S2	cluster_group
chr8:68044186|68076743	0.0000000	0.2567267	0.6967098	0.0000000	2
chr8:68062018|68076743	0.3814944	0.2567267	0.0000000	1.0465055	5
chr8:68066259|68071372	0.3542448	0.2567267	0.0000000	0.0000000	7
chr8:68070682|68076743	0.0000000	0.0000000	0.9289464	0.0000000	2
chr8:71071740|71075089	0.3814944	0.0599029	0.2752004	0.0000000	6
chr8:71126138|71128999	0.1907472	0.3423022	0.2064583	0.1308132	7
chr8:74464247|74601048	0.0000000	0.2567267	0.4644732	0.0000000	3
chr8:74495005|74652103	0.1362480	0.0000000	0.4644732	0.0000000	1
chr8:74585342|74652103	0.0000000	0.3423022	0.4888581	0.0374126	3
chr8:80963762|80976828	0.6414011	0.0855756	0.2322366	0.0000000	6
chr8:87540764|87544808	0.0000000	0.0000000	0.3910865	0.0523253	2
chr8:97256147|97270928	0.6812400	0.1711511	0.0000000	0.0000000	6
chr8:97318672|97343323	0.0000000	0.0000000	2.4082937	0.0000000	2
chr8:98673300|98703416	0.5994912	0.0000000	0.0000000	0.0000000	6
chr8:98698896|98703416	0.0272496	0.1711511	0.0000000	0.2616264	5
chr8:98731277|98735263	0.0000000	0.4389170	0.0000000	0.0000000	7
chr8:99135564|99142454	0.3542448	0.0000000	0.0000000	0.2616264	6
chr8:99146177|99149182	0.2179968	0.0000000	0.0000000	0.2616264	5
chr8:99538970|99560389	0.4904928	0.0000000	0.0000000	0.0000000	6
chr8:99718695|99719539	0.2724960	0.3423022	0.6967098	0.0000000	3
chr9:100837852|100839299	0.0000000	0.0000000	0.4644732	0.0000000	2
chr9:100846774|100849723	0.0000000	0.0000000	0.4644732	0.0000000	2
chr9:111812563|111826847	0.3542448	0.0000000	0.0000000	0.1308132	6
chr9:112898407|112900819	0.9264863	0.8557555	0.6967098	0.2616264	3
chr9:113703701|113704448	0.0000000	0.0000000	0.9289464	0.0000000	2

circRNA_coordinates	m6A-CTRL-S1	m6A-CTRL-S2	m6A-BTZ-S1	m6A-BTZ-S2	cluster_group
chr9:113703701|113773970	0.4087440	0.0000000	0.0000000	0.0000000	6
chr9:113734353|113735838	0.0968996	0.0000000	0.0977716	0.2616264	4
chr9:114145491|114154104	2.9001203	0.0000000	0.0000000	0.0000000	6
chr9:114860750|114919893	0.2179968	0.0000000	0.6967098	0.0000000	1
chr9:114904600|114919893	0.2179968	0.0000000	0.4644732	0.0000000	1
chr9:115336337|115337531	0.0817488	0.2567267	0.6967098	0.0000000	2
chr9:117110004|117113301	0.0000000	0.0000000	0.4644732	0.0000000	2
chr9:123182066|123185019	0.4519618	0.0000000	0.0000000	0.0000000	6
chr9:123199573|123202250	0.5177424	0.0000000	0.0000000	0.0000000	6
chr9:123199573|123210404	0.2179968	0.1711511	0.0000000	0.3924396	5
chr9:123210173|123234156	0.0000000	0.0000000	0.4644732	0.0000000	2
chr9:123230138|123253755	0.1089984	0.0000000	0.4644732	0.0000000	2
chr9:123280705|123292418	0.1907472	0.3423022	0.0000000	0.2616264	7
chr9:123924134|123928458	0.1089984	0.0000000	0.2322366	1.1773187	4
chr9:125895124|125946577	0.0000000	0.1711511	0.2322366	0.1308132	2
chr9:125941286|125946577	0.1089984	0.2567267	0.0000000	0.0523253	7
chr9:127670656|127674305	0.1574482	0.0000000	0.0000000	0.5232528	5
chr9:128099297|128099870	0.1634976	0.0000000	0.4644732	0.1569758	1
chr9:128246722|128268696	0.0000000	0.0000000	0.4644732	0.0000000	2
chr9:128419930|128434922	0.7357392	0.0000000	0.0000000	0.0000000	6
chr9:128677965|128697886	0.0000000	0.0652086	0.4644732	0.2616264	2
chr9:131271155|131277918	0.1362480	0.0000000	1.6772128	0.0000000	2
chr9:132719639|132720832	0.0000000	0.0000000	0.4816587	0.3924396	4
chr9:134038378|134064518	0.0000000	0.0000000	2.1674643	0.0000000	2
chr9:135172274|135187243	0.2621411	0.0599029	0.0000000	0.1308132	6

circRNA_coordinates	m6A-CTRL-S1	m6A-CTRL-S2	m6A-BTZ-S1	m6A-BTZ-S2	cluster_group
chr9:135493717|135507478	0.0000000	0.0000000	0.4644732	0.0000000	2
chr9:136277419|136278041	0.2724960	0.0000000	0.0000000	0.2616264	5
chr9:136656905|136662931	0.0000000	0.0000000	0.0000000	1.0839181	5
chr9:136901154|136905391	0.0000000	0.0000000	0.4644732	0.0000000	2
chr9:138712685|138715106	0.5722416	0.4278778	0.0000000	0.0000000	7
chr9:138758302|138774924	0.4359936	0.0000000	0.0000000	0.0000000	6
chr9:138773479|138774924	6.7306508	2.2249643	5.1092055	3.1395166	1
chr9:139301597|139303519	0.0000000	0.0000000	0.6967098	0.0000000	2
chr9:139360414|139362964	0.8638940	0.0000000	0.0000000	0.0000000	6
chr9:140087163|140093438	1.0082351	0.0000000	0.0000000	0.0000000	6
chr9:140611078|140611634	1.7984735	0.0000000	0.0000000	0.2616264	6
chr9:140611078|140712590	0.2452464	0.1711511	0.0000000	0.0000000	7
chr9:140622801|140638542	0.0272496	0.0000000	0.6307546	0.0306103	2
chr9:140622801|140657272	0.0000000	0.1711511	0.0000000	0.3924396	5
chr9:140637823|140648743	0.0000000	0.0017115	0.4644732	0.0000000	2
chr9:140637823|140712590	0.0272496	0.0000000	0.4644732	0.2616264	2
chr9:17388160|17395067	0.0000000	0.1711511	0.0000000	0.5232528	5
chr9:17394532|17395067	0.1634976	0.2567267	0.0000000	0.0000000	7
chr9:20819795|20823114	0.3542448	0.0000000	0.0000000	0.1495195	6
chr9:22046750|22097363	0.4359936	0.0898543	0.0000000	0.0000000	6
chr9:26907832|26910437	0.0000000	0.0000000	0.6967098	0.0000000	2
chr9:271627|289581	0.0000000	0.4278778	0.0441250	0.0000000	7
chr9:271627|340321	0.0000000	0.0000000	0.4644732	0.0000000	2
chr9:325671|340321	0.5177424	0.0379100	0.2322366	0.1577607	1
chr9:328022|332478	0.0000000	0.0000000	0.5160298	0.0000000	2

circRNA_coordinates	m6A-CTRL-S1	m6A-CTRL-S2	m6A-BTZ-S1	m6A-BTZ-S2	cluster_group
chr9:33026473|33026988	0.0000000	0.0000000	0.0000000	0.5232528	5
chr9:33932560|33933626	0.0817488	0.0000000	0.6967098	0.0000000	2
chr9:33941647|33953472	0.0000000	0.0000000	0.6967098	0.0000000	2
chr9:33971649|33973235	0.3542448	0.1711511	0.4644732	0.6540660	4
chr9:33986758|33989124	0.0000000	0.5909848	0.0000000	0.0000000	7
chr9:34241183|34242106	0.0000000	0.2567267	0.0000000	0.5232528	5
chr9:35228012|35243361	0.0000000	0.0000000	0.7740446	0.0000000	2
chr9:35295693|35313986	0.4904928	0.0000000	0.0000000	0.0000000	6
chr9:35546427|35548532	0.9264863	0.1711511	0.2322366	0.7848792	6
chr9:36246028|36249394	0.0000000	0.0855756	0.2322366	0.1308132	2
chr9:36581641|36589649	0.0000000	0.0000000	0.4644732	0.0000000	2
chr9:368136|370197	0.0000000	0.0000000	0.4644732	0.0000000	2
chr9:37126309|37126939	18.0119846	7.5306485	13.0052502	13.2121325	1
chr9:37126309|37305711	0.0000000	0.0000000	0.9289464	0.0000000	2
chr9:37126309|37327831	2.4524639	1.7970866	1.8578929	2.7470770	5
chr9:37424842|37426651	0.3542448	0.0000000	0.0000000	0.1308132	6
chr9:6420912|6434173	1.2534815	1.0269066	3.0190760	3.4011430	4
chr9:74481715|74489999	0.9264863	0.8557555	0.0000000	0.3924396	7
chr9:74484999|74489999	0.4632432	0.2567267	0.0000000	0.0000000	7
chr9:74489530|74489999	0.1907472	0.3423022	0.6967098	0.0000000	2
chr9:77631184|77632364	0.6539904	0.6846044	0.6967098	0.9156923	5
chr9:79996892|80022523	0.2997456	0.0000000	0.0000000	0.3924396	5
chr9:88190230|88248289	0.0000000	0.0000000	0.4644732	0.0000000	2
chr9:88200378|88248289	0.2997456	0.0000000	0.4644732	0.7848792	4
chr9:88211277|88248289	1.7167247	0.0855756	0.9289464	1.3081319	1

circRNA_coordinates	m6A-CTRL-S1	m6A-CTRL-S2	m6A-BTZ-S1	m6A-BTZ-S2	cluster_group
chr9:88233898|88248289	3.8966926	2.6528421	2.5546027	4.7092749	5
chr9:88233898|88257858	0.0000000	0.2567267	0.2322366	0.0000000	3
chr9:88284400|88327481	0.4359936	0.0000000	0.2322366	0.2616264	1
chr9:95018962|95032265	0.2452464	0.1564321	0.0000000	0.0580811	6
chr9:95062172|95068112	0.0000000	0.0000000	0.0000000	0.5232528	5
chr9:95477535|95483037	0.0000000	0.0000000	0.6967098	0.1308132	2
chr9:95477535|95485090	0.5177424	0.0000000	0.0000000	0.0000000	6
chr9:95737520|95738991	0.0000000	0.0000000	0.4644732	0.0000000	2
chr9:96233423|96261168	0.1362480	0.4278778	0.0000000	0.5232528	5
chr9:96425918|96427945	0.0000000	0.0000000	0.0000000	0.7848792	5
chr9:98740343|98766983	0.2452464	0.1711511	0.0000000	0.0000000	7
chrUn_gl000220:121605|123082	0.0000000	0.0000000	0.6967098	0.2616264	2
chrX:100356047|100357400	0.2452464	0.0000000	0.2322366	0.0000000	1
chrX:100609705|100611039	0.0272496	0.1564321	0.6967098	0.0000000	2
chrX:118763281|118787003	0.0000000	0.0000000	0.6967098	0.0000000	2
chrX:122829871|122846758	0.0000000	0.0000000	0.6967098	0.0000000	2
chrX:13684436|13698717	0.0000000	0.0000000	0.6967098	0.5232528	4
chrX:13762534|13767652	0.0000000	0.0000000	1.4449762	0.0000000	2
chrX:1404671|1409402	0.1210972	0.0000000	0.4644732	0.0000000	2
chrX:149761067|149787612	0.0000000	0.0000000	0.4644732	0.0000000	2
chrX:149962164|149984551	0.0000000	0.1711511	0.4644732	0.2616264	2
chrX:153296943|153297657	0.0000000	0.0000000	0.4644732	0.0000000	2
chrX:153577425|153577729	0.0000000	0.4278778	0.0102184	0.5232528	5
chrX:153586960|153587351	0.0000000	0.4278778	0.0000000	0.0000000	7
chrX:153763639|153764151	0.0000000	0.0000000	0.4644732	0.0000000	2

circRNA_coordinates	m6A-CTRL-S1	m6A-CTRL-S2	m6A-BTZ-S1	m6A-BTZ-S2	cluster_group
chrX:19610185|19764559	0.0000000	0.0000000	1.1611831	0.0000000	2
chrX:19701941|19713859	0.0000000	0.0000000	0.2322366	0.2616264	4
chrX:19968871|19988416	0.2997456	0.0000000	0.1955432	0.0000000	6
chrX:24179832|24197887	0.2452464	0.1711511	0.0000000	0.1308132	7
chrX:24190832|24197887	5.6406669	1.1980577	6.2703885	3.9243958	1
chrX:24197300|24197887	0.2997456	0.0599029	0.2322366	0.5232528	4
chrX:37245852|37265512	0.0000000	0.0000000	0.4644732	0.1308132	2
chrX:3746547|3747433	0.0000000	0.5990289	0.0000000	0.0000000	7
chrX:53622147|53634660	0.0000000	0.1086810	0.4129167	0.0000000	2
chrX:67272382|67293141	0.0000000	0.0000000	0.9289464	0.0000000	2
chrX:69561649|69607147	0.5722416	0.0855756	0.0000000	0.0000000	6
chrX:70352389|70352586	1.0756779	0.0855756	0.0000000	0.0000000	6
chrX:70587904|70607311	0.0000000	0.0855756	0.0000000	0.3924396	5
chrX:76918871|76920267	0.0272496	0.0000000	0.4644732	0.0000000	2
chrY:15021271|15024974	0.5994912	0.0855756	0.0000000	0.2616264	6
chrY:15024875|15026561	0.0000000	0.0000000	0.6967098	0.0000000	2
chrY:21205049|21206581	0.6267408	0.4278778	1.1611831	0.6540660	1
chrY:21749096|21749393	6.3219069	1.7970866	5.5736787	6.0174069	1
chrY:249339|272325	0.2724960	0.0000000	0.2322366	0.0000000	1
chrY:272140|273067	0.0817488	0.0000000	0.6967098	0.1308132	2
chrY:2821950|2829687	10.7908410	4.4499287	7.1993350	7.5871652	1
chrY:2829115|2829687	2.7794590	0.6846044	0.2322366	1.1773187	6
